# Discovery of pyrimidine-tethered benzothiazole derivatives as novel anti-tubercular agents towards multi- and extensively drug resistant *Mycobacterium tuberculosis*

**DOI:** 10.1080/14756366.2023.2250575

**Published:** 2023-08-30

**Authors:** Loah R. Hemeda, Mahmoud A. El Hassab, Mohamed A. Abdelgawad, Eman F. Khaleel, Marwa M. Abdel-Aziz, Faizah A. Binjubair, Sara T. Al-Rashood, Wagdy M. Eldehna, Mohamed K. El-Ashrey

**Affiliations:** a Department of Medicinal Chemistry, Faculty of Pharmacy, Beni-Suef University, Beni-Suef, Egypt; b Department of Medicinal Chemistry, Faculty of Pharmacy, King Salman International University (KSIU), South Sinai, Egypt; c Department of Pharmaceutical Chemistry, College of Pharmacy, Jouf University, Sakaka, Saudi Arabia; d Department of Pharmaceutical Organic Chemistry, Faculty of Pharmacy, Beni-Suef University, Beni-Suef, Egypt; e Department of Medical Physiology, College of Medicine, King Khalid University, Asir, Saudi Arabia; f The Regional Center for Mycology & Biotechnology, Al-Azhar University, Cairo, Egypt; g Department of Pharmaceutical Chemistry, College of Pharmacy, King Saud University, Riyadh, Saudi Arabia; h Department of Pharmaceutical Chemistry, Faculty of Pharmacy, Kafrelsheikh University, Kafrelsheikh, Egypt; i School of Biotechnology, Badr University in Cairo, Badr City, Egypt; j Department of Pharmaceutical Chemistry, Faculty of Pharmacy, Cairo University, Cairo, Egypt

**Keywords:** Benzothiazole, DprE1, anti-mycobacterial activity, molecular docking, screening

## Abstract

In this study, new benzothiazole–pyrimidine hybrids (**5a**–**c**, **6**, **7a**–**f**, and **8**–**15**) were designed and synthesised. Two different functionalities on the pyrimidine moiety of lead compound **4** were subjected to a variety of chemical changes with the goal of creating various functionalities and cyclisation to further elucidate the target structures. The potency of the new molecules was tested against different tuberculosis (TB) strains. The results indicated that compounds **5c**, **5b**, **12**, and **15** (MIC = 0.24–0.98 µg/mL) are highly active against the first-line drug-sensitive strain of *Mycobacterium tuberculosis* (ATCC 25177). Thereafter, the anti-tubercular activity was evaluated against the two drug-resistant TB strains; ATCC 35822 and RCMB 2674, where, many compounds exhibited good activity with MIC = 0.98–62.5 3 µg/mL and 3.9–62.5 µg/mL, respectively. Compounds **5c** and **15** having the highest anti-tubercular efficiency towards sensitive strain, displayed the best activity for the resistant strains by showing the MIC = 0.98 and 1.95 µg/mL for MDR TB, and showing the MIC = 3.9 and 7.81 µg/mL for XDR TB, consecutively. Finally, molecular docking studies were performed for the two most active compounds **5c** and **15** to explore their enzymatic inhibitory activities.

## Introduction

Tuberculosis (TB) is a lethal transmissible disease and is one of the most contagious killer diseases worldwide^1^^,^^2^. Nearly, 10 million persons get the active stage of TB each year, as described by the World Health Organisation^3^. Isoniazid, ethambutol, rifampicin and streptomycin as first-line drugs have shown effective potency towards *Mycobacterium tuberculosis*^4^^,^^5^. Nevertheless, the TB strains unsusceptible to first line drugs, mostly the multidrug-resistant TB (MDR TB) is a continuous grave problem. The high incidence of a second threat from TB is observed specially in the immune-compromised patients and is known as extensively drug-resistant TB (XDR TB)^6^^,^^7^. Due to the silent form of *Mycobacterium tuberculosis* strains, various current frontline therapeutics have become ineffective and more people are prone to be infected with (MDR-TB) strains every year^8^. Hence, the development of new drugs able to combat this TB epidemic is crucial. As a result, there is a challenge to discover new effective second-line anti-tubercular drugs in the treatment of MDR and XDR TB.

Decaprenylphosphoryl-β-d-ribose-2′-epimerase (DprE1) is an enzyme that is included in the mycobacterial decaprenylphosphoryl-d-arabinose (DPA) biosynthesis. DprE1 is a necessary component for the growth and survival of the mycobacterial cell, so it is considered to be an effective drug target^9–11^. The thymidine monophosphate kinase of *Mycobacterium tuberculosis* (TMPKmt) is another potential target for TB. TMPK is related to the nucleoside monophosphate kinase (NMPK) family that is pivotal for growth in various organisms, involving mycobacteria^12–14^. TMPK enzyme is important in the metabolism of *M. tuberculosis* and has a crucial and unique role in the formation of DNA essential for *M. tuberculosis*. Hence, it was expected that the discovery of a new series of drugs having dual enzyme inhibitory activity against both DprE1 and TMPKmt may get over the problem of the acquired mycobacterial resistance and provide a synergistic activity.

**Figure 1.**
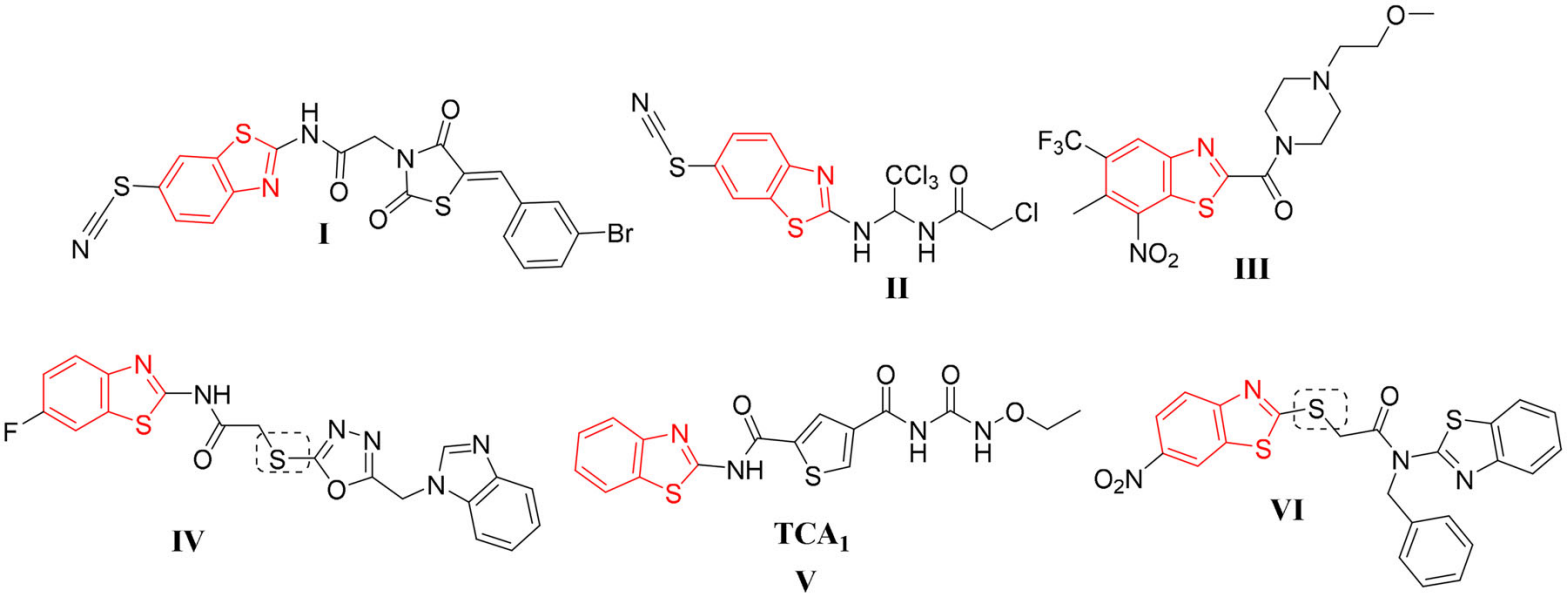
Scaffolds of some reported benzothiazole with potent anti-mycobacterial activity **(I**–**VI)**.

Benzothiazole-based small molecules have a prominent potency towards several microbes^15–18^. In addition, various analogues with benzothiazole moiety such as compounds **I**–**VI** (Figure 1) were effective as remarkable anti-tubercular agents^19–24^. Furthermore, Wang et al. reported the benzothiazole derivative **V** as a selective inhibitor of DprE1. Also, the benzothiazole derivative **VI** was highly selective against DprE1 (Figure 1).

**Figure 2.**
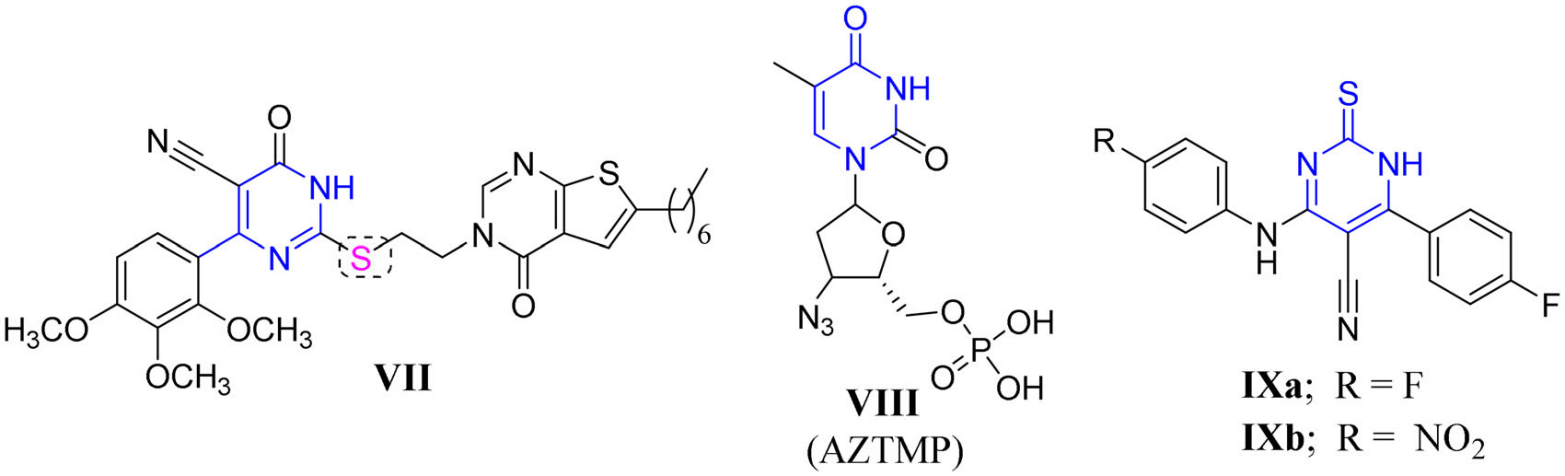
Structures of some reported pyrimidine or thiouracil-based derivatives (**VII**, **VIII**, and **IXa**–**b**) as potent anti-mycobacterial agents.

On the other hand, pyrimidines and their related structures were known as building blocks and privileged scaffolds for the discovery of new antibacterial, antiviral, and antifungal candidates^25^^,^^26^. The related thiouracil molecules were well-reported as promising candidates with promising antimicrobial and antiviral activities^27^^,^^28^. Also, it is worth mentioning that alkaloids and alkaloid-like molecules are important scaffolds in therapeutic drugs for the treatment of TB^29^. Moreover, several pyrimidine-based derivatives demonstrated significant anti-mycobacterial properties towards both *M. tuberculosis* H37Rv and INH resistant clinical strain as shown in compounds **VIIa**–**b**, **VIII**, and **IXa**–**b**^13^^,^^30^^,^^31^ (Figure 2). Interestingly, compound **VIII** was identified as a competitive inhibitor of TMPKmt^12^^,^^13^^,^^32^. Also, compounds **IXa**–**b** emerged as good TMPKmt inhibitors that demonstrated an effective anti-mycobacterial activity^33^ (Figure 2).

**Figure 3.**
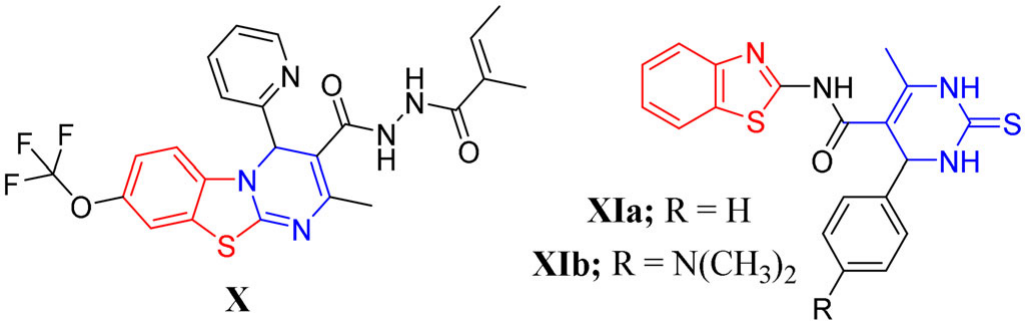
Examples of some benzothiazole derivatives (**X** and **XIa**–**b**) incorporating pyrimidine moiety as potent anti-mycobacterial agents.

Furthermore, hybridisation or fusion of both benzothiazole and pyrimidine moieties into a single entity resulted in diverse series of small molecules endowed with many pharmacological activities against bacteria, fungi, cancer and inflammation in addition to the mycobacteria^13^^,^^33–41^. For example, the 4*H*-pyrimido[2,1-*b*]benzothiazole **X** and the hybridised benzothiazolyl pyrimidine derivatives **XIa**–**b** showed a remarkable activity against TB^13^^,^^14^ (Figure 3). Also, the hybrids **XIa**–**b** were highly selective against DprE1^13^^,^^14^.

**Figure 4.**
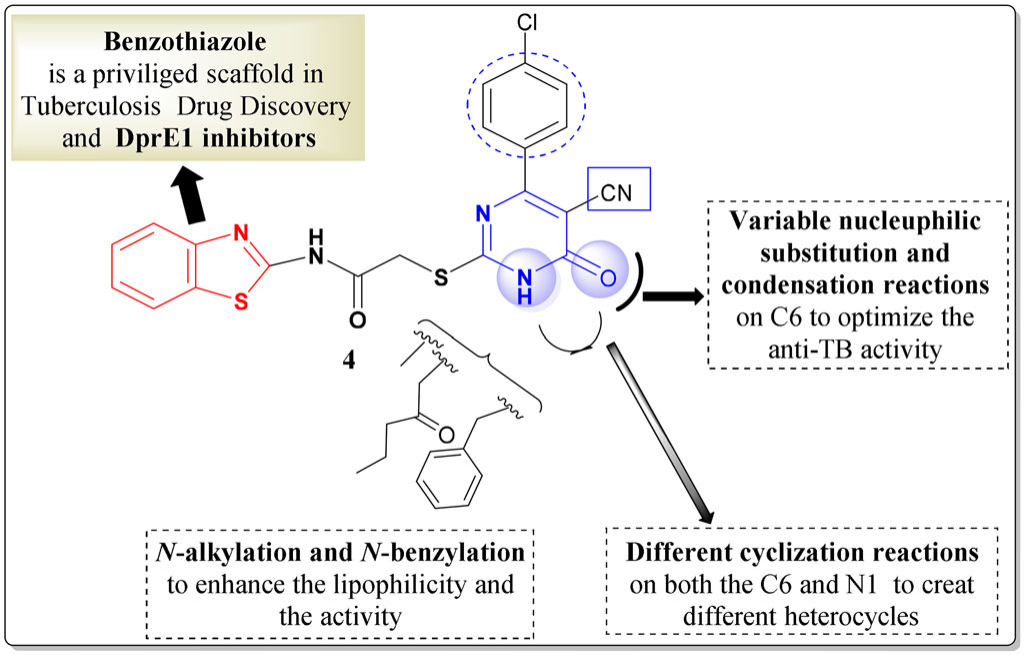
Design of the lead compound **4** and the target hybrids.

Upon consideration of the findings stated above and by using the tethering technique, new series of benzothiazole–pyrimidine conjugates were designed and synthesised. First, the design and synthesis of the lead compound **4** were performed through hybridising the benzothiazole ring with the pyrimidine moiety with thioacetamido linkage (Figure 4).

Thereafter, the effect of different substitutions on N-1 and C-6 positions of the pyrimidine motif was explored to help in studying the SAR of these new hybrids as potential anti-tubercular agents and as dual target enzyme inhibitors (Figure 4). Hence, the N-1 position was substituted with different lipophilic moieties; alkyl, alkylaryl, or ester groups. Moreover, different nucleophilic substitution reactions on C-6 were done in addition to the creation of fused heterocyclic membered rings using both N-1 and C6 positions. The herein described 18 new hybridised benzothiazolyl pyrimidine derivatives (**5**, **6a**–**c**, **7a**–**f**, and **8**–**15**) were then tested for their anti-TB efficiency towards the first line drugs-sensitive strain (ATCC 25177), in addition to the MDR (ATCC 35822), and XDR (RCMB 2674) *M. tuberculosis* strains. Subsequently, the dual enzyme inhibitory activity of the most active compounds was explored using the molecular docking studies depending on the ligand–protein interactions.

## Results and discussion

### Chemistry

**Scheme 1.**
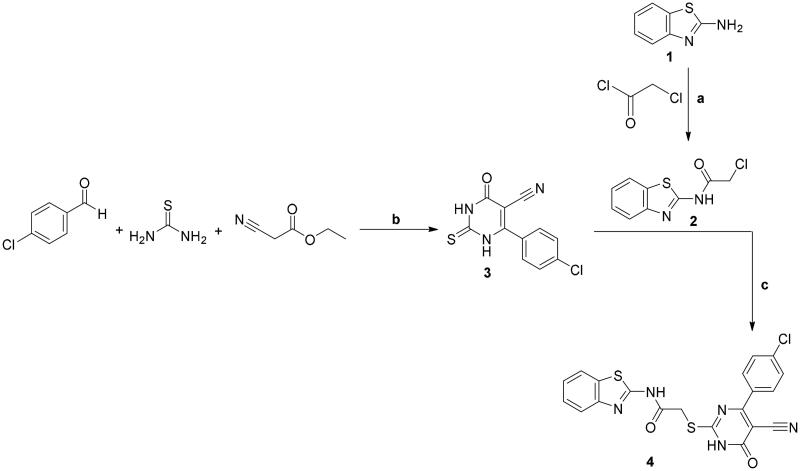
Synthesis of compounds **2**–**4**; conditions and reagents: (a) dry benzene, anhydrous K_2_CO_3_, reflux for 12 h; (b) absolute ethyl alcohol, anhydrous K_2_CO_3_, reflux for 10–12 h; (c) dry acetone, anhydrous K_2_CO_3_, reflux for 8–10 h.

**Scheme 2.**
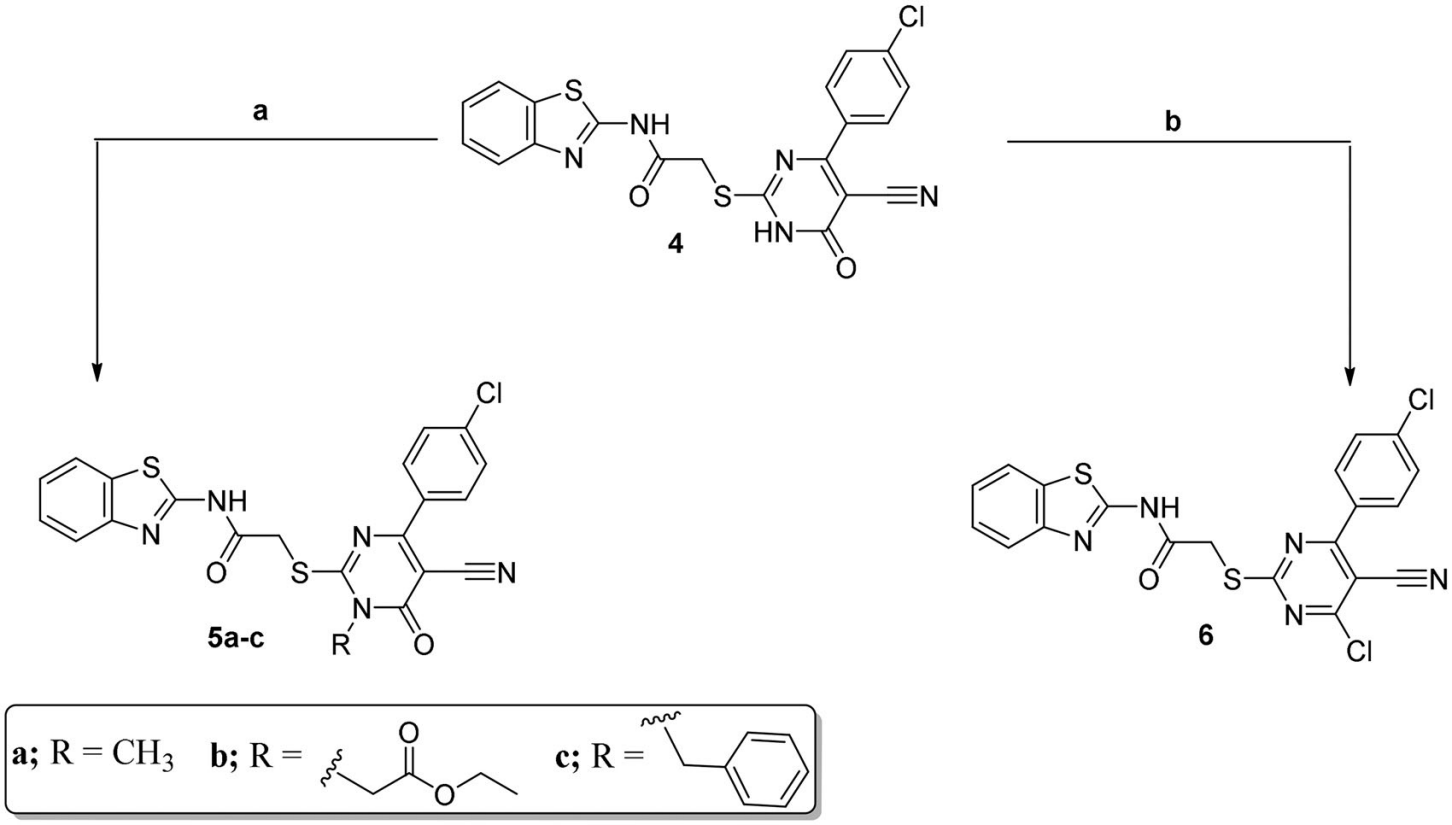
Synthesis of **5a**–**c** and **6**; conditions and reagents: (a) dry DMF, CH_3_I/C_6_H_5_CH_2_Cl/ClCH_2_COOC_2_H_5_, anhydrous K_2_CO_3_, reflux for 12 h; (b) POCl_3_, reflux for 3 h.

**Scheme 3.**
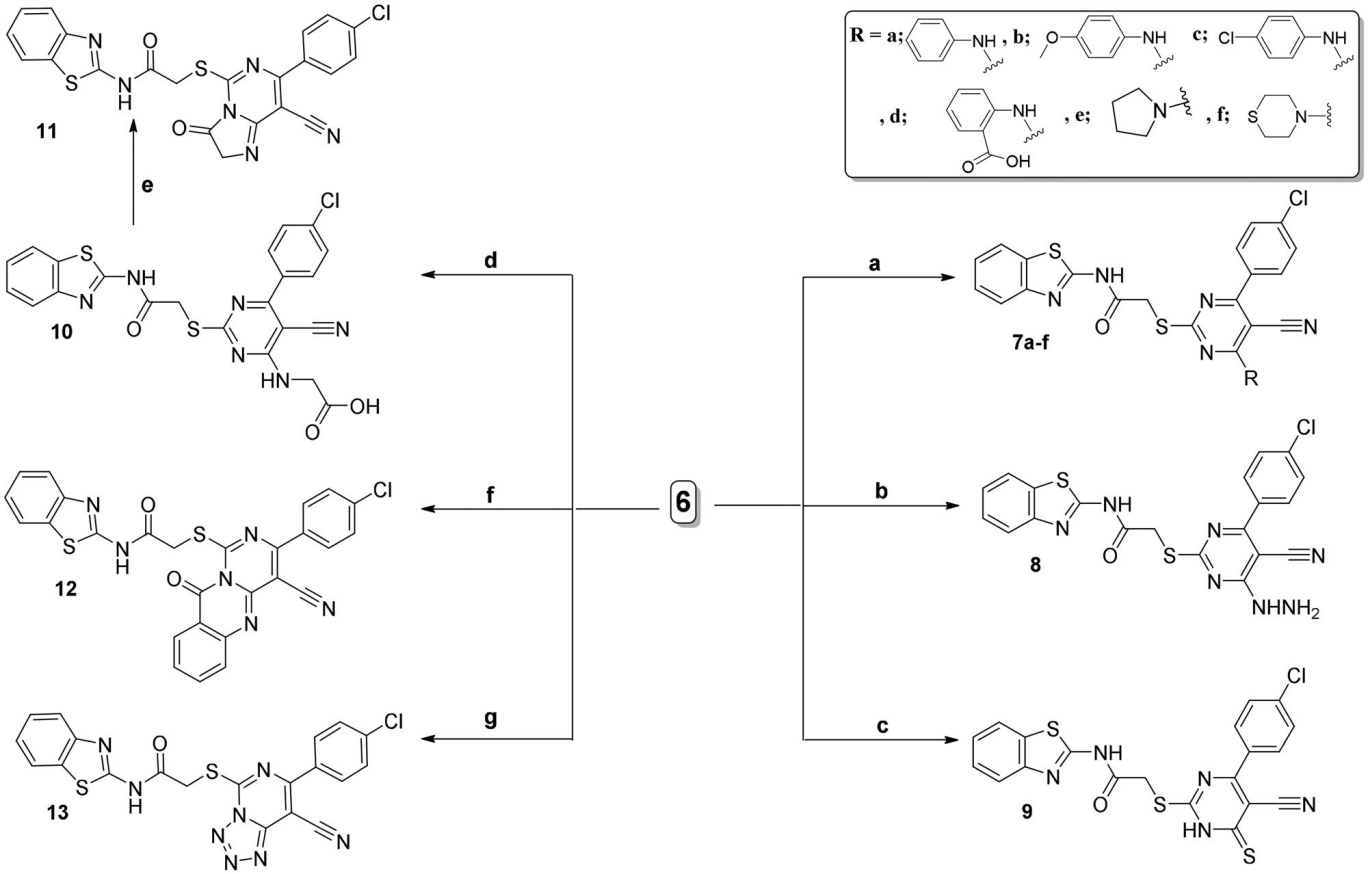
Synthesis of compounds **7a**–**f** and **8**–**13**; conditions and reagents: (a) absolute ethyl alcohol, primary or secondary amine, TEA, room temperature for 24 h, then the heating under reflux for 6–12 h; (b) hydrazine hydrate, abs. ethanol, reflux, 6 h; (c) thiourea, abs. ethanol, reflux, 6 h; (d) n-butanol, glycine, reflux for 3 h; (e) reflux for 2 h with acetic anhydride; (f) fusion with the anthranilic acid in the oil bath at 190 °C, for 2 h; (g) glacial acetic acid, sodium azide, reflux for 3 h.

**Scheme 4.**
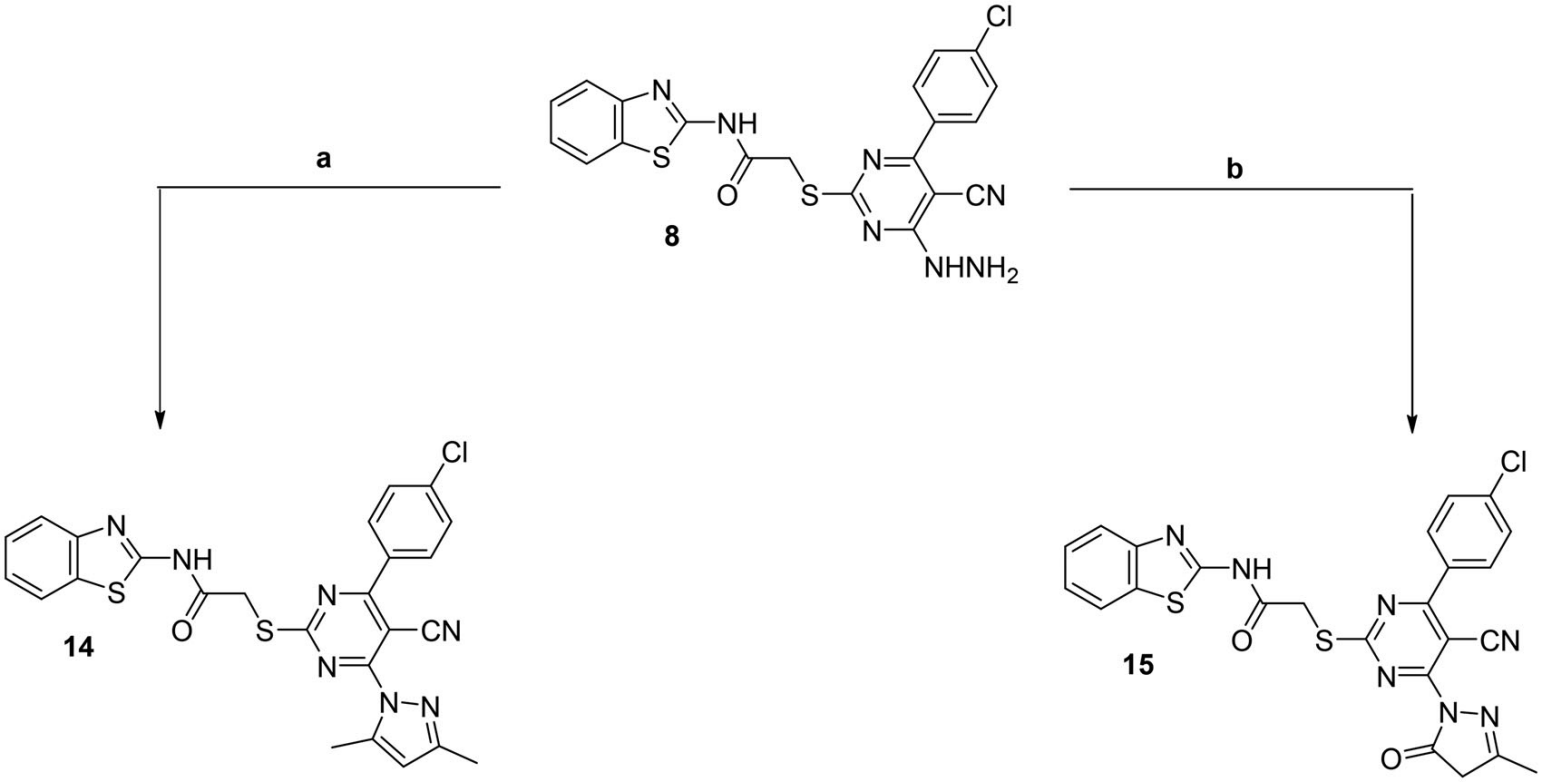
Synthesis of **14** and **15**; conditions and reagents: (a) glacial acetic acid and acetyl acetone, reflux for 6 h. (b) Reflux with the ethyl acetoacetate in NaOC_2_H_5_, for 4 h.

A new series of benzothiazole–pyrimidine hybrids **5**, **6a**–**c**, **7a**–**f**, and **8**–**15** were described in the drawn Schemes 1–4. The elemental analyses with the spectral data confirmed the structures of the new target compounds. The lead compound **4** was synthesised through the reaction between the two key intermediates; the 2-chloroacetamide derivative **2** and the 6-(4-chlorophenyl)-tetrahydropyrimidine-5-carbonitrile derivative **3**. The synthesis of compound **2** was performed through the chloroacetylation of the 2-aminobenzothiazole **1** with the chloroacetyl chloride using equimolar quantities^42^. The 6-(4-chlorophenyl)-tetrahydropyrimidine-5-carbonitrile derivative **3** was prepared by using equimolar quantities of ethyl cyanoacetate, 4-chlorobenzaldehyde, and thiourea^43–45^. Then, the pyrimidine derivative **3** was reacted with the chloroacetamide derivative **2** to give the target compound **4**^46^ (Scheme 1).

The pyrimidine-bearing benzothiazole hybrid **4** was subjected to a number of reactions on the N-1 and/or the C6 reactive sites of the pyrimidine moiety. The N-alkylated target compounds **5a**–**c**, were synthesised using alkylating agents such as CH_3_I, ClCH_2_COOC_2_H_5_, and C_6_H_5_CH_2_Cl, respectively, using an anhydrous K_2_CO_3_ in DMF. The 4-chloropyrimidine derivative **6** was produced through the reflux of hybrid **4** with excess POCl_3_ (Scheme 2).

IR and NMR (^1^H and ^13^C) spectral data of molecules **5a**–**c** and **6** were matched with the suggested structures. Noticeably, the proton spectra of compounds **5a**–**c** displayed new singlets at *δ* 3.82, 5.34, and 5.64 ppm due to the *N*-CH_3_, *N*-CH_2_-COOEt, and *N*-CH_2_-C_6_H_5_ protons, respectively. In addition, **5b** displayed triplet signal at *δ* 0.86 ppm and a quartet signal at *δ* 1.45 ppm related to CH_3_ and OCH_2_ protons, respectively. Also, the ^1^H NMR spectrum for **5c** showed an increase in the aromatic protons integration.

Furthermore, a new series of benzothiazole derivatives was synthesised by the nucleophilic substitution reactions of the chloro derivative **6** with variable nucleophiles. The reaction between compound **6** and different amine derivatives (primary or secondary) in absolute ethyl alcohol using the triethylamine (TEA) yielded the compounds **7a**–**f**. ^1^H NMR spectra of **7a**–**d** showed the exchangeable singlets of NH proton of the anilino moiety around *δ* 9.72 to 12.64 ppm, whereas, the singlet signals for the OCH_3_ and COOH group protons appeared at *δ* 3.67 and 11.94 ppm for **7b** and **7d**, respectively. The proton spectrum of compound **7e** displayed two triplet signals at *δ* = 1.80, 3.70 ppm related to the eight protons of the 2-methyl (CH_2_) in addition to the 2N-methyl (N-CH_2_) groups, respectively. The eight protons of CH_2_-N-CH_2_ and CH_2_-S-CH_2_ of compound **7f** were confirmed through the ^1^H NMR spectrum by showing two broad signals at *δ* 2.68 and 4.14 ppm, respectively.

Further, reflux of 98% hydrazine hydrate with chloropyrimidine derivative **6** in absolute ethyl alcohol gave the hydrazine-pyrimidine hybrid **8**. Confirming of the hybrid **8** was done through its IR spectrum through showing three stretching bands at 3376 and 3296 and 3187 cm^–1^ attributed to the stretching vibrations of NH and NH_2_, respectively. In addition, two singlets at *δ* = 5.04 and 12.47 ppm were observed by its proton spectrum, that were matched with the protons of NH_2_ and NH, respectively. The replacing of the Cl atom in **6** with mercapto functionality was performed upon using thiourea and reflux in boiling ethanol to give **9**. The dissociation of the non-stable isothiouronium salt was considered to be the reason for the formation of compound **9**. The IR spectrum of the C═S functionality displayed an absorption band at 1278 cm^–1^. Proton and carbon NMR spectra were also consistent with its structure.

Also, the chloro derivative **6** was used as a reactive key precursor for ring cyclisation giving imidazopyrimidine **11**, quinazolinopyrimidine **12**, and tetrazolopyrimidine **13** derivatives. First, non-cyclised glycine bearing derivative **10** is formed by the reaction of the chloropyrimidine derivative **6** with glycine in butanol. Thereafter, the heating of compound **10** with acetic anhydride led to the intramolecular cyclisation giving the corresponding imidazopyrimidine derivative **11**.

For compound **10**, its IR spectrum displayed the carboxylic C═O vibrating at 1715 cm^–1^. Also, **10** was confirmed with its proton spectrum that displayed D_2_O exchangeable singlets at *δ* 8.38 and 12.69 ppm related to the amino (NH) and hydroxyl (OH) protons of glycine motif. The ^1^H NMR spectrum confirmed the structure of **11** by displaying a signal that is singlet at *δ* 4.04 ppm due to protons of CH_2_ of the imidazole moiety. Also, the proton spectrum lacked any D_2_O exchangeable protons for the NH and OH protons of glycine motif.

Further, cyclic pyrimidoquinazoline derivative **12** was yielded through the fusion between the chloro derivative **6** and the anthranilic acid in the oil bath. The ^1^H NMR spectrum of **12** lacked any deuterium oxide exchangeable singlets due to NH and OH protons of anthranilic acid confirming their contribution in the cyclisation process giving the cyclised quinazoline derivative **12**. Also, ^1^H NMR spectrum of **12** displayed increasing in the aromatic protons integration confirming its structure. Regarding the tetrazolo derivative **13**, its synthesis was performed through the reflux of the 4-chloro pyrimidine **6** with NaN_3_ in glacial CH_3_COOH (Scheme 3).

Compound **8** was utilised as another key precursor for further heterocyclisation affording pyrazolo ring fused to the pyrimidine nucleus (derivatives **14** and **15**). Upon reacting acetylacetone with hydrazine derivative **8** in refluxing glacial acetic acid, 3,5-dimethyl-*N*-substituted pyrazole **14** was afforded. Moreover, the hydrazine derivative **8** was heated under reflux with ethylacetoacetate in sodium-ethoxide solution in absolute ethanol to afford the corresponding pyrazolone derivative **15** (Scheme 4).

Compounds **14** and **15** were revealed by the IR and ^1^H NMR spectral data through the disappearance of both the forked band and the exchangeable singlet signal characterising the NH_2_ of the hydrazine functionality. For compound **14**, ^1^H NMR spectrum showed two singlet signals at *δ* = 2.52 and 2.64 ppm matched with the two CH_2_ groups of the pyrazole moiety and showed a signal that was singlet at *δ* = 7.53 ppm related to the 4-*H*-pyrazole moiety. While for compound **15**, the IR spectrum showed the carbonyl stretching band of pyrazolone ring vibrating at 1696 cm^–1^. Also, ^1^H NMR spectrum displayed two bands at *δ* 2.42 and 4.04 ppm due to methyl (CH_3_) and methylene (CH_2_) groups of pyrazolone ring, respectively.

### Biological evaluations

#### Anti-tuberculosis effect

The anti-TB effects of **5a**–**c**, **6**, **7a**–**f**, and **8**–**15** besides the lead compound **4** were tested for the sensitive strain of *M. tuberculosis* (ATCC 25177) by the assay of Microplate Alamar Blue and using INH as the reference. Table 1 describes the results and the minimum inhibitory concentration (MIC) was used for their expression. SAR depended on the obtained results and on the comparison of the new compounds with the lead compound **4** in addition to the reference INH.

**Table 1.** MIC in µg/mL for **4**, **5a**–**c**, **6**, **7a**–**f**, and **8**–**15**, as well as INH against the sensitive *M. tuberculosis* (ATCC 25177).

Comp.	MIC (µg/mL)
	*M. tuberculosis* (ATCC 25177)
**4**	3.9
**5a**	1.95
**5b**	0.98
**5c**	0.24
**6**	1.95
**7a**	15.63
**7b**	125
**7c**	125
**7d**	31.25
**7e**	7.81
**7f**	7.81
**8**	3.9
**9**	3.9
**10**	15.63
**11**	31.25
**12**	0.98
**13**	62.5
**14**	7.81
**15**	0.48
**St. Isoniazid**	0.24

As shown in Table 1, the majority of the tested compounds displayed an effective anti-TB activity by having MIC in the range of 0.24–7.81 µg/mL. Compound **5c** was the highest active derivative by having MIC of 0.24 µg/mL and showed an equal potency to INH and showed a potency with about 16-fold of the lead compound **4** (MIC = 3.9 µg/mL). In addition, compounds **5b**, **12**, and **15** revealed high potent activity by having MIC values of 0.98, 0.98, and 0.48 µg/mL, respectively. Whereas a moderate activity was observed for **4**, **5a**, **6**, **7e**, **7f**, and **8**, **9**, and **14** by having MIC values in the range from 1.95 to 7.81 µg/mL. Regarding the other derivatives, a slight to fair anti-TB activity was observed by having MIC in the range from 15.63 to 125 µg/mL

The lead benzothiazolyl pyrimidine hybrid **4** showed a moderate effect with MIC of 3.9 µg/mL relative to INH. Introducing lipophilic moieties on N-1 of pyrimidine ring, shown by compounds **5a**–**c**, led to more effective anti-tubercular activity with MIC in the range of 0.24–1.95 µg/mL in comparison to compound **4** having MIC of 3 µg/mL. Generally, the *N*-benzyl counterpart **5c** demonstrated the highest activity by having MIC of 0.24 µg/mL not only among the *N*-substituted derivatives **5a**–**c**, but also among all the synthesised molecules herein. The *N*-ethyl acetate bearing analogue **5b** was the second active drug of the *N*-substituted derivatives (MIC = 0.98 µg/mL) compared to **4** and INH. Compound **5a** bearing methyl group displayed the lowest activity in this series (MIC = 1.95 µg/mL) compared to **4** and INH. Clearly, it was assumed that the increasing of the lipophilic side chain on N-1 of pyrimidine ring increased the bioavailability and penetration and was mostly advantageous for the anti-tubercular activity. Hence, the activity of the *N*-substituted derivatives decreased in the order of N-CH_2_-C_6_H_5_ > N-CH_2_-CO-C_2_H_5_ > N-CH_3_.

The replacement of the carbonyl group with the chloro group in compound **6**, increased the anti-tubercular effect (MIC = 1.95 µg/mL) compared to **4** by about twofold. Unexpectedly, the presence of an aniline moiety directly attached to the pyrimidine ring as in compounds **7a**–**d** diminished the activity (MIC = 15.63–125 µg/mL) relative to compound **4** and the reference INH. Concerning the impact of substitution of the aniline moiety within hybrids **7b**–**d**, the anti-tubercular activity decreased by 2–8 folds (MIC = 125, 125, and 31.25 µg/mL, respectively) compared to the unsubstituted analogue **7a** (MIC = 15.63 µg/mL). It was noticed that the *para* substitution of the anilino motif with a methoxy (analogue **7b**) or chlorine atom (analogue **7c**) had the same impact (MIC = 125 µg/mL) and resulted in a diminished activity. However, the slightly better activity of **7d** (MIC = 31.25 µg/mL) having the carboxylic acid functionality at the *ortho* site of the aniline ring, indicated that the *ortho* substitution could be more favourable than the *para* substitution. Overall, the steric factors of aniline derivatives **7a**–**d** may be the reason for the visible decrease in the potency and hence the unsubstituted derivative **7a** was more favourable than the substituted ones **7b**–**d**. Conversely, the introduction of alicyclic amino groups (pyrrolidine and thiomorpholine) in compounds **7e** and **7f** manifested a better activity (MIC = 7.81 and 7.81 µg/mL, respectively) than anilino derivatives **7a**–**d** (MIC = 125, 125, and 15.63 µg/mL, respectively). However, tethering the pyrimidine motif with pyrrolidine **7e** and thiomorpholine **7f** did not show an increase in the anti-tubercular activity compared to compound **4** and INH. Furthermore, the present study revealed that the presence of the hydrazino group in compound **8** or thione group in compound **9** at C-6 of the pyrimidine ring had not affected the anti-tubercular activity by having MIC of 3.9 µg/mL that was similar to **4**.

It is worth to mention that neither the open aminoacetic acid derivative **10** nor its cyclised imidazolo derivative **11** showed an increase in the activity (MIC = 15.63 and 31.25 µg/mL, respectively) compared to the lead compound **4** and INH. Noticeably, the open aminoacetic acid derivative **10** has twice the activity of its cyclised imidazole derivative **11**. Comparably, the fusion of the quinazolino moiety to the pyrimidine ring in **12** showed a remarkable increase in the activity (MIC = 0.98 µg/mL) by about fourfold compared to compound **4**. Unlike the cyclised imidazolo derivative **11** and its open counterpart **10**, the pyrimidoquinazoline derivative **12** (MIC = 0.98 µg/mL) showed more than 32-fold increase in the activity in comparison to its open anthranilic acid analogue **7d** (MIC = 31.25 µg/mL). Fusion of pyrimidine ring with tetrazole moiety in compound **13** diminished the activity (MIC = 62.5 µg/mL) compared to compound **4** and INH. From the obtained results, it could be concluded that the fusion of pyrimidine ring with five membered heterocyclic rings as in compounds **11** and **13** is less favourable than that with higher ones as in compound **12**. Both pyrazole **14** and pyrazolone **15** derivatives showed higher activity (MIC = 7.81 and 0.48 µg/mL, respectively) than their hydrazino counterpart **8**, highlighting that the heterocyclisation of the hydrazine derivative **8** is favourable. Interestingly, the pyrazolone **15** was the second most active newly synthesised compound after **5c** and showed half the activity (MIC = 0.48 µg/mL) of INH.

Finally, different factors influenced the activity of the newly synthesised molecules against different strains of TB described as *N*-substitution, nucleophilic substitution, or heterocyclisation at position 6 of the pyrimidine ring as well as ring cyclisation at N-1 and C-6. It was found that raising the lipophilic character for the pyrimidine moiety through *N*-1 alkyl substitution was more favourable for activity. Collectively, the *N*-benzyl derivative **5c** showed the best activity that may be attributed to its high lipophilicity, whereas the pyrazolone derivative **15** was the second most active derivative. On the other hand, the anilino derivatives **7b** and **7c** were the least active ones followed by the tetrazole derivative **13**.

#### Anti-tuberculosis effect against multidrug resistant M. tuberculosis (ATCC 35822)

In this work, the anti-tubercular activity for the most potent members in the previous assay (**4**, **5a**–**c**, **6**, **7e**, **7f**, **8**, **9**, **12**, **14**, and **15**) was further assessed against MDR strain using MABA protocol. The tested MDR strain (ATCC 35822) demonstrates a resistance towards isoniazid, cycloserine, kanamycin, and rifampin.

Interpreting the obtained results for the target molecules demonstrated that most of the evaluated derivatives displayed good effect by having MIC values of 0.98–62.5 µg/mL (Table 2). Hybrids **5c** and **15** with the most anti-TB efficiency for the sensitive *M. tuberculosis* (ATCC 27294), exhibited potent effect against the MDR strain (ATCC 35822) by having MIC values of 0.98 and 1.95 µg/mL, respectively. They also showed more than 128- and 64-fold rising in the potency in comparison with the lead hybrid **4** having MIC value equal to 125 µg/mL. Furthermore, hybrids **5a**, **5b**, and **12** exerted an excellent action by having MIC of 7.81 µg/mL, whereas derivatives **6** produced a good action by having MIC of 31.25 µg/mL. Additionally, compounds **7e**, **8**, and **9** showed a moderate action by having MIC of 62.5 µg/mL.

**Table 2.** MIC in µg/mL of **4**, **5a**–**c**, **6**, **7e**, **7f**, **8**, **9**, **12**, **14**, and **15** against the MDR TB (ATCC 35822) and XDR TB (RCMB 2674) strains.

Comp.	MIC (µg/mL)	
	*M. tuberculosis* (MDR) (ATCC 35822)	*M. tuberculosis* (XDR) (RCMB 2674)
**4**	125	<125
**5a**	7.81	31.25
**5b**	7.81	31.25
**5c**	0.98	3.9
**6**	31.25	62.5
**7e**	62.5	NA
**7f**	125	NA
**8**	62.5	<125
**9**	62.5	125
**12**	7.81	31.25
**14**	NA	NA
**15**	1.95	7.81
**St. Isoniazid**	>125	>125

#### Anti-tuberculosis activity against extensively drug-resistant M. tuberculosis (RCMB 2674)

Furthermore, the anti-TB effect of **4**, **5a**–**c**, **6**, **7e**, **7f**, **8**, **9**, **12**, **14**, and **15** against XDR strain is shown in Table 2. It was observed that the results of tested molecules showed a visible correlation for MDR and XDR strains. Many of the tested derivatives were observed to be of a good effect by having MIC values in the range of 3.9–62.5 µg/mL. Hybrids **5c** and **15** having the most anti-TB efficiency against sensitive and MDR strains, showed also an excellent activity against XDR by having MIC values of 3.9 and 7.81 µg/mL, respectively, and by showing more than 16–32-fold enhancing in the efficiency in comparison with the lead hybrid **4**. Moreover, compounds **5a**, **5b**, and **12** showed a moderate activity by having MIC of 31.25 µg/mL. In contrast, the derivative **6** produced a weak activity by having MIC of 62.5 µg/mL.

### Molecular docking

In structural molecular biology and computer-assisted drug design, molecular docking is a critical tool. The goal of ligand–protein docking is to predict the most likely binding mode(s) of a ligand with a known three-dimensional structure of a protein. Successful docking methods effectively search high-dimensional spaces and employ a scoring function that correctly ranks candidate dockings^47^. In the present work, two of the most important biological targets in *Mycobacterium tuberculosis* were selected to perform the molecular docking study; decaprenylphosphoryl-d-ribose oxidase (DprE1) (pdb ID: 4KW5)^48^ and TMPKmt (pdb ID: 1W2H)^49^. Two synthesised derivatives were chosen to test their binding affinity with the two target enzymes (**5c** and **15**). The two tested compounds showed good binding scores to both enzymes compared to the score of the co-crystallised ligands (Table 3).

**Table 3.** Binding energy results in kcal/mol for the tested hybrids versus the co-crystallised ligands.

Compound	DprE1 (pdb: 4KW5) Energy score (kcal/mol)	TMPKmt (pdb: 1W2H) Energy score (kcal/mol)
**5c**	−21.5	−19.7
**15**	−19.8	−18.4
**The co-crystall**ise**d ligand**	−20.3	−17.9

**Figure 5.**
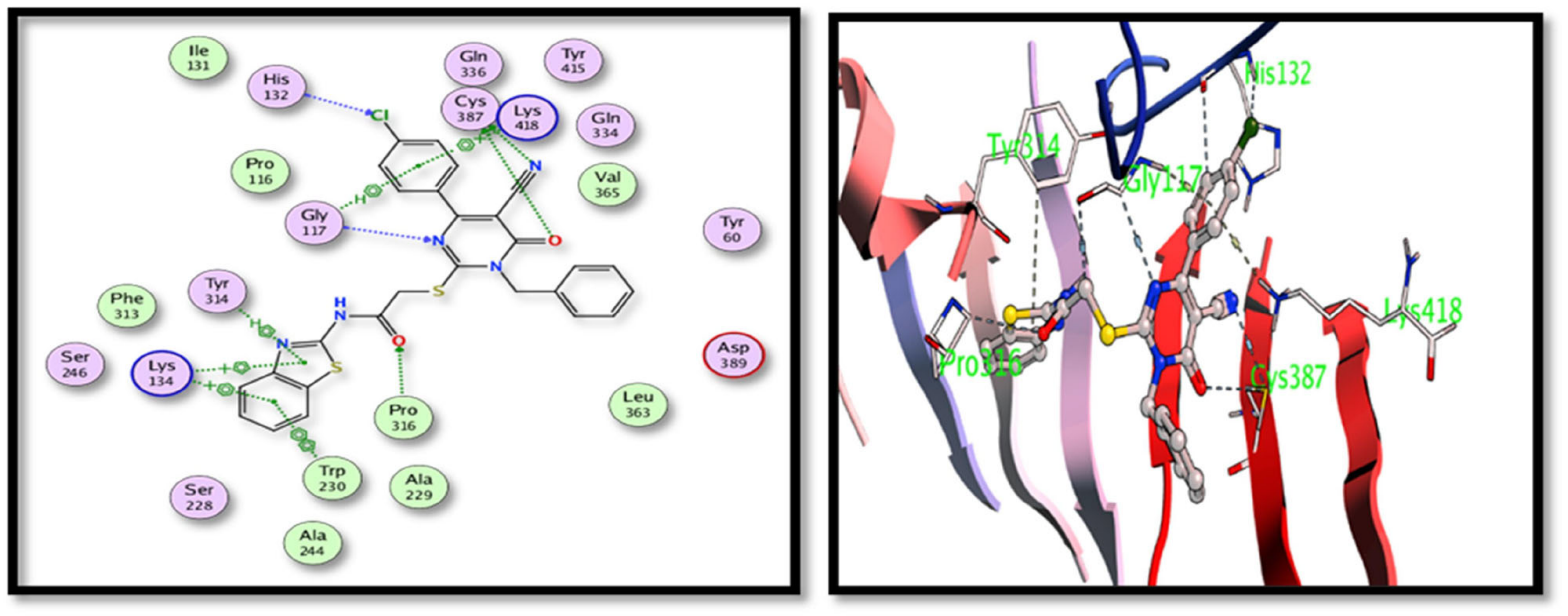
2D and 3D interactions of compound **5c** with the binding position of **DprE1** enzyme.

**Figure 6.**
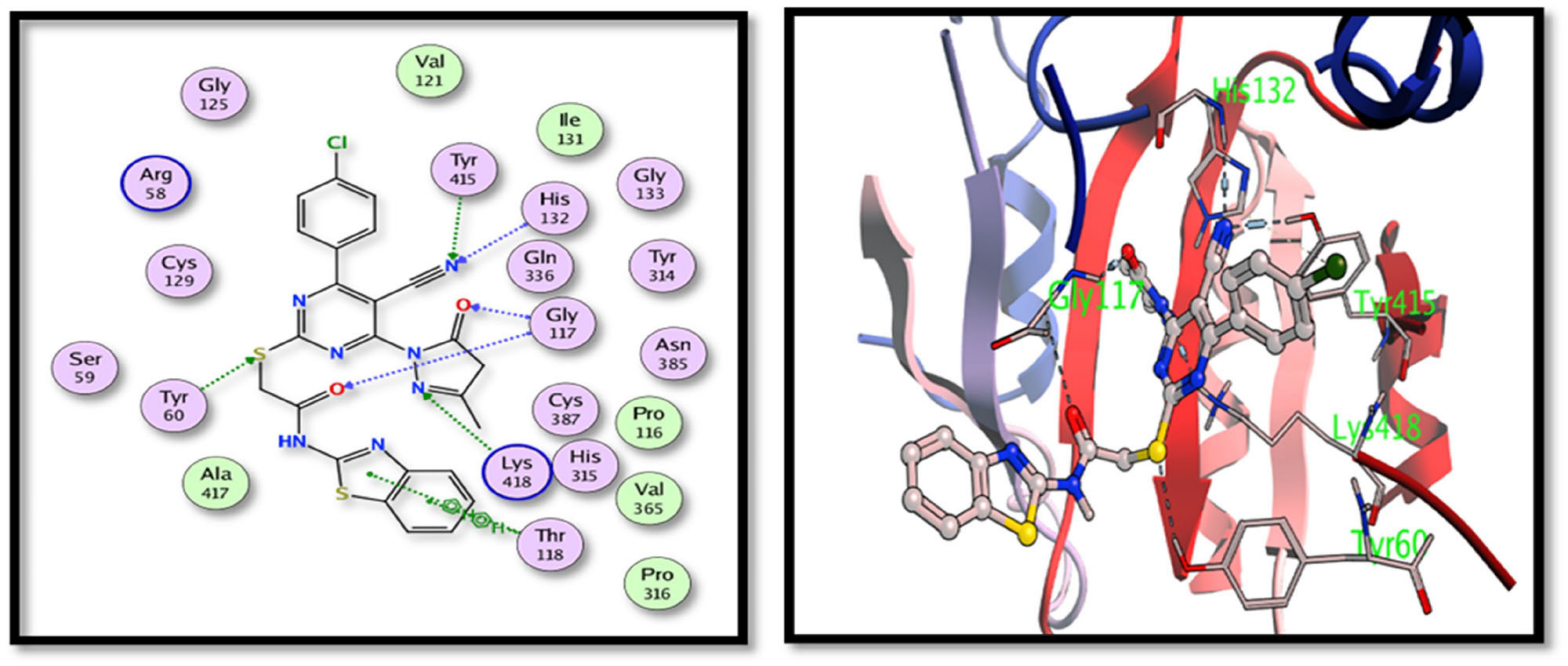
2D and 3D interactions of compound **15** with the binding position of **DprE1** enzyme.

For DprE1 binding site, **5c** derivative showed good binding interactions through the main binding site amino acid residues. Several hydrogen bonding interactions were formed between the main functional groups in the tested **5c** compound, nitrile N with Cys387, carbonyl oxygens with Cys387 and Pro316 and pyrimidone N with Gly117 with additional halogen bonding of Cl with His132 (Figure 5). On the other hand, compound **15** showed also good binding interactions through hydrogen bonding of nitrile N with His132 and Tyr415, carbonyl oxygens with Gly117, pyrazolone with Lys418 and S with Tyr60 (Figure 6).

**Figure 7.**
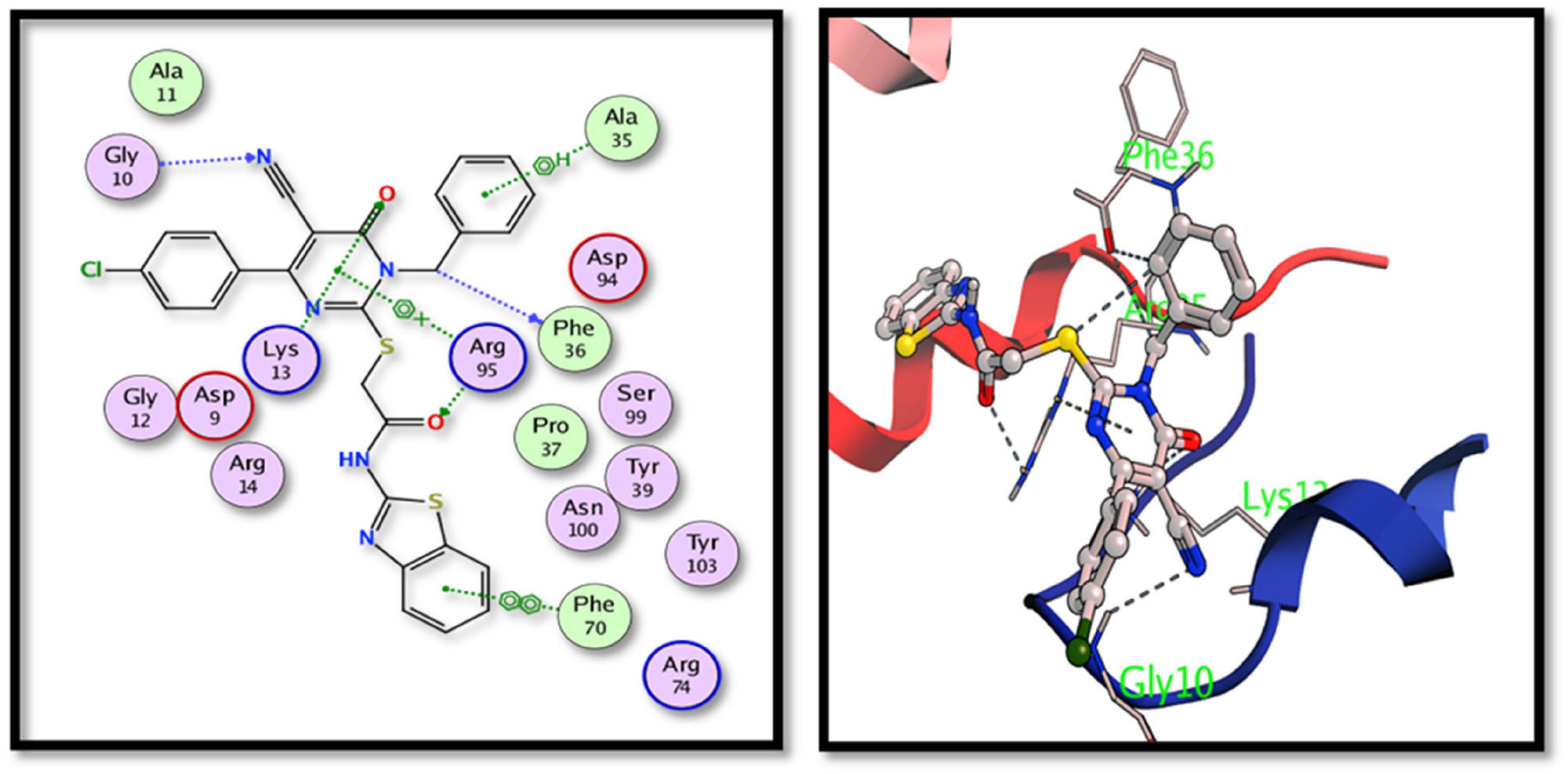
The 2D and 3D interactions of compound **5c** with the binding position of **TMPKmt** enzyme.

**Figure 8.**
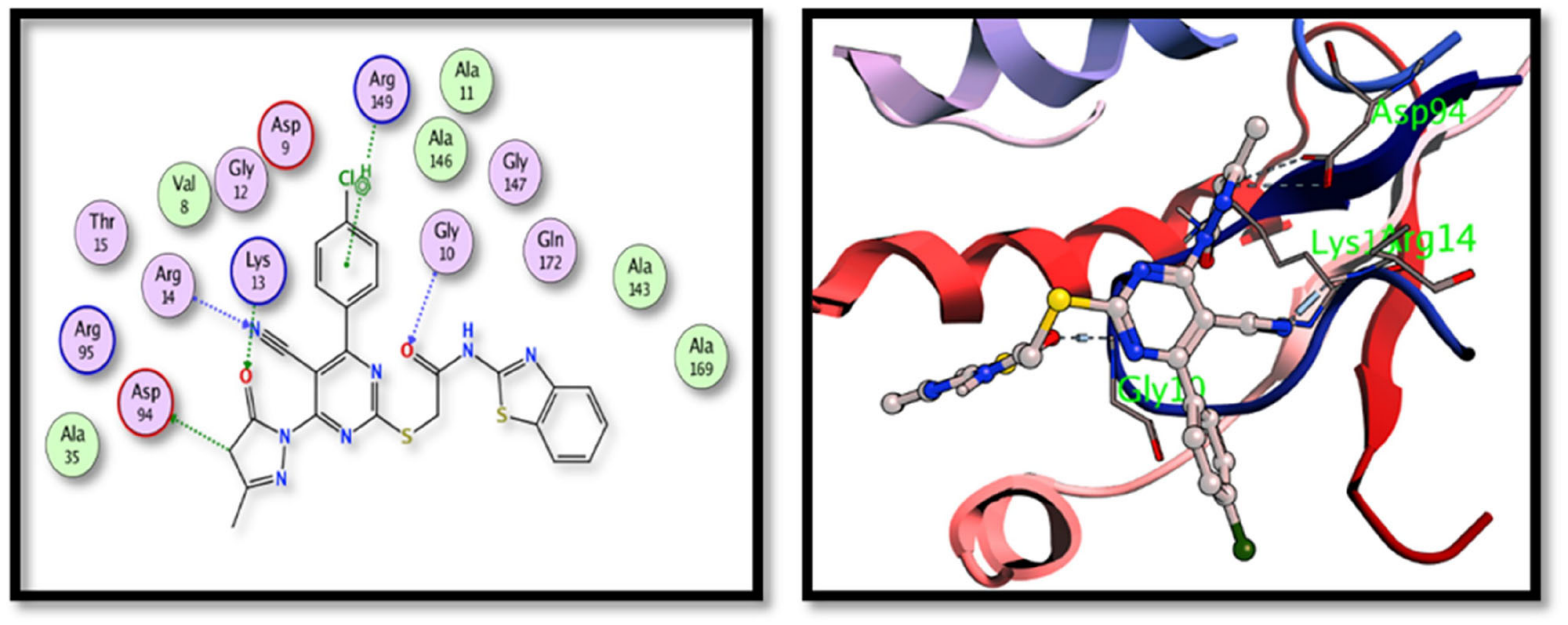
The 2D and 3D interactions for the compound **15** with the binding position of **TMPKmt** enzyme.

The two tested derivatives showed also a good binding affinity to TMPKmt enzyme. Both derivatives showed hydrogen bond interactions with the key amino acids Gly10 and Lys13. Compound **5c** binds to Gly10 (nitrile N), Lys13 and Arg95 (carbonyl oxygens) (Figure 7) while compound **15** showed interactions with Arg14 (nitrile N), Gly10 and Lys13 (carbonyl oxygens) (Figure 8).

## Conclusions

In the present research, variable benzothiazolyl pyrimidine hybrids **4**, **5a**–**c**, **6**, **7a**–**f**, and **8**–**15** were synthesised. The activity against the different TB strains namely sensitive *M. tuberculosis* (ATCC 25177), MDR strain of *M. tuberculosis* (ATCC 35822) and the XDR strain of *M. tuberculosis* (RCMB2674) were tested for the 18 newly synthesised derivatives in addition to the lead compound **4**. Compounds **5c** and **15** showed the best anti-TB effect in this work by having MIC = 0.24 and 0.48 µg/mL, showing minimal resistance against MDR strain by having MIC = 0.98 and 1.95 µg/mL and against XDR strain by having MIC = 3.9 and 7.81 µg/mL, respectively. The SAR analysis highlighted that enhancing the lipophilic nature regarding the pyrimidine ring through *N*-1 alkyl substitution was more favourable for activity, whereas introducing the amino moieties in compounds **7a**–**d** through the nucleophilic substitution, produced a diminished anti-mycobacterial activity. Finally, the two most active compounds **5c** and **15** showed good binding scores to DprE1 and TMPKmt enzymes compared to the score of the co-crystallised ligands.

## Experimental work

### Chemistry

#### General methods regarding chemistry

No additional purification was done for the used commercial solvents and chemical agents that were reagent-grade. The apparatus of IA 9100 MK digital melting point griffin was used to measure the total melting points and they were not corrected and the open capillary tubes were used for measuring. At the Micro Analytical Center, Science College, in Cairo, Egypt, the IR spectral data were done on Bruker Vector 22 FTIR Spectrometer. At Pharmacy College, at Beni-Suef University, in Egypt, the proton and carbon spectral data were made on a Bruker Avance III at 400 MHz and at 100 MHz for NMR (^1^H and ^13^C), respectively. At Mycology and Biotechnology Regional Center, at the University of Al Azhar, in Cairo, Egypt, inlet-part to single quadrupole mass analyzer was used to record the mass spectra. Compounds **2**–**4** were synthesised based on the reported procedures^42–46^. Compound **1** can be obtained commercially.

#### General method for synthesis of N-(benzo[d]thiazol-2-yl)-2-((4-(4-chlorophenyl)-5-cyano-1-substituted-6-oxo-1,6-dihydropyrimidin-2yl)thio) acetamide derivatives (5a–c)

A mixture of 5 mmol of **4** (2.26 g) and 73 mmol of anhydrous K_2_CO_3_ (1 g) in 20 mL of dry DMF was mixed with 20 mmol of the suitable alkyl halide. The mixture underwent 12 h reflux followed by cooling and pouring on cold H_2_O. The precipitate was collected through filtration, left to dry and then recrystallised using CHCl_3_/C_2_H_5_-OH mixture.

##### N-(benzo[d]thiazol-2-yl)-2-((4-(4-chlorophenyl)-5-cyano-1-methyl-6-oxo-1,6 dihydro pyrimidin-2-yl)thio)acetamide (5a)

Yield 73%; melting point: 167–170 °C. IR (*ʋ*_max_/cm^–1^): 3424 (stretching NH), 3197 (aromatic CH), 2921 (aliphatic CH), 2212 (C≡N), 1694 (C═O), 1579 (C═N), 1530 (C═C). ^1^H NMR (DMSO-*d*_6_) *δ* ppm: 3.85 (s, 3H, *N*-CH_3_), 4.13 (s, 2H, CH_2_S), 7.35 (t, 1H, C5-H, *J* = 7.2 Hz), 7.47 (d, 2H, C2′-H and C6′-H, *J* = 7.2 Hz), 7.52 (t, 1H, C6-H, *J* = 8.00 Hz), 7.64 (d, 1H, C4-H, *J* = 8.4 Hz), 7.78 (d, 2H, C3′-H and C5′-H, *J* = 8 Hz), 7.89 (d, 1 H, C7-H, *J* = 4 Hz). ^13^C NMR (DMSO-*d*_6_) *δ* ppm: 22.45, 32.82, 79.21, 113.74, 118.16, 122.15, 123.28, 123.58, 124.64, 126.08, 128.49, 130.47, 132.65, 135.22, 140.62, 152.75, 158.62, 159.56, and 172.87. MS *m*/*z* (%): 467 (M^+^, 20.97), 469 (M^+^+2, 13.95). Anal. Calcd. for C_21_H_14_ClN_5_O_2_S_2_: C 53.90, H 3.02, N 14.9; found: C 53.91, H 3.06, N 14.91.

##### Ethyl-2-(2-((2-(benzo[d]thiazol-2-ylamino)-2-oxoethyl)thio)-4-(4-chlorophenyl)-5-cyano-6-oxopyrimidin-1(6H)-yl)acetate (5b)

Yield 68%; melting point: 178–180 °C. IR (*ʋ*_max_/cm^–1^): 3392 (stretching NH), 3061 (aromatic CH), 2922 (aliphatic CH), 2206 (C≡N), 1702, 1688 (C═O), 1533 (C═N), 1459 (C═C). ^1^H NMR (DMSO-*d*_6_) *δ* ppm: 1.17 (t, 3 H, CH_3_, ethyl, *J* = 8.4 Hz), 4.11 (q, 2 H, CH_2_, ethyl, *J* = 6.8 Hz), 4.31 (s, 2 H, CH_2_S), 5.20 (s, 2 H, N-CH_2_), 7.38 (t, 1 H, C5-H, *J* = 6.4 Hz), 7.51 (d, 2 H, C2′-H and C6′-H, *J* = 6.2 Hz), 7.67 (d, 2 H, C2′-H and C6′-H, *J* = 9.6 Hz), 7.80 (d, 1H, C4-H, *J* = 8.4 Hz), 7.84 (t, 1 H, C6-H, J = 7.4 Hz), 7.94 (d, 1 H, C7-H, *J* = 7.6 Hz). ^13^C NMR (DMSO-*d*_6_) *δ* ppm: 23.08, 34.16, 44.38, 63.05, 96.20, 112.70, 114.32, 118.58, 122.33, 126.61, 128.18, 130.92, 131.51, 133.76, 134.18, 140.51, 148.27, 159.04, 162.36, 168.27, 169.93, and 173.46. MS *m*/*z* (%): 539 (M^+^, 10.98), 540 (M^+^+1, 2.66), 541 (M^+^+2, 3.07). Anal. Calcd. for C_24_H_18_ClN_5_O_4_S_2_: C 53.38, H 3.36, N 12.97; found: C 53.36, H 3.40, N 13.02.

##### N-(benzo[d]thiazol-2-yl)-2-((1-benzyl-4-(4-chlorophenyl)-5-cyano-6-oxo-1,6-dihydro pyrimidin-2-yl)thio)acetamide (5c)

Yield 73%; melting point: 201–203 °C. IR (*ʋ*_max_/cm^–1^): 3363 (stretching NHs); 2977 (aromatic CH), 2917 (aliphatic CH), 2213 (C≡N), 1692 (C═O), 1590 (C═N), 1547 (C═C). ^1^H NMR (DMSO-*d*_6_) *δ* ppm: 4.26 (s, 2H, CH_2_S), 5.14 (s, 2H, CH_2_-phenyl), 7.32 (t, 1H, C5-H, *J* = 7.2 Hz), 7.43 (t, 3H, C3″-H, C5″-H and C6-H, *J* = 6.8 Hz), 7.46 (t, 1H, C4″-H, *J* = 5.6 Hz), 7.76 (d, 2H, C2″-H and C6″-H, *J* = 6.2 Hz), 7.79 (d, 2H, C2′-H, C6′-H, *J* = 8.2 Hz), 7.83 (d, 3H, C3′-H, C5′-H, and C4-H, *J* = 8.6 Hz), 7.96 (d, 1H, C7-H, *J* = 8.00 Hz), 12.44 (s, 1H, NH, D_2_O exchangeable). ^13^C NMR (DMSO-*d*_6_) *δ* ppm: 35.53, 56.50, 89.51, 114.90, 120.71, 121.83, 121.95, 126.91, 128.02, 129.07, 129.31, 130.21, 132.08, 132.31, 133.56, 135.07, 151.72, 157.30, 163.03, 166.81, 169.17, 169.63, and 173.83. MS *m*/*z* (%): 543 (M^+^, 34.58), 545 (M^+^+2, 19.79). Anal. Calcd. for C_27_H_18_ClN_5_O_2_S_2_: C 59.61, H 3.33, N 12.87; found: C 59.63, H 3.35, N 12.80.

#### Synthesis of N-(benzo[d]thiazol-2-yl)-2-((4-chloro-6–(4-chlorophenyl)-5-cyanopyrimidin-2-yl)thio)acetamide (6)

A 10 mmol of compound **4** (4.51 g) was dissolved in 30 mL of phosphorus oxychloride and then underwent 3 h reflux. Then, leaving the reaction mixture to cool was done, and was followed by pouring onto cold water with continuous agitation. The formed precipitate was subjected to filtration, and recrystallisation using aqueous ethyl alcohol.

Yield 77%; melting point: 185–187 °C. IR (*ʋ*_max_/cm^–1^): 3435 (stretching NH), 3199 (aromatic CH), 2918 (aliphatic CH), 2217 (C≡N), 1696 (C═O), 1580 (C═N), 1536 (C═C). ^1^H NMR (DMSO-*d*_6_) *δ* ppm: 4.08 (s, 2H, CH_2_S), 7.26 (d, 2H, C2′-H and C6′-H, *J* = 4.0 Hz), 7.32 (t, 1H, C5-H, *J* = 7.0 Hz), 7.46 (t, 1H, C6-H, *J* = 7.8 Hz), 7.71 (d, 2H, C3′-H and C5′-H, *J* = 6.8 Hz), 7.70 (d, 1H, C4-H, *J* = 8.0 Hz), 7.97 (d, 1H, C7-H, *J* = 7.6 Hz), 12.38 (s, 1H, NHCO, D_2_O exchangeable). ^13^C NMR (DMSO-*d*_6_) *δ* ppm: 35.11, 93.76, 116.15, 120.67, 121.79, 128.04, 128.70, 129.09, 132.01, 132.05, 133.62, 135.34, 146.89, 157.39, 161.34, 165.80, 167.09, and 167.55. MS *m*/*z* (%): 470 (M^+^, 60.60), 472 (M^+^+2, 20.43). Anal. Calcd. for C_20_H_11_Cl_2_N_5_OS_2_: C 50.85, H 2.35, N 14.83; found: C 50.87, H 2.40, N 14.91.

#### General method for synthesis of N-(benzo[d]thiazol-2-yl)-2-(4-substituted-5-cyano-6-(phenylamino)pyrimidin-2-yl)thio)acetamide (7a–f)

Few drops of TEA were added to a mixture of 10 mmol of **6** (4.70 g) and 10 mmol of primary or secondary amino derivative in 30 mL absolute ethyl alcohol and then was allowed to stir without heating for 24 h. Further, the reaction mixture underwent 6–12 h reflux. The mixture was leaved to cool and was then concentrated using reduced pressure. The residue was then treated with a plenty of water and the separated precipitate was obtained by filtration. The collected solid was subjected to washing with water, drying, and recrystallisation from methyl alcohol.

##### N-(benzo[d]thiazol-2-yl)-2-((4-(4-chlorophenyl)-5-cyano-6-(phenylamino)pyrimidin-2-yl)thio)acetamide (7a)

Yield 81%; melting point: 222–224 °C. IR (*ʋ*_max_/cm^–1^): 3423 (2 stretching NHs); 3161 (aromatic CH), 2964 (aliphatic CH), 2227 (C≡N), 1672 (C═O), 1604 (C═N), 1552 (C═C). ^1^H NMR (DMSO-*d*_6_) *δ* ppm: 4.22 (s, 2H, CH_2_S), 7.27 (d, 2H, C3′-H and C5′-H, *J* = 8.2 Hz), 7.42 (t 1H, C5-H, *J* = 7.6 Hz), 7.53 (t, 1H, C4″-H, *J* = 7.2 Hz) 7.63 (d, 2H, C2′-H and C6′-H, *J* = 9.4 Hz), 7.70 (d, 3H, C4-H, C2″-H and C6″-H, *J* = 8.6), 7.82 (t, 3H, C6-H, C3″-H and C5″-H, *J* = 8.4 Hz), 7.97 (d, 1H, C7-H, *J* = 6.8 Hz), 10.15 (s, 1H, NH, D_2_O exchangeable), 12.37 (s, 1H, NHCO, D_2_O exchangeable). ^13^C NMR (DMSO-*d*_6_) *δ* ppm: 35.66, 85.09, 113.96, 116.25, 120.75, 122.07, 123.67, 125.77, 126.44, 128.53, 128.96, 130.98, 132.08, 135.03, 136.41, 149.28, 157.07, 159.53, 160.46, 167.14, 168.18, and 173.25. Mass spectrum *m*/*z* (%): 528 (M^+^, 5.72), 529 (M^+^+1, 1.22), 530 (M^+^+2, 2.28). Anal. Calcd. for C_26_H_17_ClN_6_OS_2_: C 59.03, H 3.24, N 15.89; found: C 59.02, H 3.20, N 15.94.

##### N-(benzo[d]thiazol-2-yl)-2-((4-(4-chlorophenyl)-5-cyano-6-((4-methoxyphenyl) amino)pyrimidin-2-yl)thio)acetamide (7b)

Yield 75%; melting point: 236–238 °C. IR (*ʋ*_max_/cm^–1^): 3424, 3321 (2 stretching NHs), 3040 (aromatic CH), 2919 (aliphatic CH), 2213 (C≡N), 1692 (C═O), 1607 (C═N), 1549 (C═C). ^1^H NMR (DMSO-*d*_6_) *δ* ppm: 3.67 (s, 3H, OCH_3_), 4.16 (s, 2H, CH_2_S), 6.81 (d, 2H, C3″-H and C5″-H, *J* = 8.0 Hz), 7.29 (d, 2H, C3′-H and C5′-H, *J* = 8.4 Hz), 7.38 (t, 1H, C5-H, *J* = 6.6 Hz), 7.41 (d, 2H, C2″-H, C6″-H *J* = 6.8 Hz), 7.49 (t, 1H, C6-H, *J* = 7.4 Hz), 7.66 (d, 2H, C2′-H and C6′-H, *J* = 8.4 Hz), 7.77 (d, 1H, C4-H, *J* = 8.4 Hz), 7.81 (d, 1H, C7-H, *J* = 7.6 Hz), 9.72 (s, 1H, NH, D_2_O exchangeable), 12.53 (s, 1H, NHCO, D_2_O exchangeable). ^13^C NMR (DMSO-*d*_6_) *δ* ppm: 35.66, 55.47, 85.09, 113.96, 116.25, 120.75, 122.07, 123.67, 125.77, 126.44, 128.53, 128.96, 130.63, 130.98, 132.08, 135.03, 136.41, 149.28, 157.07, 160.46, 167.14, 168.18, and 173.25. MS *m*/*z* (%): 558 (M^+^, 21.57), 559 (M^+^+1, 5.20), 560 (M^+^+2, 8.93). Anal. Calcd. for C_27_H_19_N_6_O_2_S_2_: C 62.44, H 4.12, N 15.60; found: C 62.45, H 4.16, N 15.66.

##### N-(benzo[d]thiazol-2-yl)-2-((4-(4-chlorophenyl)-6-((4-chlorophenyl)amino)-5-cyano pyrimidin-2-yl)thio)acetamide (7c)

Yield 81%; melting point: 212–214 °C. IR (cm^–1^): 3435, 3256 (2 stretching NHs, stretching), 3172 (aromatic CH), 2913 (aliphatic CH), 2212 (C≡N), 1682 (C═O), 1644 (C═N), 1535 (C═C), 756 (C-Cl).^1^H NMR (DMSO-*d*_6_) *δ* ppm: 4.21 (s, 2H, CH_2_S), 7.29 (t 1H, C5-H, *J* = 10.4 Hz), 7.46 (t 1H, C6-H, *J* = 10.6 Hz), 7.55 (d, 4H, C3″-H and C5″, C3′-H and C5′-H, *J* = 7.2 Hz), 7.81 (d, 4H, C2″-H and C6″-H, C2′-H and C6′-H *J* = 9.2 Hz), 7.85 (d, 1H, C4-H, *J* = 7.6 Hz), 7.99 (d, 1H, C7-H, *J* = 6.4 Hz), 9.91 (s, 1H, NH, D_2_O exchangeable), 12.54 (s, 1H, NHCO, D_2_O exchangeable). ^13^C NMR (DMSO-*d*_6_) *δ* ppm: 35.28, 85.97, 116.03, 121.08, 122.18, 124.06, 125.56, 126.64, 128.75, 128.98, 129.39, 131.01, 131.90, 134.81, 136.58, 136.84, 149.07, 158.27, 160.42, 167.34, 167.59, and 173.13. MS *m*/*z* (%): 562 (M^+^, 7.40), 563 (M^+^+1, 2.84), 564 (M^+^+2, 1.76). Anal. Calcd. for C_26_H_16_Cl_2_N_6_OS_2_: C 55.42, H 2.86, N 14.91; found: C 55.44, H 2.89, N 14.98.

##### 2-((2-((2-(Benzo[d]thiazol-2-ylamino)-2-oxoethyl)thio)-6–(4-chlorophenyl)-5-cyano pyrimidin-4-yl)amino)benzoic acid (7d)

Yield 76%; melting point: 281–282 °C. IR (*ʋ*_max_/cm^–1^): 3415, 3224 (2 stretching NHs), 3160 (aromatic CH), 2955, 2836 (aliphatic CH), 2227 (C≡N), 1721, 1673 (2C═O), 1590 (C═N), 1569 (C═C). ^1^H NMR (DMSO-*d*_6_) *δ* ppm: 4.33 (s, 2H, CH_2_S), 7.18 (t, 1H, C4″-H, *J* = 7.2 Hz), 7.33 (t, 1H, C5-H, *J* = 7.6 Hz), 7.45 (t, 1H, C6-H, *J* = 8.2 Hz), 7.61 (t, 1H, C5″-H, *J* = 8.4 Hz), 7.82 (d, 3H, C4-H, C3′-H and C5′-H, *J* = 7.2 Hz), 7.89 (d, 3H, C3″-H, *J* = 8.4 Hz), 7.98 (d, 2H, C2′-H and C6′-H, *J* = 7.6 Hz), 8.02 (d, 1H, C7-H, *J* = 8.0 Hz), 8.63 (d, 1H, C6″-H, *J* = 8.4 Hz), 11.94 (s, 1H, COOH, D_2_O exchangeable), 12.64 (br, 2H, NH and NHCO, D_2_O exchangeable). ^13^C NMR (DMSO-*d*_6_) *δ* ppm: 35.10, 93.79, 114.96, 116.15, 120.66, 121.76, 128.02, 128.69, 128.97, 129.09, 129.21, 132.00, 133.61, 146.87, 157.40, 157.47, 161.34, 165.77, 167.07, 167.55, 167.63, 168.48, 169.93, 172.55, and 173.88. MS *m*/*z* (%): 572 (M^+^, 94.03), 573 (M^+^+1, 15.72), 574 (M^+^+2, 34.99). Anal. Calcd. for C_27_H_17_ClN_6_O_3_S_2_: C 56.59, H 2.99, N 14.67; found: C 56.57, H 3.03, N 14.75.

##### N-(benzo[d]thiazol-2-yl)-2-((4-(4-chlorophenyl)-5-cyano-6-(pyrrolidin-1-yl) pyrimidin-2-yl)thio)acetamide (7e)

Yield 77%; melting point: 234–236 °C. IR (*ʋ*_max_/cm^–1^): 3391 (stretching NH), 3060 (aromatic CH), 2968, 2925 (aliphatic CH), 2208 (C≡N), 1682 (C═O), 1545 (C═N), 1463 (C═C). ^1^H NMR (DMSO-*d*_6_) *δ* ppm:1.84 (br, 4H, CH_2_-N-CH_2_), 3.72 (br, 4H, CH_2_CH_2_), 4.23 (s, 2H, CH_2_S), 7.32 (t, 1H, C5-H, *J* = 7.2 Hz), 7.48 (d, 3H, C4-H, 3′-H and C5′-H, *J* = 7.6 Hz), 7.75 (t, 1H, C6-H, *J* = 8 Hz), 7.88 (d, 2H, C2′-H and C6′-H, *J* = 5.2 Hz), 7.97 (d, 1H, C7-H, *J* = 7.6 Hz), 12.60 (br, 1H, NH, D_2_O exchangeable). ^13^C NMR (DMSO-*d*_6_) *δ* ppm: 22.01, 35.66, 49.05, 83.63, 118.45, 121.20, 122.18, 124.12, 126.69, 128.76, 130.22, 131.53, 135.37, 136.34, 148.90, 158.41, 168.12, 168.91, 171.96, and 172.94. MS *m*/*z* (%): 506 (M^+^, 12.18), 507 (M^+^+1, 1.65), 508 (M^+^+2, 3.65). Anal. Calcd. for C_24_H_19_ClN_6_OS_2_: C 56.85, H 3.78, N 16.57; found: C 56.87, H 3.81, N 16.65.

##### N-(benzo[d]thiazol-2-yl)-2-((4-(4-chlorophenyl)-5-cyano-6-thiomorpholino pyrimidin-2-yl) thio)acetamide (7f)

The yield was of 84%; melting point: 187–189 °C. IR (*ʋ*_max_/cm^–1^): 3222 (stretching NH), 3155, 3050 (aromatic CH), 2925, 2860 (aliphatic CH), 2227 (C≡N), 1695 (C═O), 1610 (C═N), 1537 (C═C). ^1^H NMR (DMSO-*d*_6_) *δ* ppm: 2.68 (br, 4H, CH_2_-N-CH_2_ thiomorpholine), 4.14 (br, 4H, CH_2_-S-CH_2_, thiomorpholine), 4.27 (s, 2H, CH_2_S), 7.34 (br, 1H, C5-H), 7.47 (br, 1H, C6-H), 7.82 (br, 3H, C4-H, C3′-H and C5′-H), 7.99 (br, 3H, C7-H, C2′-H and C6′-H), 12.55 (br, 1H, NHCO, D_2_O exchangeable). ^13^C NMR (DMSO-*d*_6_) *δ* ppm: 38.25, 49.07, 61.87, 92.98, 112.69, 116.59, 123.46, 125.40, 128.81, 129.12, 132.05, 134.49, 134.54, 135.68, 166.66, 167.27, 167.30, 167.47, and 176.73. MS *m*/*z* (%): 538 (M^+^, 13.28), 539 (M^+^+1, 1.18), 539 (M^+^+2, 2.98). Anal. Calcd. for C_24_H_19_ClN_6_OS_3_: C 53.47, H 3.55, N 15.59; found: C 53.46, H 3.51, N 15.65.

#### Synthesis of 2-((5-cyano-4-hydrazinyl-6-phenylpyrimidin-2-yl) thio)-N-(6-methylbenzo[d]thiazol-2-yl)acetamide (8)

Equimolar quantities (10 mmol) of hydrazine hydrate (98%) (0.5 g) and **6** (4.70 g) were dissolved in 30 mL of absolute ethanol. After 6 h reflux, the separated material was then cooled by filtration and watery washing. The obtained solid was air dried and underwent recrystallisation using acetic acid and methyl alcohol. Yield 71%; melting point: 235–237 °C. IR (*ʋ*_max_/cm^–1^): 3460 (2 stretching NH), 3291, 3206 (NH_2_), 3056 (aromatic CH), 2919 (aliphatic CH), 2215 (C≡N), 1703 (C═O), 1585, (NH_2_ bend, C═N), 1461 (C═C). ^1^H NMR (DMSO-*d*_6_) *δ* ppm: 4.29 (s, 2H, CH_2_S), 5.04 (s, 2H, NH_2_, D_2_O exchangeable), 7.27 (d, 2H, C3′-H and C5′-H, *J* = 8.4 Hz), 7.41 (t, 1H, C5-H, *J* = 7.6 Hz), 7.51 (t, 1H, C6-H, *J* = 7.2 Hz), 7.66 (d, 3H, C4-H, C2′-H and C6′-H, *J* = 8.4 Hz), 7.75 (d, 1H, 4-H, *J* = 7.6 Hz), 7.80 (d, 1H, C7-H, *J* = 7.6 Hz), 12.47 (s, 2H, NH and NHCO, D_2_O exchangeable). ^13^C NMR (DMSO-*d*_6_) *δ* ppm: 35.20, 100.40, 120.65, 121.76, 128.01, 129.11, 129.43, 131.02, 132.04, 133.61, 136.70, 146.95, 148.27, 155.75, 157.56, 161.73, 166.98, and 168.26. MS *m*/*z* (%): 467 (M^+^, 23.37), 469 (M^+^+2, 9.62), Anal. Calcd. for C_20_H_14_ClN_7_OS_2_: C 51.33, H 3.02, N 20.95; found: C 51.35, H 3.06, N 21.01.

#### Synthesis of N-(benzo[d]thiazol-2-yl)-2-((4-(4-chlorophenyl)-5-cyano-6-thioxo-1,6-dihydropyrimidin-2-yl)thio)acetamide (9)

The reflux of equimolar quantities (10 mmol) of **6** (4.70 g) as well as thiourea (0.76 g) was done in 20 mL absolute ethyl alcohol for 6 h. The deposit was yielded by filtration off. The collected deposit was then leaved to dry and recrystallised using dioxane. Yield 76%; melting point: 280–282 °C. IR (*ʋ*_max_/cm^–1^): 3441, 3203 (2 stretching NHs), 3033 (aromatic CH), 2920 (aliphatic CH), 2218 (C≡N), 1692 (C═O), 1540 (C═N), 1472 (C═C), 1278 (C═S). ^1^H NMR (DMSO-*d*_6_) *δ* ppm: 4.38 (s, 2H, CH_2_S), 7.27 (d, 2H, C3′-H and C5′-H, *J* = 7.6 Hz), 7.36 (t, 1H, C5-H, *J* = 7.8 Hz), 7.54 (t, 1H, C6-H, *J* = 7.4 Hz), 7.66 (d, 2H, C2′-H and C6′-H, *J* = 8.4 Hz), 7.83 (d, 1H, C4-H, *J* = 8.0 Hz), 7.88 (d, 1H, C7-H, *J* = 8.0 Hz), 12.59 (s, 2H, 2NH, D_2_O exchangeable). ^13^C NMR (DMSO-*d*_6_) *δ* ppm: 35.06, 93.80, 116.13, 120.71, 121.81, 128.06, 128.71, 129.01, 129.08, 129.22, 132.05, 133.65, 146.96, 161.38, 165.80, 167.06, 167.61, 173.88. MS *m*/*z* (%): 468 (M^+^, 11.33), 469 (M^+^+1, 6.02), 470 (M^+^+2, 1.43). Anal. Calcd. for C_20_H_12Cl_N_5_OS_3_: C 51.11, H 2.57, N 14.90; found: C 51.13, H 2.61, N 14.87.

#### Synthesis of 2-((2-((2-(benzo[d]thiazol-2-ylamino)-2-oxoethyl)thio)-6-(4-chlorophenyl)-5-cyanopyrimidin-4-yl)amino)acetic acid (10)

The reflux of equimolar amounts 10 mmol of both compound **6** (4.70 g) in addition to glycine (0.75 g) was performed in 30 mL n-butyl alcohol for 3 h. The formed solid was separated by filtration and was air dried. Yield 80%; melting point: 270–272 °C. IR (*ʋ*_max_/cm^−1^): 3431, 3127 (2 stretching NH), 3015 (aromatic CH), 2833 (aliphatic CH), 2223 (C≡N), 1715, 1669 (2C═O), 1598 (C═N), 1557 (C═C).^1^H NMR (DMSO-*d*_6_) *δ* ppm: 4.12 (s, 2H, CH_2_-NH), 4.24 (s, 2H, CH_2_S), 7.38 (br, 1H, C5-H), 7.48 (br, 1H, C6-H), 7.82 (br, 3H, C4-H, C3′-H and 5′-H), 7.99 (br, 3H, C7-H, C2′-H and C6′-H), 8.38 (br, 1H, NH-CH_2_, D_2_O exchangeable), 12.69 (br, 2H, NHCO and COOH, D_2_O exchangeable). ^13^C NMR (DMSO-*d*_6_) *δ* ppm: 35.39, 49.06, 84.87, 118.30, 120.80, 121.66, 128.00, 128.89, 129.68, 131.08, 131.98, 133.52, 136.38, 146.97, 157.49, 167.11, 166.82, 167.68, 170.67, and 172.00. MS *m*/*z* (%): 510 (M^+^, 27.28), 512 (M^+^+2, 18.43). Anal. Calcd. for C_22_H_15_ClN_6_O_3_S_2_: C 51.71, H 2.96 N, 16.45; found: C 51.73, H 3.01, N 16.53.

#### Synthesis of N-(benzo[d]thiazol-2-yl)-2-((7-(4-chlorophenyl)-8-cyano-3-oxo-2,3-dihydroimidazo[1,2-c]pyrimidin-5-yl)thio)acetamide (11)

Compound 1**0** (5.10 g, 10 mmol) and 5 mL of acetic anhydride were heated under reflux for 2 h. After cooling, the precipitate was separated with filtration off and was then subjected to airily drying. Finally, compound **11** was formed through recrystallisation from acetic acid. Yield 68%; melting point: 267–269 °C. IR (*ʋ*_max_/cm^−1^): 3294 (stretching NH), 3053 (aromatic CH), 2968, 2916 (aliphatic CH), 2211 (C≡N), 1693 (2C═O), 1605 (C═N), 1563 (C═C).^1^H NMR (DMSO-*d*_6_) *δ* ppm: 4.04 (s, 2H, CH_2_, imidazole), 4.39 (s, 2H, CH_2_S), 7.29 (d, 2H, C3′-H and C5′-H, *J* = 8.0 Hz), 7.38 (t, 1H, C5-H, *J* = 7.6 Hz), 7.54 (t, 1H, C6-H, *J* = 7.2 Hz), 7.67 (d, 2H, C2′-H and C6′-H, *J* = 8.0 Hz), 7.83 (d, 1H, C4-H, *J* = 8.4 Hz), 7.89 (d, 1H, C7-H, *J* = 8.0 Hz), 12.66 (s, 1H, NHCO, D_2_O exchangeable). ^13^C NMR (DMSO-*d*_6_) *δ* ppm: 35.53, 56.50, 89.51, 114.90, 120.71, 121.83, 121.95, 126.91, 128.02, 129.07, 129.31, 130.21, 132.31, 133.56, 135.07, 151.72, 157.45, 167.01, 169.17, 169.63, and 173.83. MS *m*/*z* (%): 492 (M^+^, 62.79), 493 (M^+^+1, 13.85), 494 (M^+^+2, 18.47). Anal. Calcd. for C_22_H_13_ClN_6_O_2_S_2_: C 53.60, H 2.66, N 17.05; found: C 53.62, H 2.62, N 17.12.

#### Synthesis of N-(benzo[d]thiazol-2-yl)-2-((3-(4-chlorophenyl)-4-cyano-10-oxo-10H-pyrimido[6,1-b]quinazolin-1-yl)thio)acetamide (12)

Compound **12** was formed by the fusion of 10 mmol of both the anthranilic acid (1.37 g) and the compound **6** (4.70 g) in a bath of oil at 190 °C for 2 h. After cooling, the mixture was poured on ice and the solid was separated by filtration. The separated solid was subjected to airily drying and recrystallisation using benzene. Yield 69%; melting point: 190–192 °C. IR (*ʋ*_max_/cm^−1^): 3200 (stretching NH), 3065 (aromatic CH), 2971, 2916 (aliphatic CH), 2207 (C≡N), 1689 (2C═O), 1603 (C═N), 1540 (C═C). ^1^H NMR (DMSO-*d*_6_) *δ* ppm: 4.30 (s, 2H, CH_2_S), 7.32 (t, 1H, C5-H, *J* = 7.4 Hz), 7.38 (t, 1H, C7″-H, pyrimido quinazoline, *J* = 8.4 Hz), 7.43 (d, 1H, C9″-H (pyrimido quinazoline), *J* = 8.4 Hz), 7.49 (t, 2H, C6-H and C8″-H, *J* = 7.6 Hz), 7.64 (d, 1H, C6″-H (pyrimido quinazoline), *J* = 7.6 Hz), 7.71 (d, 2H, C3′-H and C5′-H, *J* = 7.6 Hz), 7.81 (d, 2H, C2′-H and C6′-H, *J* = 8.4 Hz), 7.84 (d, 1H, C4-H, *J* = 8.4 Hz) 7.98 (d, 1H, C7-H, *J* = 7.6 Hz), 12.69 (s, 1H, NHCO, D_2_O exchangeable). ^13^C NMR (DMSO-*d*_6_) *δ* ppm: 35.10, 93.79, 120.66, 121.76, 128.02, 128.69, 128.97, 129.09, 129.21, 132.00, 133.61, 146.87, 157.40, 157.47, 161.34, 165.77, 167.07, 167.29, 167.55, 167.63, 168.48, 169.93, 172.55, and 173.88. MS *m*/*z* (%): 554 (M^+^, 32.18), 556 (M^+^+2, 12.19). Anal. Calcd. for C_27_H_15_ClN_6_O_2_S_2_: C 58.43, H 2.72, N 15.14; found: C 58.45, H 2.77, N 15.20.

#### Synthesis of N-(benzo[d]thiazol-2-yl)-2-((7-(4-chlorophenyl)-8-cyanotetrazolo[1,5-c]pyrimidin-5-yl)thio)acetamide (13)

A 50 mmol of NaN_3_ (3.25 g) was added to a solution of 10 mmol of **6** (4.70 g) in 30 mL glacial CH_3_COOH and was then allowed for 3 h reflux. After cooling and filtration, the separated solid was dried and the acetic acid was used for recrystallisation to yield **13**. Yield 70%; melting point: 259–261 °C. IR (*ʋ*_max_/cm^−1^): 3296 (stretching NH), 3070 (aromatic CH), 2918 (aliphatic CH), 2211 (C≡N), 1694 (C═O), 1603 (C═N), 1536 (C═C). ^1^H NMR (DMSO-*d*_6_) *δ* ppm: 4.38 (s, 2H, CH_2_S), 7.31 (t, 1H, C5-H, *J* = 7.4 Hz), 7.44 (t, 1H, C6-H, *J* = 8.4 Hz), 7.77 (d, 2H, C3′-H and C5′-H, *J* = 8.0 Hz), 7.88 (d, 3H, C4-H, C2′-H and C6′-H, *J* = 8.4 Hz), 7.97 (d, 1H, C7-H, *J* = 7.6 Hz), 12.62 (s, 1H, NHCO, D_2_O exchangeable). ^13^C NMR (DMSO-*d*_6_) *δ* ppm: 36.02, 93.81, 111.87, 120.73, 121.83, 128.07, 128.85, 129.09, 129.49, 129.82, 132.03, 143.54, 146.79, 151.88, 157.88, 167.11, 170.97, and 172.63. MS *m*/*z* (%): 478 (M^+^, 31.04), 480 (M^+^+2, 12.24). Anal. Calcd. for C_20_H_11_ClN_8_OS_2_: C 50.16, H 2.31, N 23.40; found: C 50.14, H 3.35, N 23.48.

#### Synthesis of N-(benzo[d]thiazol-2-yl)-2-((4-(4-chlorophenyl)-5-cyano-6-(3,5-dimethyl-1H-pyrazol-1-yl)pyrimidin-2-yl)thio)acetamide (14)

The reflux of a solution of equimolar amounts (10 mmol) of both compound **8** (4.67 g) and acetylacetone (1.0 g, 1.02 mL) was done in glacial CH_3_COOH for 6 h. After cooling and filtration, the aqueous ethanol was used for recrystallisation of compound **14**. Yield 76%; melting point: 223–225 °C. IR (*ʋ*_max_/cm^−1^): 3367 (stretching NH), 3197 (aromatic CH), 2919 (aliphatic CH), 2210 (C≡N), 1697 (C═O), 1556 (C═N), 1465 (C═C). ^1^H NMR (DMSO-*d*_6_) *δ* ppm: 2.52 (s, 3H, CH_3_, pyrazole), 2.63 (s, 3H, CH_3_, pyrazole), 4.42 (s, 2H, CH_2_S), 7.32 (t, 1H, C5-H, *J* = 7.2 Hz), 7.47 (t, 1H, C6-H, *J* = 7.6 Hz), 7.53 (s, 1H, CH, pyrazole), 7.55 (d, 2H, C3′-H and C5′-H, *J* = 8.2 Hz), 7.79 (d, 1H, C4-H, *J* = 8.2 Hz), 7.96 (d, 1H, C7-H, *J* = 6.0 Hz), 8.51 (d, 2H, C2′-H and C6′-H, *J* = 8.4 Hz), 12.56 (s, 1H, NHCO, D_2_O exchangeable). ^13^C NMR (DMSO-*d*_6_) *δ* ppm: 21.45, 26.86, 35.33, 86.57, 116.13, 120.77, 122.61, 128.92, 129.23, 132.05, 133.17, 133.51, 135.98, 142.36, 147.15, 157.30, 160.45, 167.47, 168.69, and 173.19. MS *m*/*z* (%): 531 (M^+^, 23.35), 532 (M^+^+1, 16.81), 533 (M^+^+2, 10.47). Anal. Calcd. for C_25_H_18_ClN_7_OS_2_: C 56.44, H 3.41, N 18.43; found: C 56.45, H 3.45, N 18.38.

#### Synthesis of N-(benzo[d]thiazol-2-yl)-2-((4-(4-chlorophenyl)-5-cyano-6-(3-methyl-5-oxo-4,5-dihydro-1H-pyrazol-1-yl)pyrimidin-2-yl)thio) acetamide (15)

A 10 mmol of ethylacetoacetate (1.30 g, 1.27 mL) was added to a 10 mmol of compound **8** (4.67 g) and in a solution of sodium ethoxide that was formed by mixing 10 mmol metallic Na (0.23 g) with 30 mL absolute ethyl alcohol. After 4 h reflux, the mixture was leaved to cool and was added to ice water. The formed precipitate upon neutralisation with acetic acid was separated with filtration off. The mixture of chloroform and methyl alcohol was used for recrystallisation of compound **15**. Yield 64%; melting point: 235–236 °C. IR (*ʋ*_max_/cm^−1^): 3198 (stretching NH), 3048 (aromatic CH), 2954, 2916 (aliphatic CH), 2226 (C≡N), 1696 (C═O), 1607 (C═N), 1581 (C═C). ^1^H NMR (DMSO-*d*_6_) *δ* ppm: 2.41 (s, 3H, CH_3_, pyrazolone), 4.04 (s, 2H, CH_2_, pyrazolone), 4.39 (s, 2H, CH_2_S), 7.27 (d, 2H, C3′-H and C5′-H, *J* = 8.4 Hz), 7.38 (t, 1H, C5-H, *J* = 6.8 Hz), 7.56 (t, 1H, C6-H, *J* = 7.0 Hz), 7.67 (d, 2H, C2′-H and C6′-H, *J* = 8.2 Hz), 7.84 (d, 1H, C4-H, *J* = 6.4 Hz), 7.89 (d, 1H, C7-H, *J* = 7.6 Hz), 12.56 (s, 1H, NHCO, D_2_O exchangeable). ^13^C NMR (DMSO-*d*_6_) *δ* ppm: 21.46, 25.95, 35.53, 89.51, 114.90, 120.71, 121.83, 121.95, 126.91, 128.02, 129.07, 129.31, 130.21, 132.31, 133.56, 135.07, 151.72, 167.01, 169.17, 169.63, 173.83. MS *m*/*z* (%): 533 (M^+^, 24.70), 535 (M^+^+2, 10.15). Anal. Calcd. for C_24_H_16_ClN_7_O_2_S_2_: C 53.98, H 3.02, N 18.36; found: C 54.00, H 3.07, N 18.43.

### Biological evaluation

#### Cultures of mycobacteria

The first line drug sensitive *M. tuberculosis* ATCC 25177/H37Ra and the first line drug resistant *M. tuberculosis* ATCC 35822 were obtained from ATCC (Manassas, VA). The first and second line drug resistant *M. tuberculosis* RCMB 2674, as well as the MDR clinically isolated strains of *M. tuberculosis* were obtained from the RCMB. Dubos medium treated with 50 µmol of NaNO_3_ was used as a medium for the growth of all used *mycobacteria* strains. The cultures were grown to exponential phase optical absorbance, where the absorbance was measured at 595 nm in an aerobic environment at 37 °C. The sonication of TB strains was allowed for 2 min in a watery bath sonicator (Ultrasonic, Freeport, IL), that as the mycobacteria prefer to grow in aggregated clusters.

#### Minimum inhibitory concentration assay

The MABA assay was used for the determination of MICs of all tested hybrids **4**, **5a**–**c**, **6**, **7a**–**f**, and **8**–**15** against three strains of *M. tuberculosis*^50^^,^^51^. INH was used as reference control. The final testing concentrations used for stock solutions of tested compounds were in the range of 1000–0.003 µg/mL. *M. tuberculosis* was allowed to grow to late exponential phase in Broth of Difco Middlebrook 7H9 enriched with 0.2% vol/vol of glycerol, 0.05% Tween 80, and 10% vol/vol of albumin, dextrose, catalase (7 H9–ADC–TG). Preparation of twofold serial dilutions of tested hybrids was done in 100 µL of 7H9–ADC–TG in 96-well clear-bottom micro plates. The ending evaluating volume of 200 µL of *M. tuberculosis* was yielded by addition of 100 µL having 2 × 10^5^ CFU. Then, the incubation of plates was done at 37 °C for 16–24 h. On the seventh day of the incubation, all wells were supplemented with 20 µL of alamar blue and 12.5 µL of 20% Tween 80. After incubation at 37 °C, the reading of fluorescence was taken at values of 530 and 590 nm. The lowest concentration producing a decrease in the 100% fluorescence related to the average of controls of the replicate bacterium only was used for the definition of the MIC^33^.

### Molecular docking

In the present work, molecular operating environment (MOE, 2019.0102) software^52^ was operated for doing the molecular docking, energy minimised structures were gained by applying MMFF94x force field until the reaching of RMSD gradient of 0.1 kcal·mol^–1^ Å^–1^. The X-ray crystallographic structures of DprE1 and TMPKtm enzymes were downloaded from protein data bank (PDB) with the following ID: 4KW5 and 1W2H. Each protein file’s co-crystallised ligand was used to identify the essential binding features to the enzymes using triangle matcher as a placement method and London dG as a scoring algorithm^52^.

## Supplementary Material

Supplemental Material
