# Supplementary material for: Discovery of pyrimidine-tethered benzothiazole derivatives as novel anti-tubercular agents towards multi- and extensively drug resistant Mycobacterium tuberculosis
Source: J Enzyme Inhib Med Chem. 2023 Aug 30;38(1):2250575. doi: 10.1080/14756366.2023.2250575 (PMC10472891; doi:10.1080/14756366.2023.2250575)
Supplement: Supplemental Material [file IENZ_A_2250575_SM4290.pdf]

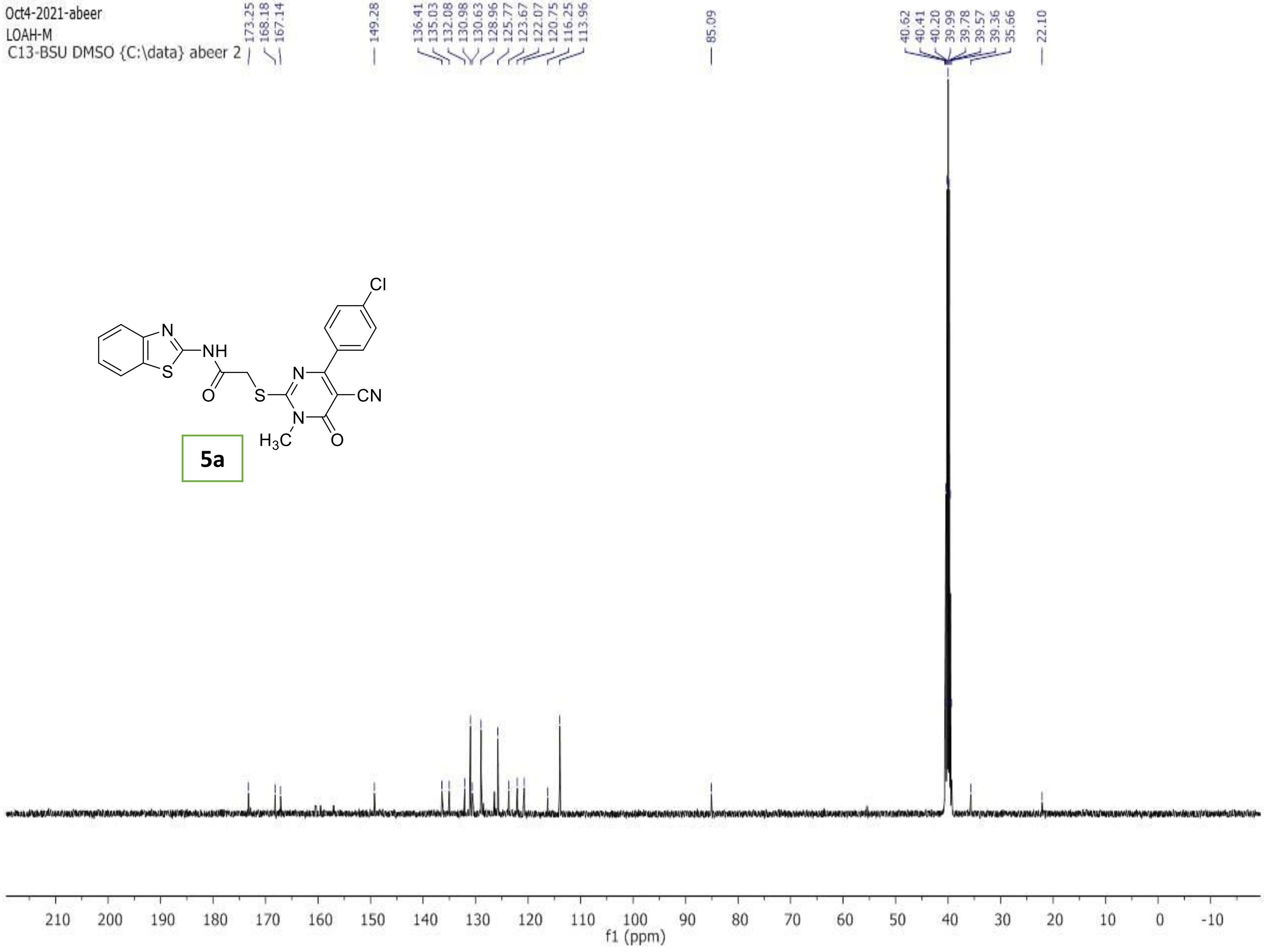

Oct4-2021-abeer
LOAH-M
C13-BSU DMSO {C:\data} abeer 2
173.25
168.18
167.14
149.28
136.41
135.03
132.08
130.98
130.63
128.96
125.77
123.67
122.07
120.75
116.25
113.96
85.09
40.62
40.41
40.20
39.99
39.78
39.57
39.36
35.66
22.10
Cl
N
NH
S
O
S
N
CN
N
H3C
O
5a
210 200 190 180 170 160 150 140 130 120 110 100 90 80 70 60 50 40 30 20 10 0 -10
f1 (ppm)

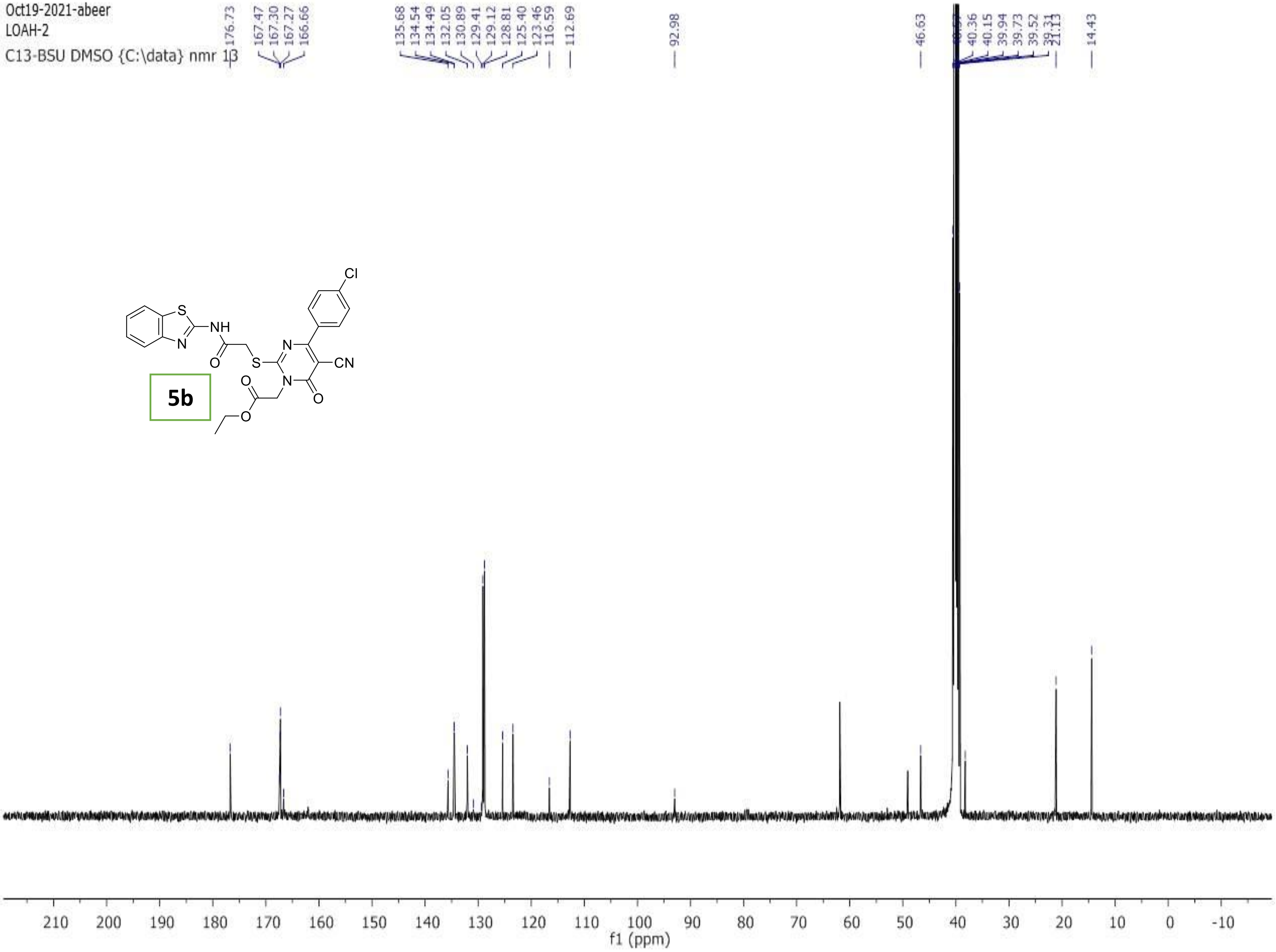

Oct19-2021-abeer
LOAH-2
C13-BSU DMSO {C:\data} nmr 13
176.73
167.47
167.30
167.27
166.66
135.68
134.54
134.49
132.05
130.89
129.41
129.12
128.81
125.40
123.46
116.59
112.69
92.98
46.63
40.36
40.15
39.94
39.73
39.52
39.31
21.13
14.43
Cl
S
NH
N
O
S
N
CN
N
O
O
O
5b
210 200 190 180 170 160 150 140 130 120 110 100 90 80 70 60 50 40 30 20 10 0 -10
f1 (ppm)

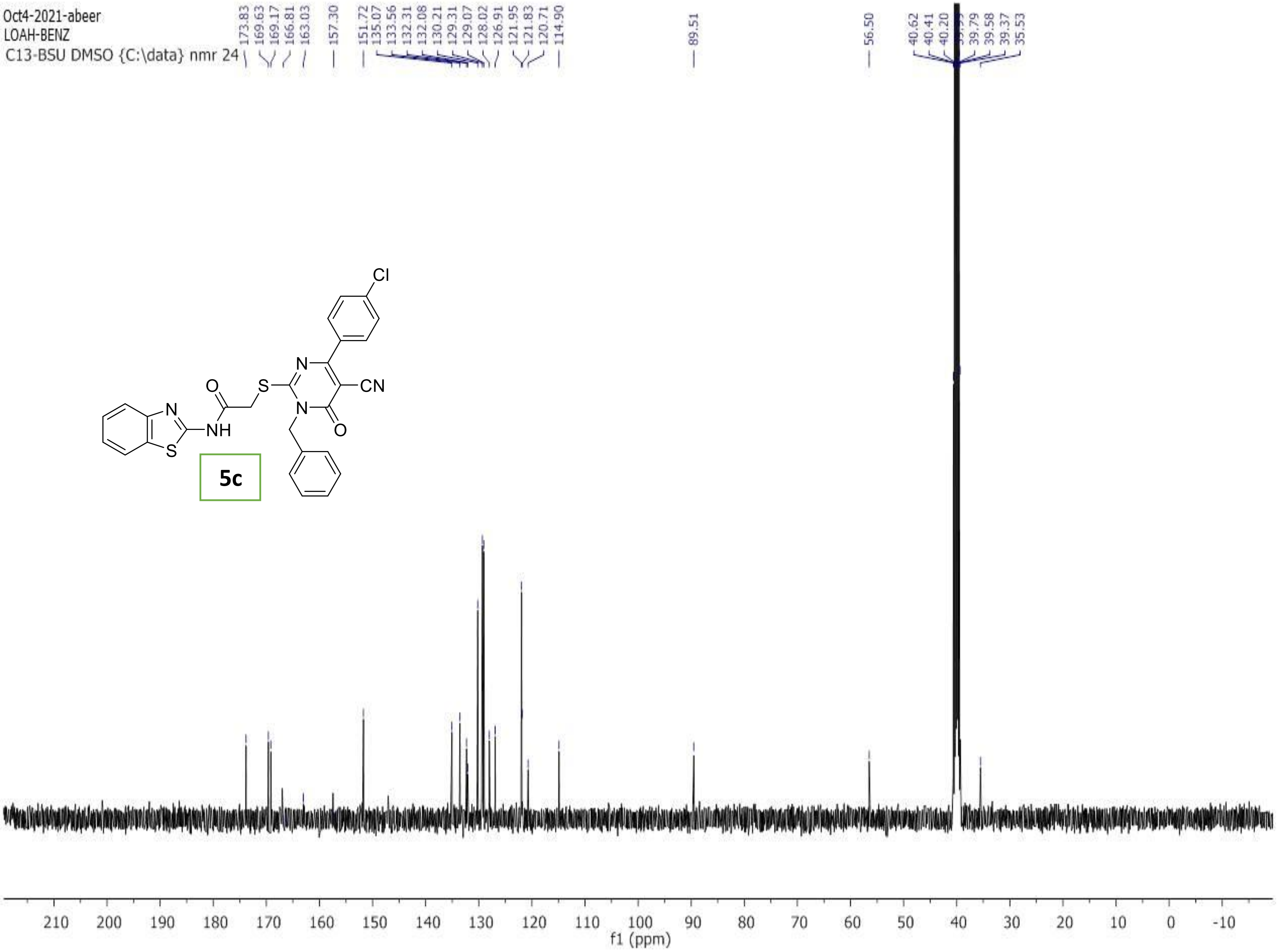

Oct4-2021-abeer
LOAH-BENZ
C13-BSU DMSO {C:\data} nmr 24
173.83
169.63
169.17
166.81
163.03
157.30
151.72
135.07
133.56
132.31
132.08
130.21
129.31
129.07
128.02
126.91
121.95
121.83
120.71
114.90
89.51
56.50
40.62
40.41
40.20
39.79
39.58
39.37
35.53
Cl
N
S
O
CN
N
O
N
NH
S
5c
210 200 190 180 170 160 150 140 130 120 110 100 90 80 70 60 50 40 30 20 10 0 -10
f1 (ppm)

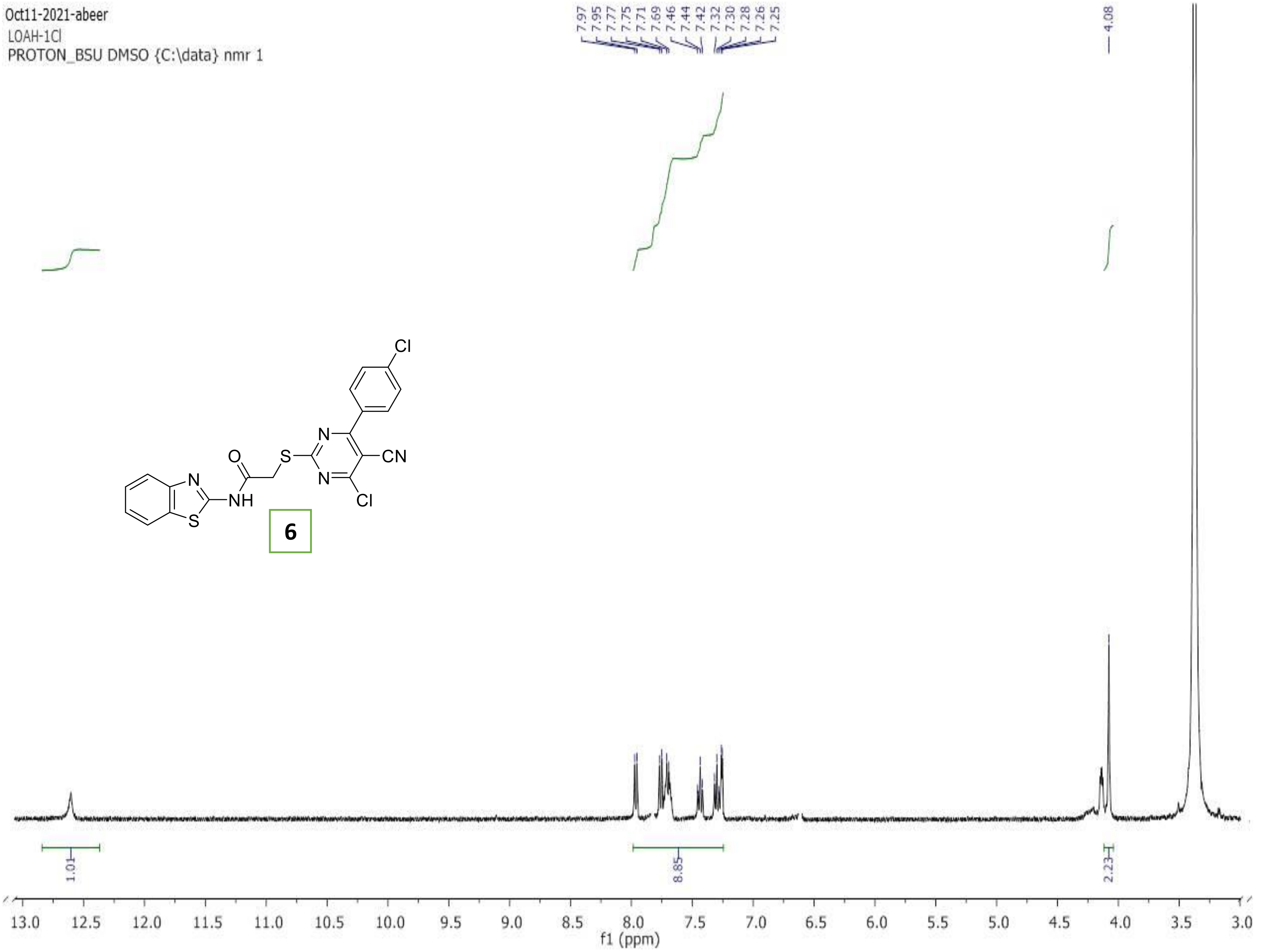

Oct11-2021-abeer
LOAH-1Cl
PROTON_BSU DMSO {C:\data} nmr 1
7.97 7.95 7.77 7.75 7.71 7.69 7.46 7.44 7.42 7.32 7.30 7.28 7.26 7.25
4.08
Cl
N
O
S
CN
N
NH
N
Cl
S
6
1.01
8.85
2.23
13.0 12.5 12.0 11.5 11.0 10.5 10.0 9.5 9.0 8.5 8.0 7.5 7.0 6.5 6.0 5.5 5.0 4.5 4.0 3.5 3.0
f1 (ppm)

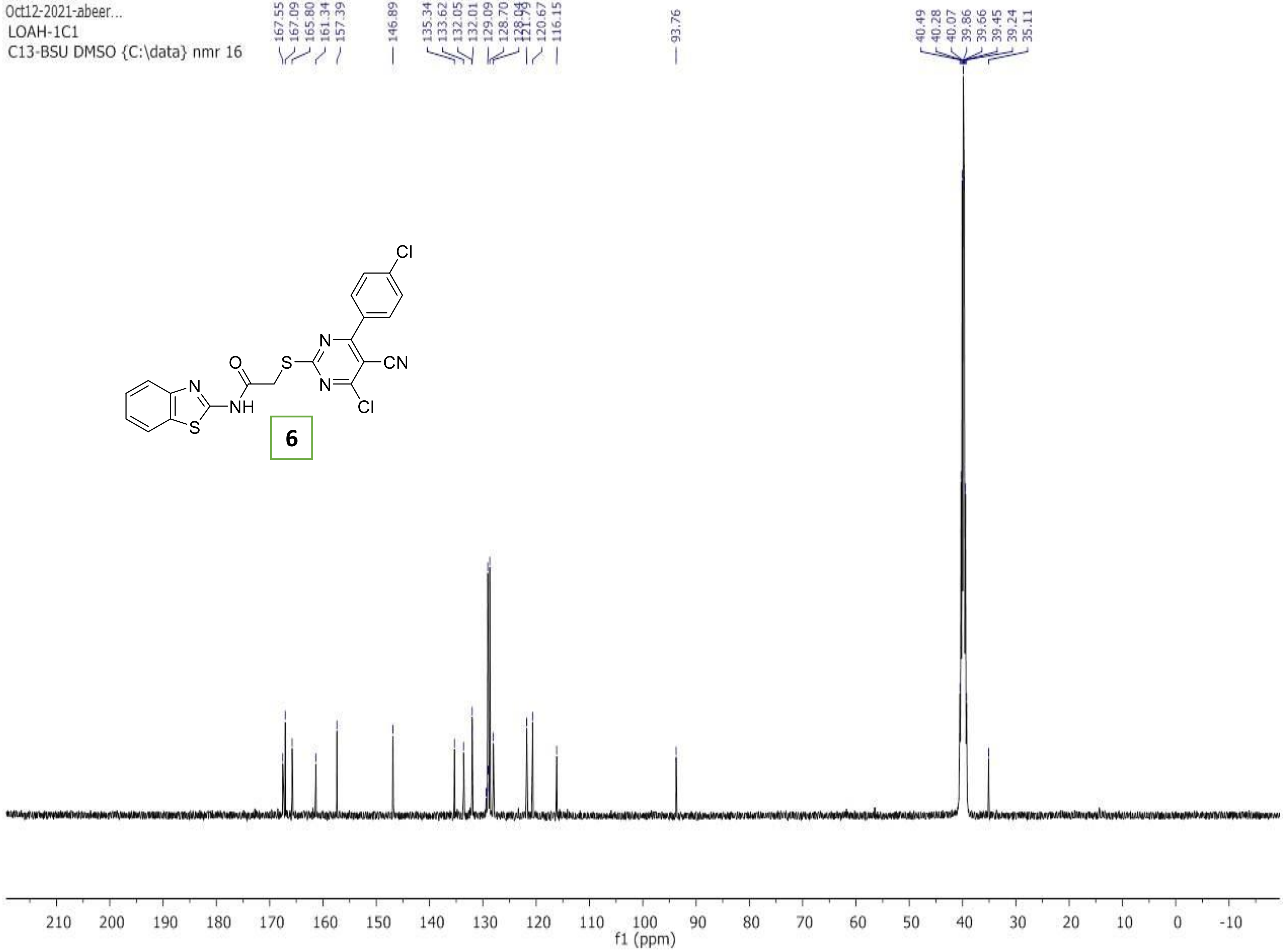
Oct12-2021-abeer...
LOAH-1C1
C13-BSU DMSO {C:\data} nmr 16
167.55
167.09
165.80
161.34
157.39
146.89
135.34
133.62
132.05
132.01
129.09
128.70
128.04
121.79
120.67
116.15
93.76
40.49
40.28
40.07
39.86
39.66
39.45
39.24
35.11
Cl
N
O
S
CN
N
NH
S
Cl
6
210 200 190 180 170 160 150 140 130 120 110 100 90 80 70 60 50 40 30 20 10 0 -10
f1 (ppm)

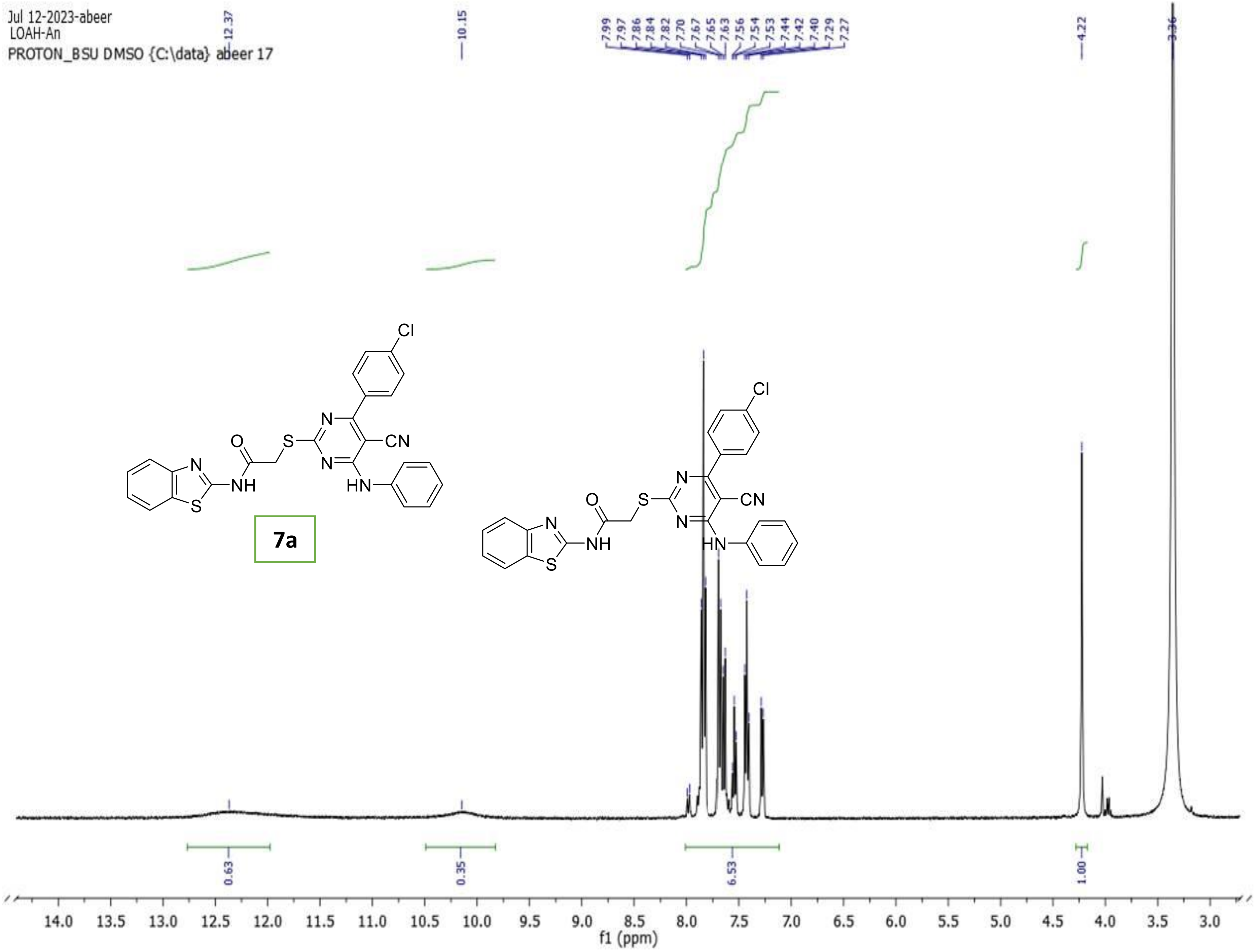
Jul 12-2023-abeer
LOAH-An
PROTON_BSU DMSO {C:\data} abeer 17
12.37
10.15
7.99 7.97 7.86 7.84 7.82 7.70 7.67 7.65 7.63 7.56 7.54 7.53 7.44 7.42 7.40 7.29 7.27
4.22
3.36
7a
0.63
0.35
6.53
1.00
14.0 13.5 13.0 12.5 12.0 11.5 11.0 10.5 10.0 9.5 9.0 8.5 8.0 7.5 7.0 6.5 6.0 5.5 5.0 4.5 4.0 3.5 3.0
f1 (ppm)

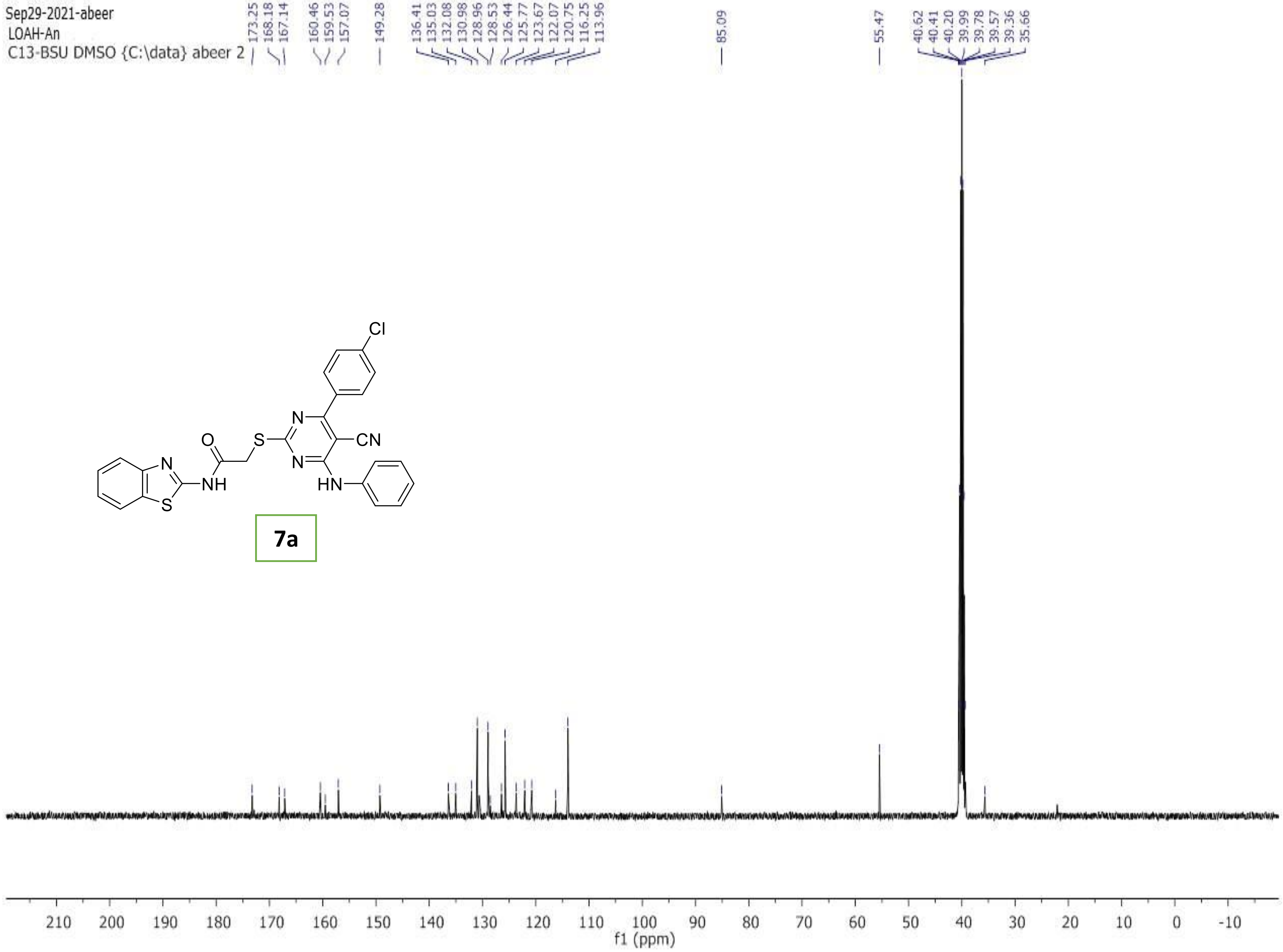
Sep29-2021-abeer
LOAH-An
C13-BSU DMSO {C:\data} abeer 2
173.25
168.18
167.14
160.46
159.53
157.07
149.28
136.41
135.03
132.08
130.98
128.96
128.53
126.44
125.77
123.67
122.07
120.75
116.25
113.96
85.09
55.47
40.62
40.41
40.20
39.99
39.78
39.57
39.36
35.66
Cl
N
S
O
CN
N
NH
HN
S
7a
210 200 190 180 170 160 150 140 130 120 110 100 90 80 70 60 50 40 30 20 10 0 -10
f1 (ppm)

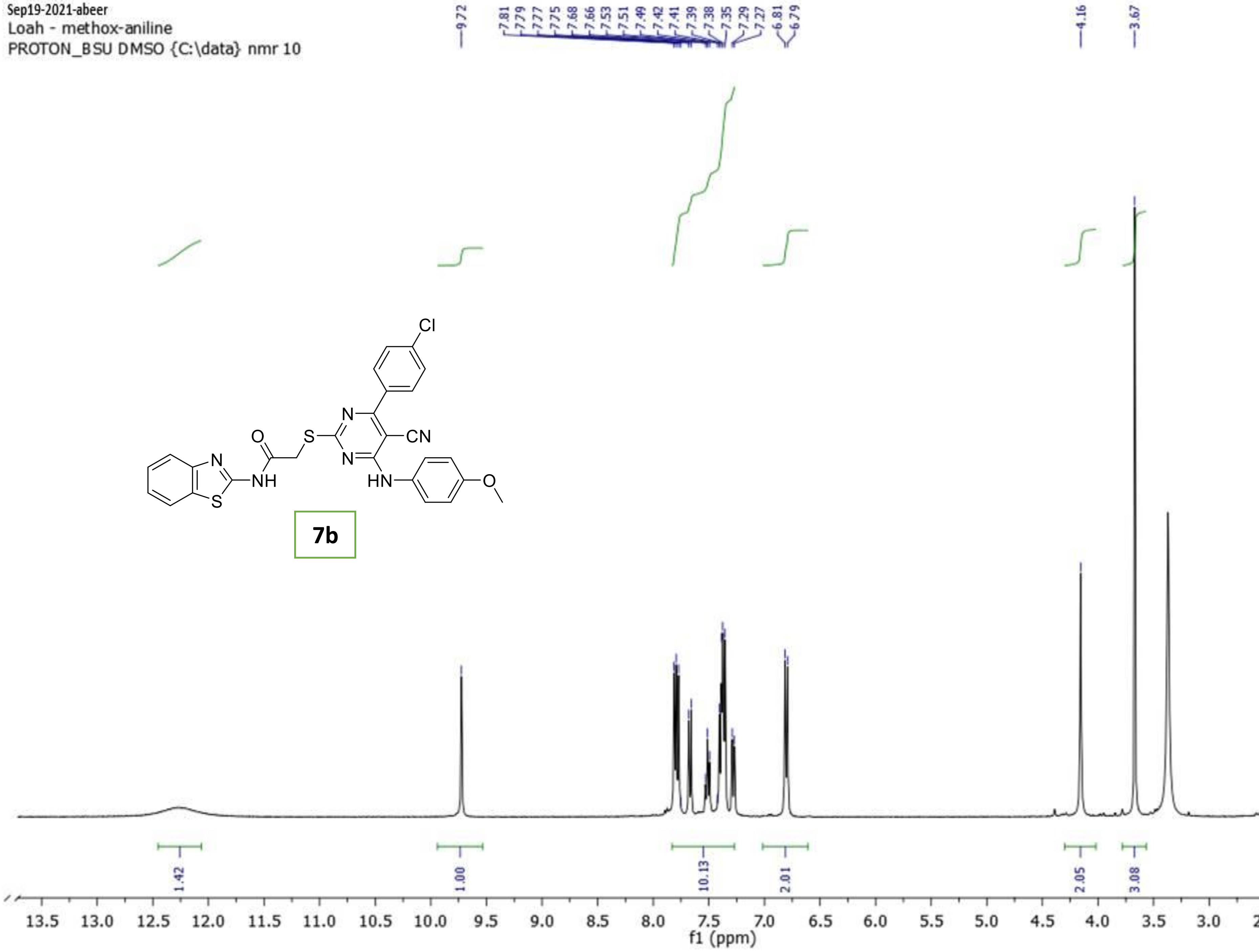
Sep19-2021-abeer
Loah - methox-aniline
PROTON_BSU DMSO {C:\data} nmr 10
9.72
7.81 7.79 7.77 7.75 7.68 7.66 7.53 7.51 7.49 7.42 7.41 7.39 7.38 7.35 7.29 7.27 6.81 6.79
4.16
3.67
Cl
N
S
O
CN
N
N
NH
HN
O
S
7b
1.42
1.00
10.13
2.01
2.05
3.08
13.5 13.0 12.5 12.0 11.5 11.0 10.5 10.0 9.5 9.0 8.5 8.0 7.5 7.0 6.5 6.0 5.5 5.0 4.5 4.0 3.5 3.0
f1 (ppm)

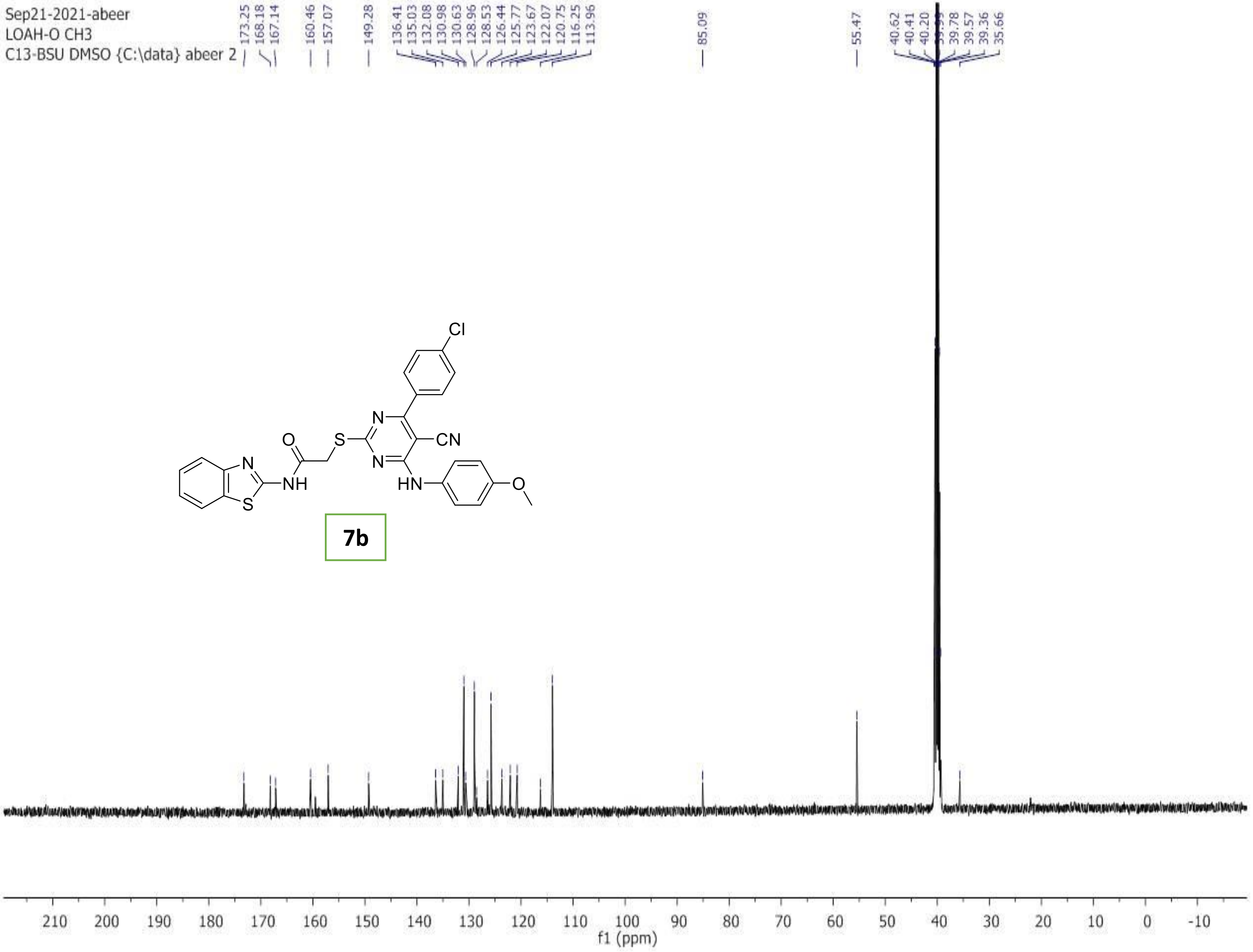
Sep21-2021-abeer
LOAH-O CH3
C13-BSU DMSO {C:\data} abeer 2
173.25
168.18
167.14
160.46
157.07
149.28
136.41
135.03
132.08
130.98
130.63
128.96
128.53
126.44
125.77
123.67
122.07
120.75
116.25
113.96
85.09
55.47
40.62
40.41
40.20
39.78
39.57
39.36
35.66
Cl
N
O
S
CN
N
N
NH
HN
O
S
7b
210
200
190
180
170
160
150
140
130
120
110
100
90
80
70
60
50
40
30
20
10
0
-10
f1 (ppm)

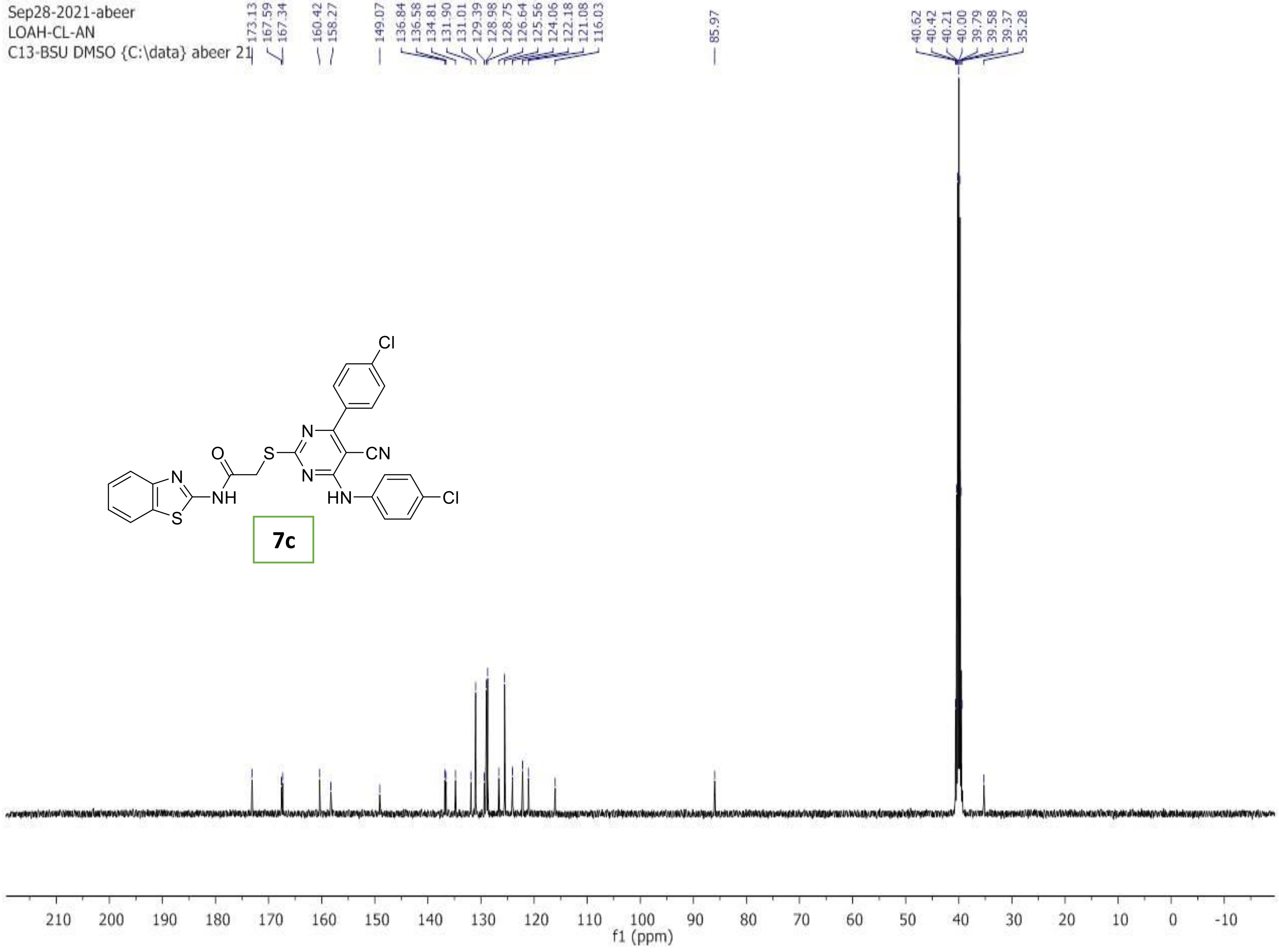

Sep28-2021-abeer
LOAH-CL-AN
C13-BSU DMSO {C:\data} abeer 21
173.13
167.59
167.34
160.42
158.27
149.07
136.84
136.58
134.81
131.90
131.01
129.39
128.98
128.75
126.64
125.56
124.06
122.18
121.08
116.03
85.97
40.62
40.42
40.21
40.00
39.79
39.58
39.37
35.28
Cl
N
O
S
CN
N
N
NH
HN
Cl
S
7c
210 200 190 180 170 160 150 140 130 120 110 100 90 80 70 60 50 40 30 20 10 0 -10
f1 (ppm)

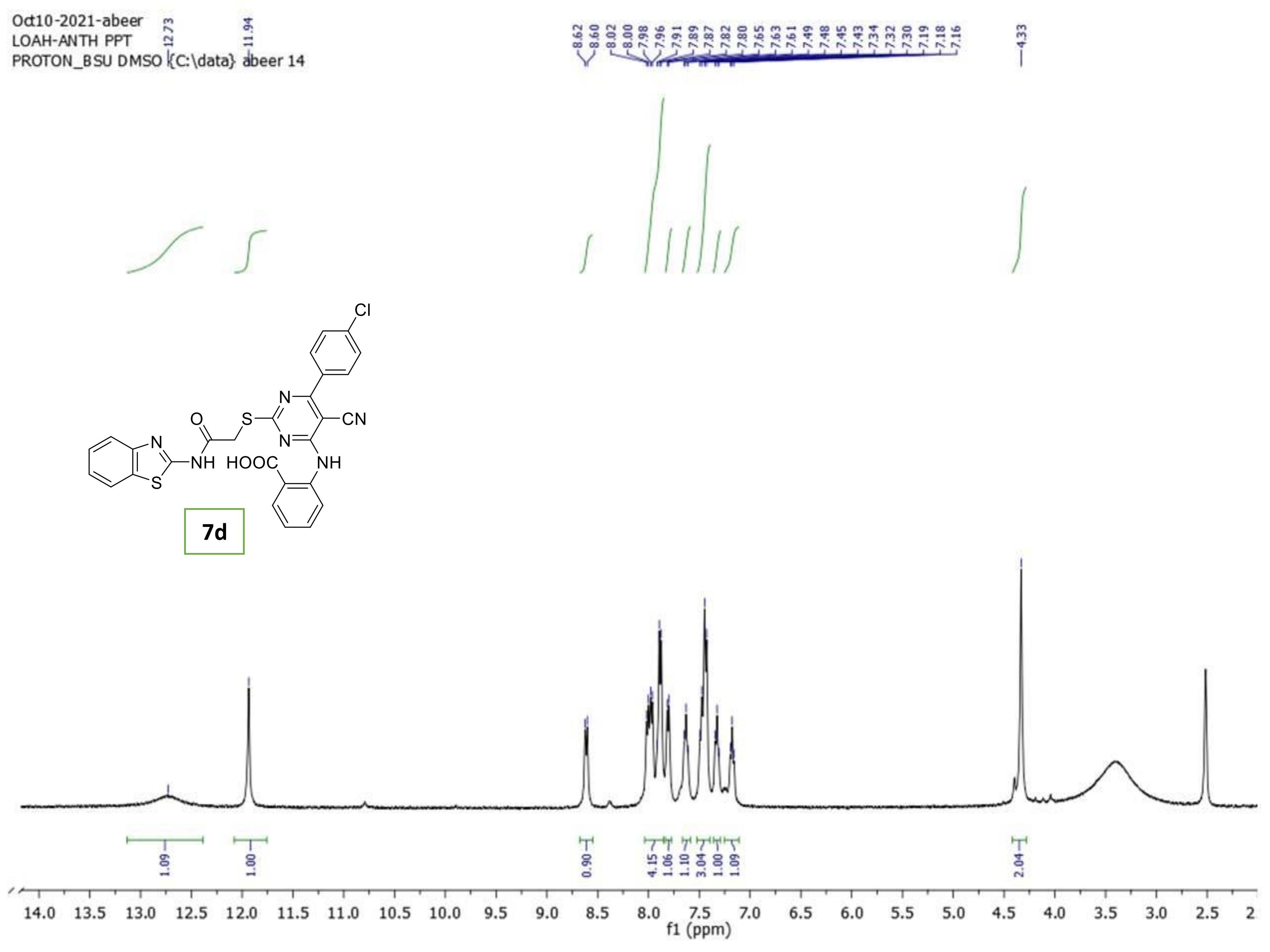
Oct10-2021-abeer
LOAH-ANTH PPT
PROTON_BSU DMSO {C:\data} abeer 14
12.73
11.94
8.62 8.60 8.02 8.00 7.98 7.96 7.91 7.89 7.87 7.82 7.80 7.65 7.63 7.61 7.49 7.48 7.45 7.43 7.34 7.32 7.30 7.19 7.18 7.16
4.33
Cl
N
CN
O
S
N
N
NH
HOOC
NH
S
7d
1.09
1.00
0.90
4.15
1.06
1.10
3.04
1.00
1.09
2.04
14.0 13.5 13.0 12.5 12.0 11.5 11.0 10.5 10.0 9.5 9.0 8.5 8.0 7.5 7.0 6.5 6.0 5.5 5.0 4.5 4.0 3.5 3.0 2.5 2
f1 (ppm)

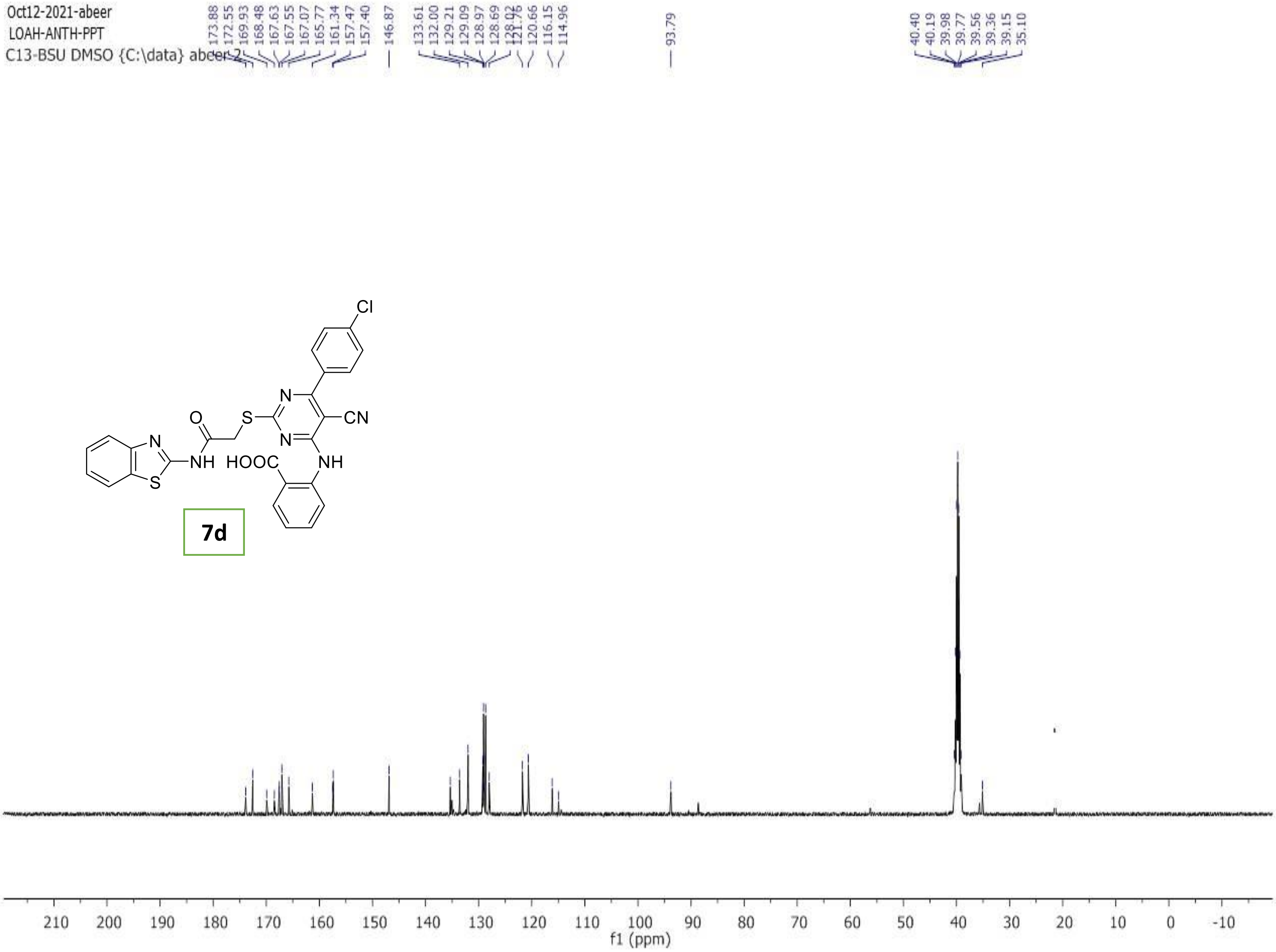
Oct12-2021-abeer
LOAH-ANTH-PPT
C13-BSU DMSO {C:\data} abeer 2
173.88
172.55
169.93
168.48
167.63
167.55
167.07
165.77
161.34
157.47
157.40
146.87
133.61
132.00
129.21
129.09
128.97
128.69
128.02
121.76
120.66
116.15
114.96
93.79
40.40
40.19
39.98
39.77
39.56
39.36
39.15
35.10
Cl
N
S
CN
O
N
N
NH
HOOC
NH
S
7d
210
200
190
180
170
160
150
140
130
120
110
100
90
80
70
60
50
40
30
20
10
0
-10
f1 (ppm)

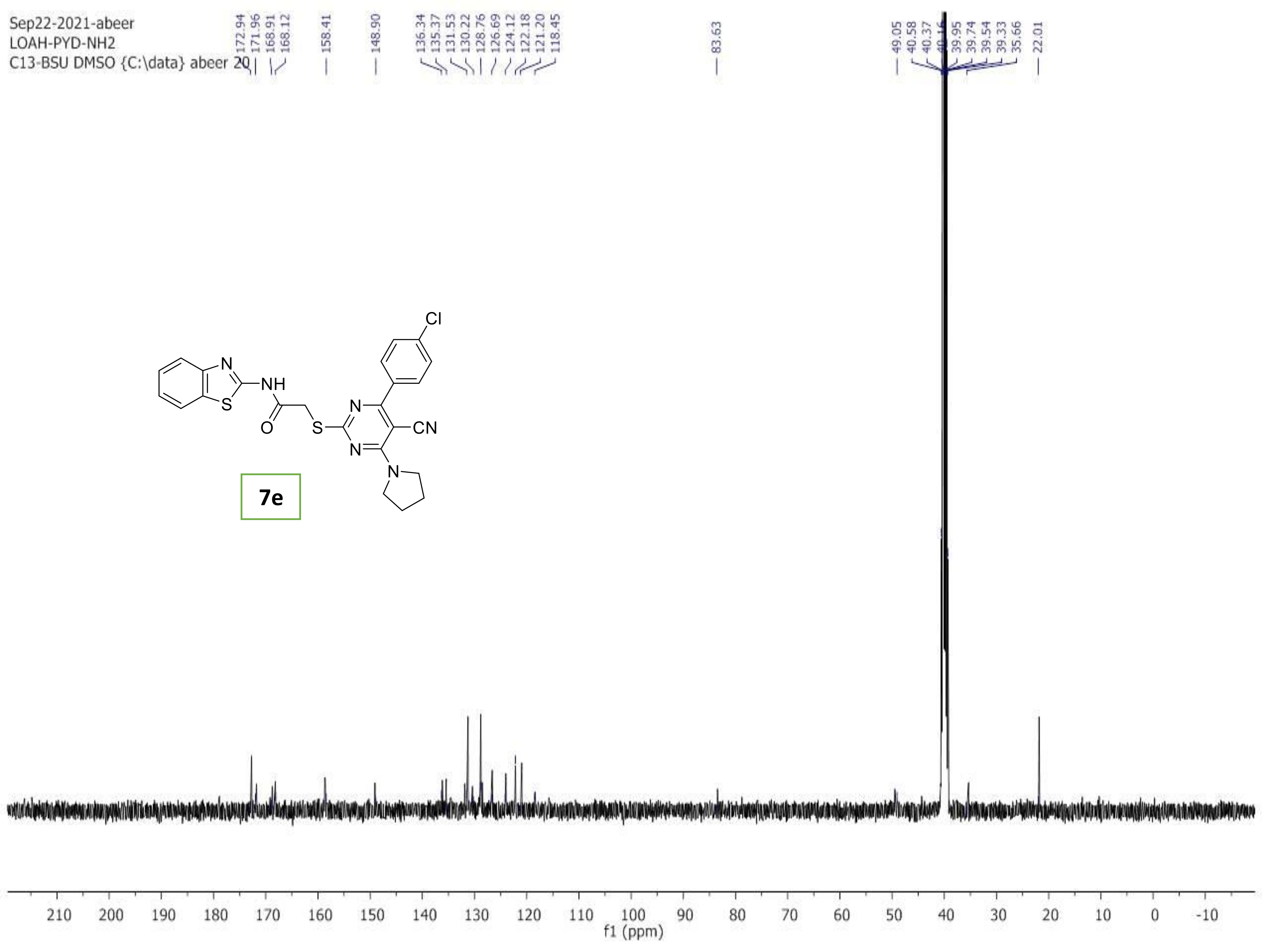
Sep22-2021-abeer
LOAH-PYD-NH2
C13-BSU DMSO {C:\data} abeer 20
172.94
171.96
168.91
168.12
158.41
148.90
136.34
135.37
131.53
130.22
128.76
126.69
124.12
122.18
121.20
118.45
83.63
49.05
40.58
40.37
40.16
39.95
39.74
39.54
39.33
35.66
22.01
Cl
N
NH
S
O
S
N
N
CN
N
7e
210
200
190
180
170
160
150
140
130
120
110
100
90
80
70
60
50
40
30
20
10
0
-10
f1 (ppm)

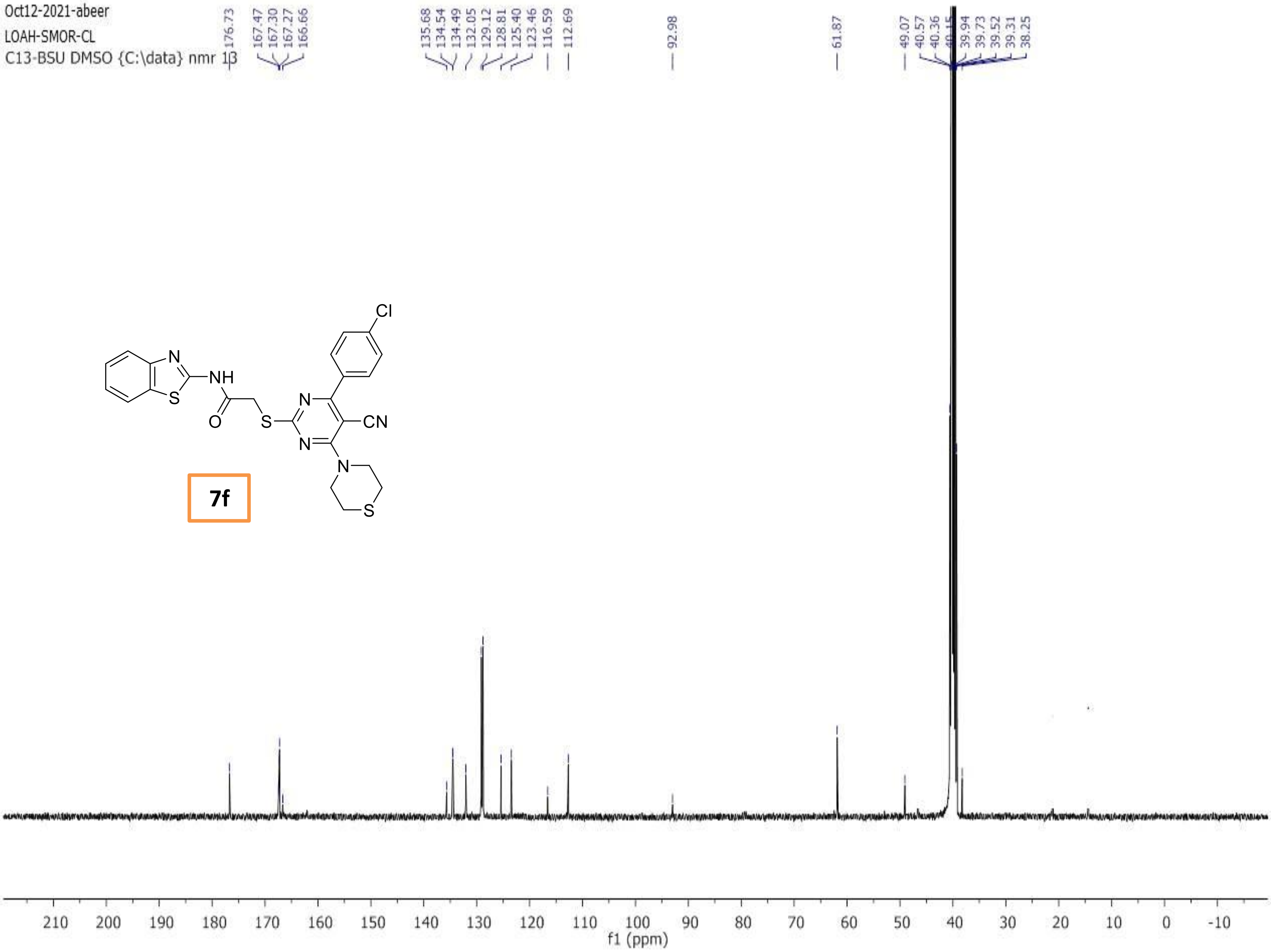
Oct12-2021-abeer
LOAH-SMOR-CL
C13-BSU DMSO {C:\data} nmr 13
176.73
167.47
167.30
167.27
166.66
135.68
134.54
134.49
132.05
129.12
128.81
125.40
123.46
116.59
112.69
92.98
61.87
49.07
40.57
40.36
40.15
39.94
39.73
39.52
39.31
38.25
Cl
N
NH
S
O
S
N
N
CN
N
S
7f
210
200
190
180
170
160
150
140
130
120
110
100
90
80
70
60
50
40
30
20
10
0
-10
f1 (ppm)

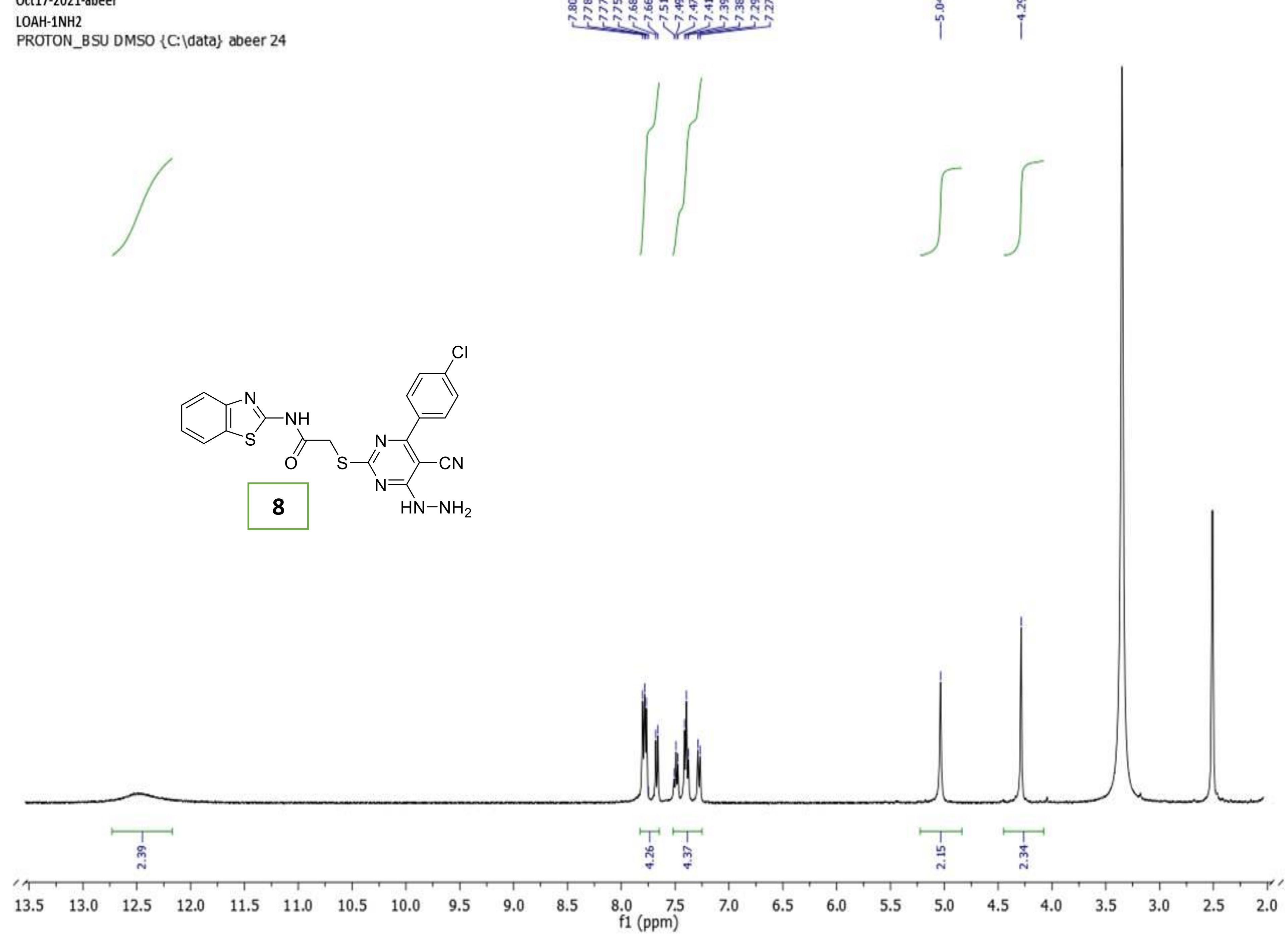

LOAH-1NH2
PROTON_BSU DMSO {C:\data} abeer 24
8
2.39
4.26
4.37
2.15
2.34
f1 (ppm)

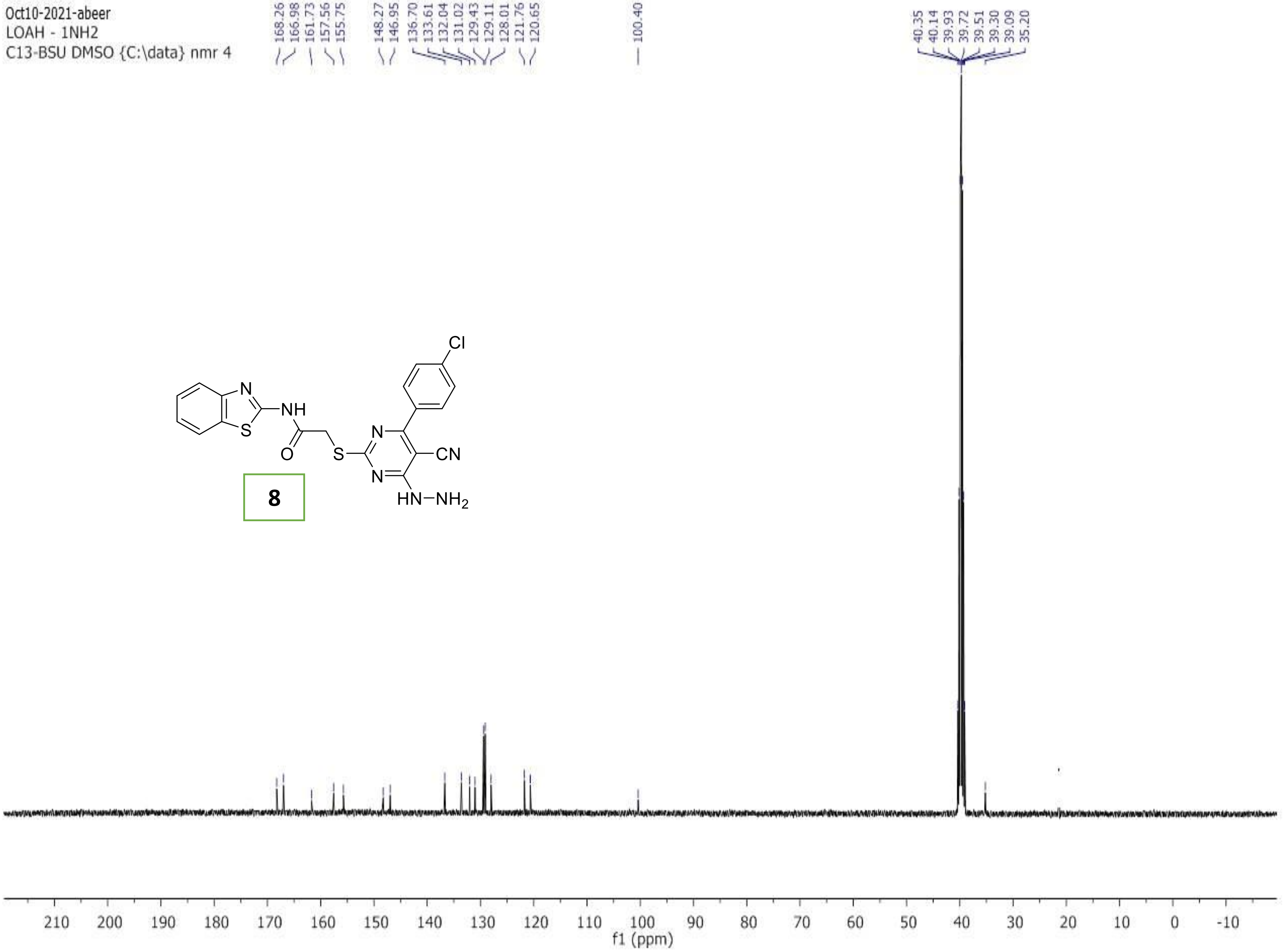
Oct10-2021-abeer
LOAH - 1NH2
C13-BSU DMSO {C:\data} nmr 4
168.26
166.98
161.73
157.56
155.75
148.27
146.95
136.70
133.61
132.04
131.02
129.43
129.11
128.01
121.76
120.65
100.40
40.35
40.14
39.93
39.72
39.51
39.30
39.09
35.20
210
200
190
180
170
160
150
140
130
120
110
100
90
80
70
60
50
40
30
20
10
0
-10
f1 (ppm)
N
NH
S
O
S
N
N
Cl
CN
HN−NH2

8

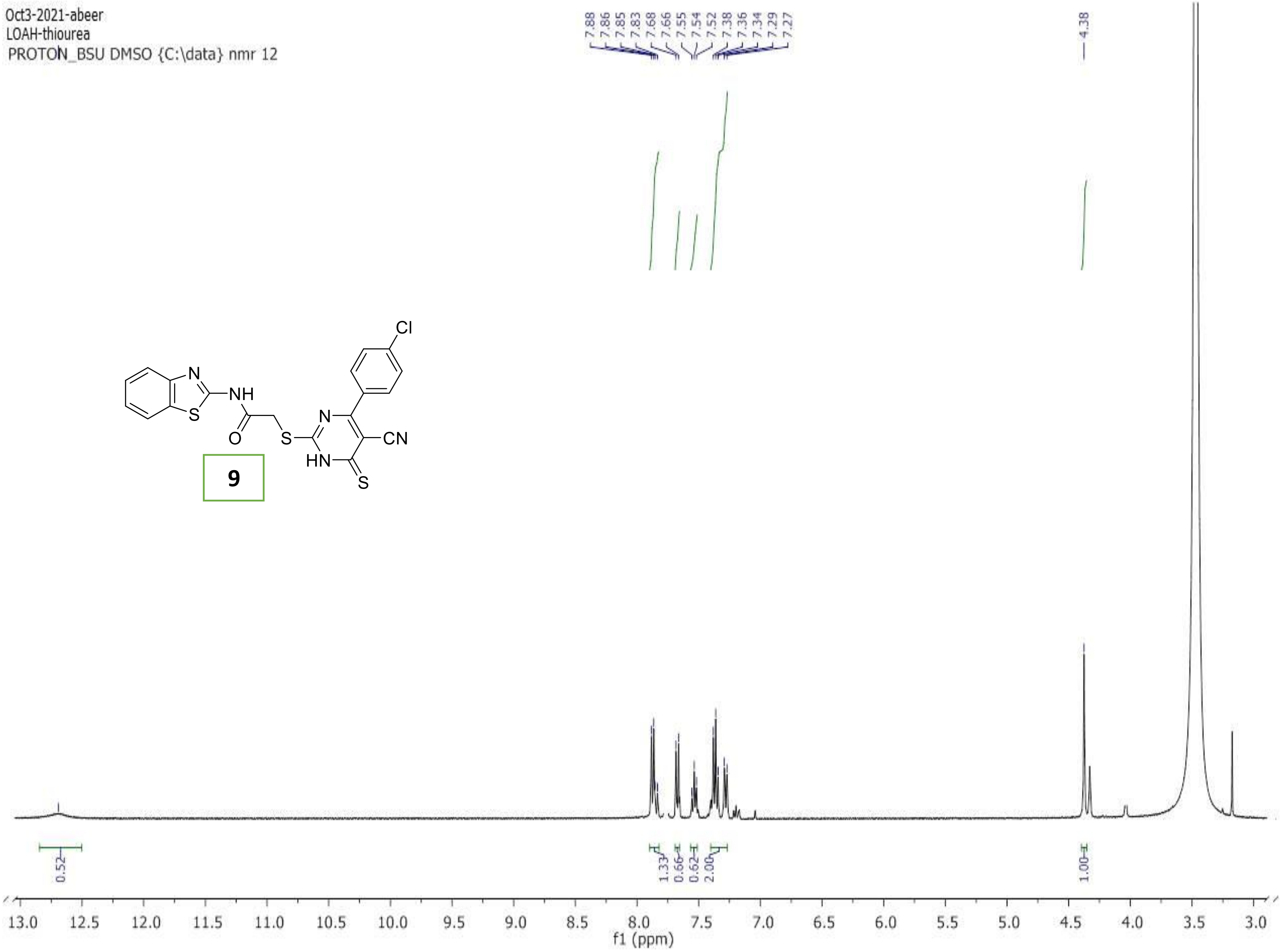

Oct3-2021-abeer
LOAH-thiourea
PROTON_BSU DMSO {C:\data} nmr 12
7.88
7.86
7.85
7.83
7.68
7.66
7.55
7.54
7.52
7.38
7.36
7.34
7.29
7.27
4.38
Cl
N
NH
S
O
N
S
CN
HN
S
9
0.52
1.33
0.66
0.62
2.00
1.00
13.0
12.5
12.0
11.5
11.0
10.5
10.0
9.5
9.0
8.5
8.0
7.5
7.0
6.5
6.0
5.5
5.0
4.5
4.0
3.5
3.0
f1 (ppm)

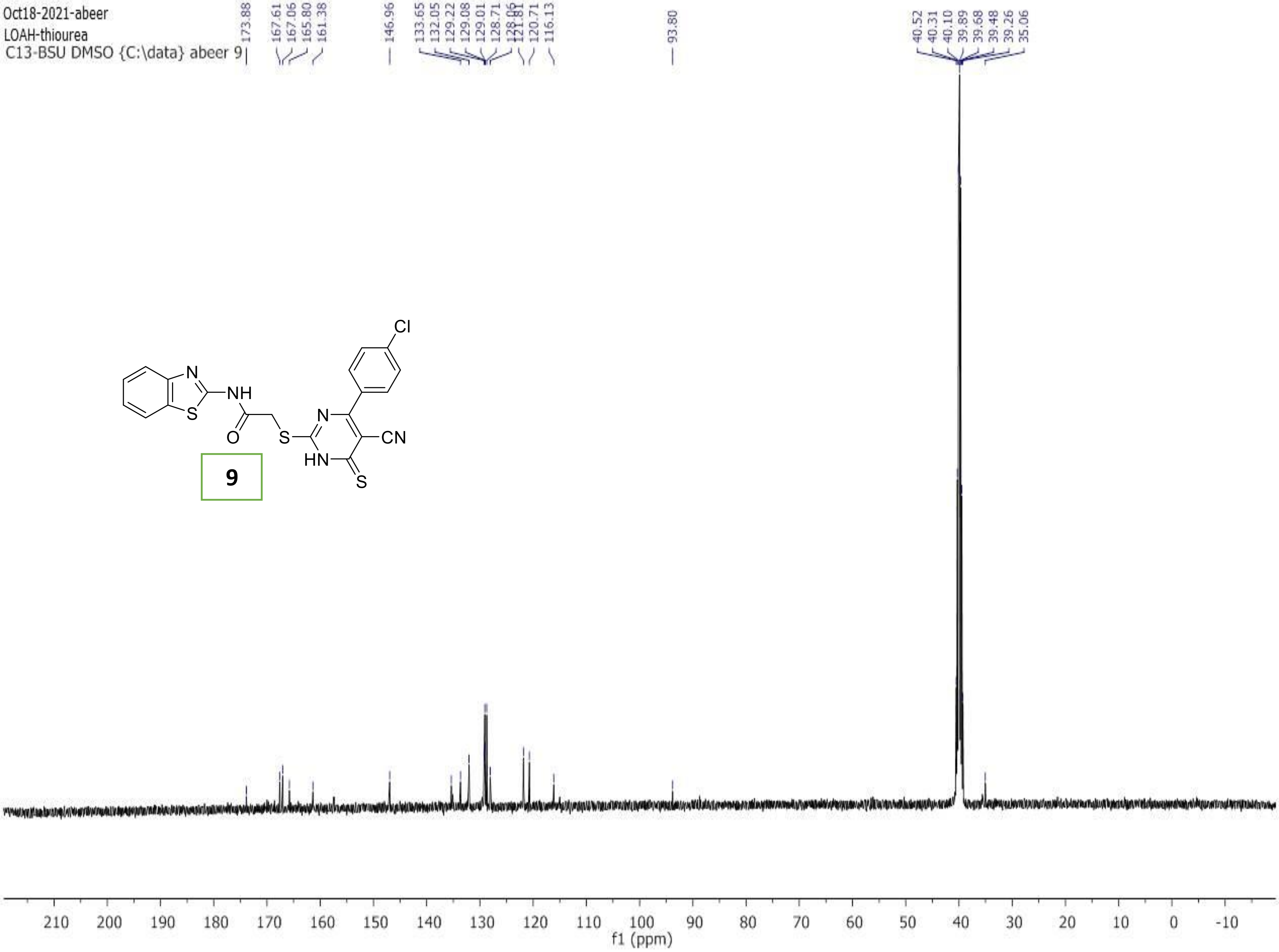
Oct18-2021-abeer
LOAH-thiourea
C13-BSU DMSO {C:\data} abeer 9
173.88
167.61
167.06
165.80
161.38
146.96
133.65
132.05
129.22
129.08
129.01
128.71
121.81
120.71
116.13
93.80
40.52
40.31
40.10
39.89
39.68
39.48
39.26
35.06
Cl
N
NH
S
O
S
N
HN
CN
S
9
f1 (ppm)

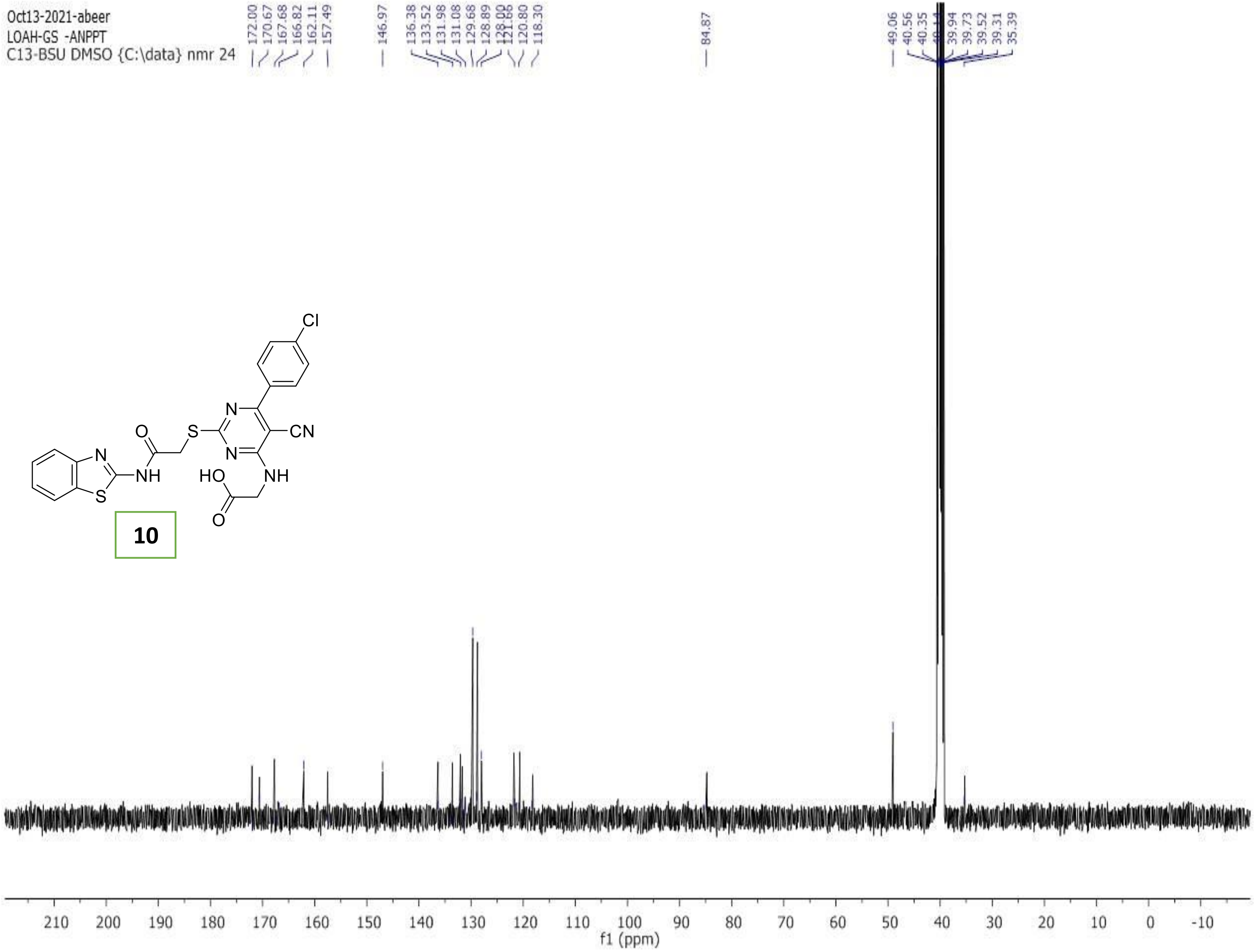

Oct13-2021-abeer
LOAH-GS -ANPPT
C13-BSU DMSO {C:\data} nmr 24
172.00
170.67
167.68
166.82
162.11
157.49
146.97
136.38
133.52
131.98
131.08
129.68
128.89
120.80
118.30
84.87
49.06
40.56
40.35
39.94
39.73
39.52
39.31
35.39
Cl
N
S
O
CN
N
NH
HO
NH
S
O
10
210
200
190
180
170
160
150
140
130
120
110
100
90
80
70
60
50
40
30
20
10
0
-10
f1 (ppm)

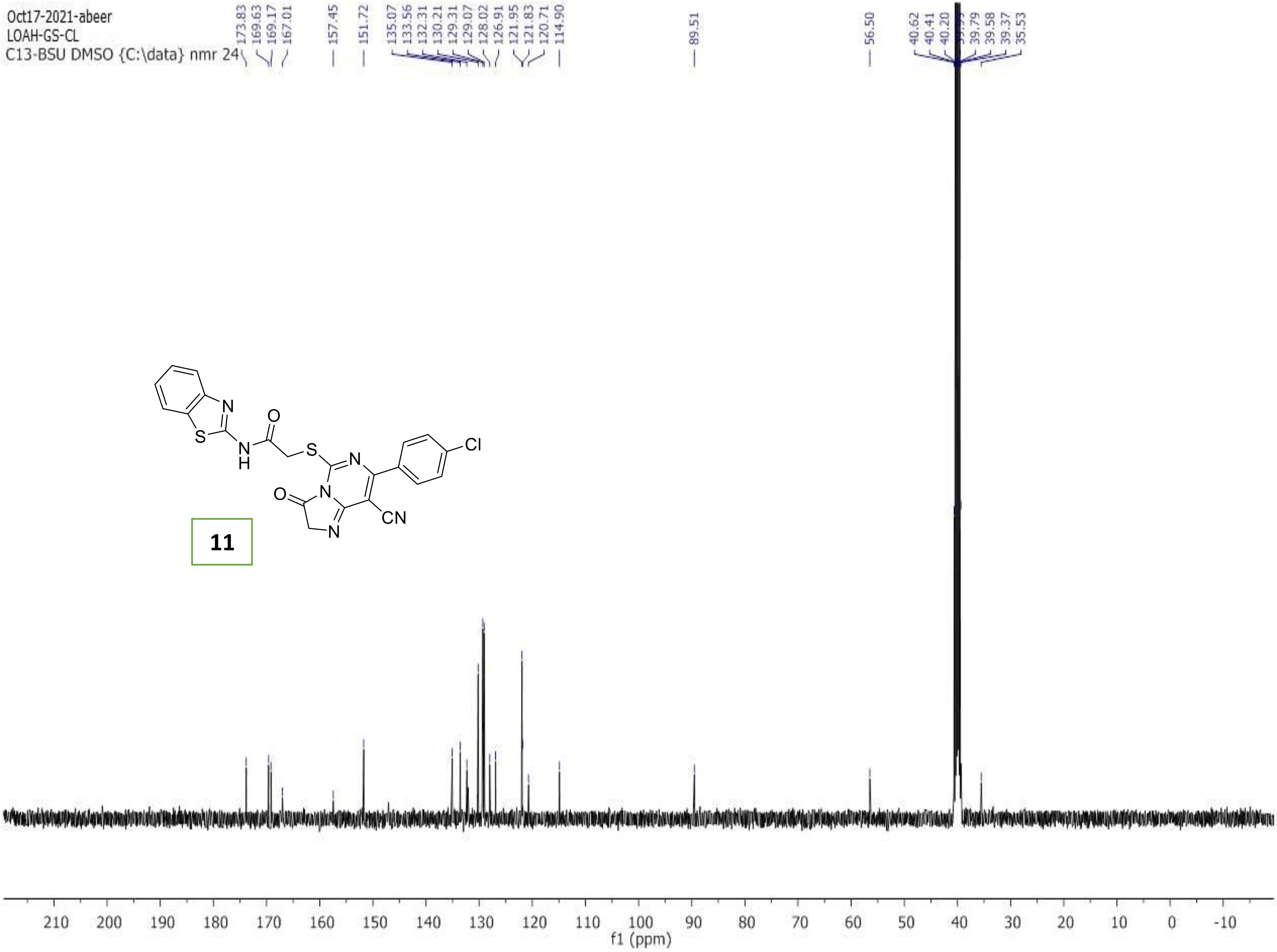
Oct17-2021-abeer
LOAH-GS-CL
C13-BSU DMSO {C:\data} nmr 24
173.83
169.63
169.17
167.01
157.45
151.72
135.07
133.56
132.31
130.21
129.31
129.07
128.02
126.91
121.95
121.83
120.71
114.90
89.51
56.50
40.62
40.41
40.20
39.79
39.58
39.37
35.53
N
O
S
N
H
S
N
Cl
O
N
CN
N
11
210
200
190
180
170
160
150
140
130
120
110
100
90
80
70
60
50
40
30
20
10
0
-10
f1 (ppm)

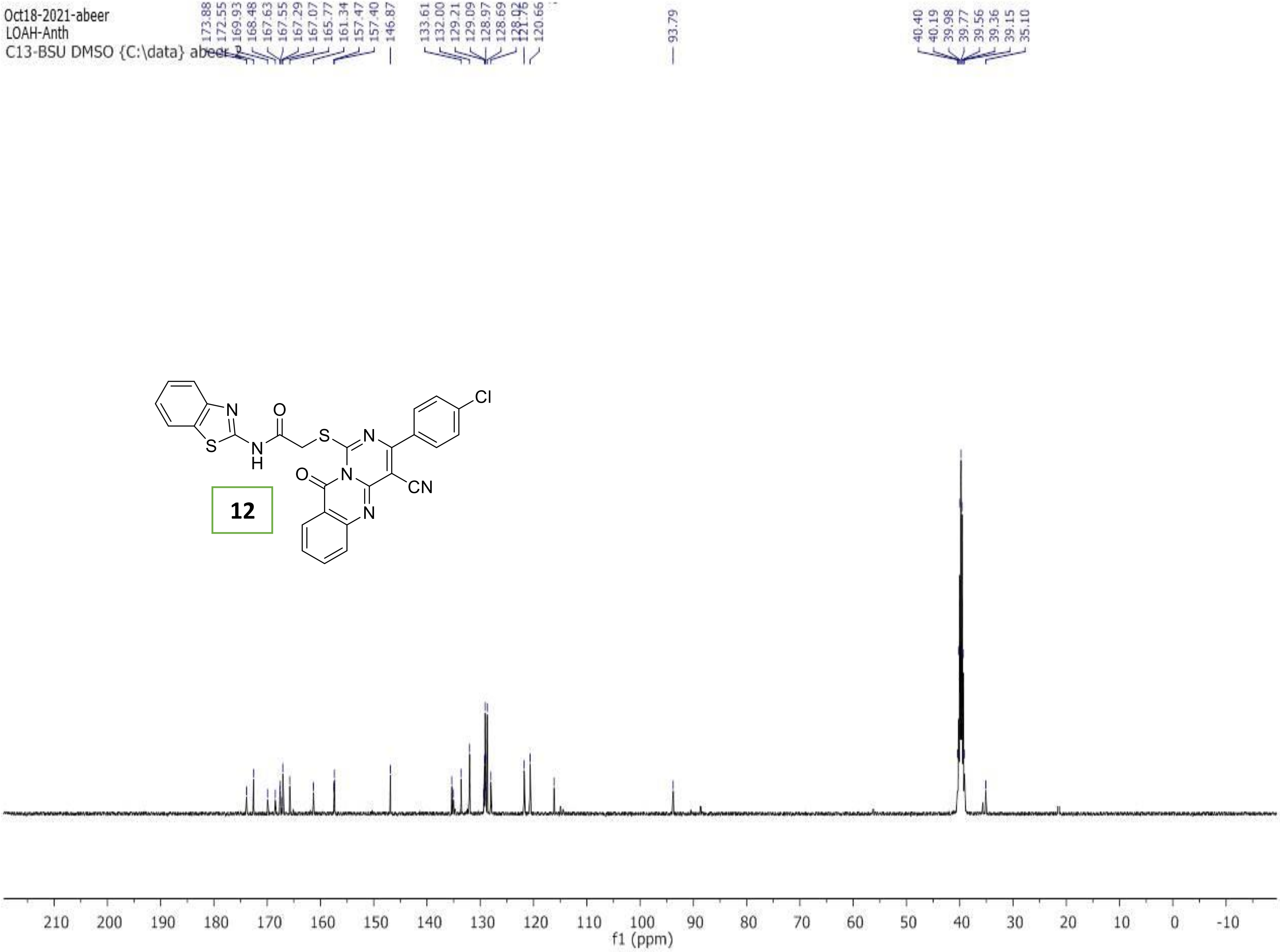

Oct18-2021-abeer
LOAH-Anth
C13-BSU DMSO {C:\data} abeer
173.88
172.55
169.93
168.48
167.63
167.55
167.29
167.07
165.77
161.34
157.47
157.40
146.87
133.61
132.00
129.21
129.09
128.97
128.69
128.02
121.76
120.66
93.79
40.40
40.19
39.98
39.77
39.56
39.36
39.15
35.10
N
O
S
N
H
N
Cl
O
N
CN
N
12
210 200 190 180 170 160 150 140 130 120 110 100 90 80 70 60 50 40 30 20 10 0 -10
f1 (ppm)

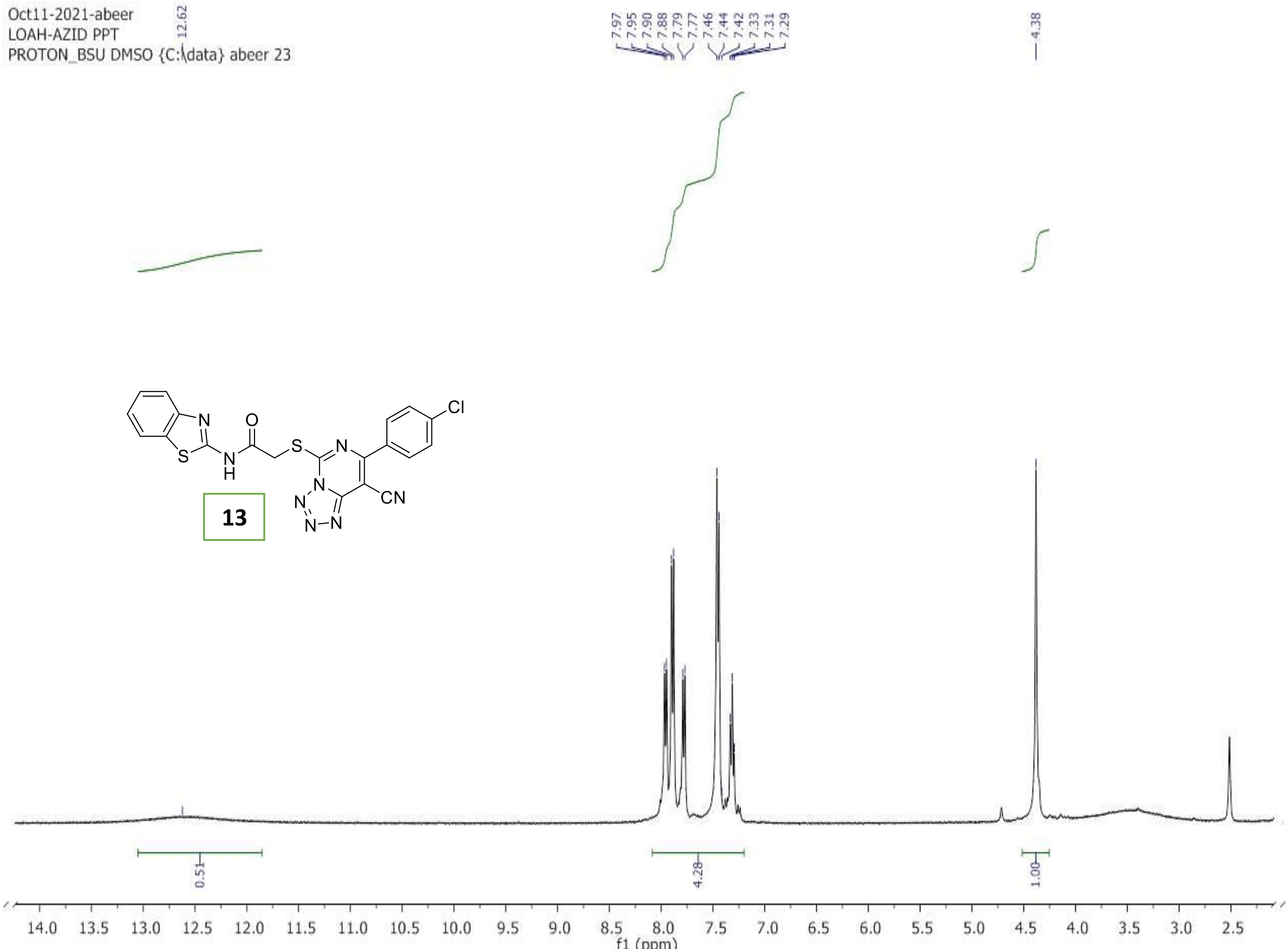

Oct11-2021-abeer
LOAH-AZID PPT
PROTON_BSU DMSO {C:\data} abeer 23
12.62
7.97
7.95
7.90
7.88
7.79
7.77
7.46
7.44
7.42
7.33
7.31
7.29
4.38
0.51
4.28
1.00
14.0
13.5
13.0
12.5
12.0
11.5
11.0
10.5
10.0
9.5
9.0
8.5
8.0
7.5
7.0
6.5
6.0
5.5
5.0
4.5
4.0
3.5
3.0
2.5
f1 (ppm)
N
S
N
H
O
S
N
N
N
N
N
N
Cl
CN
13

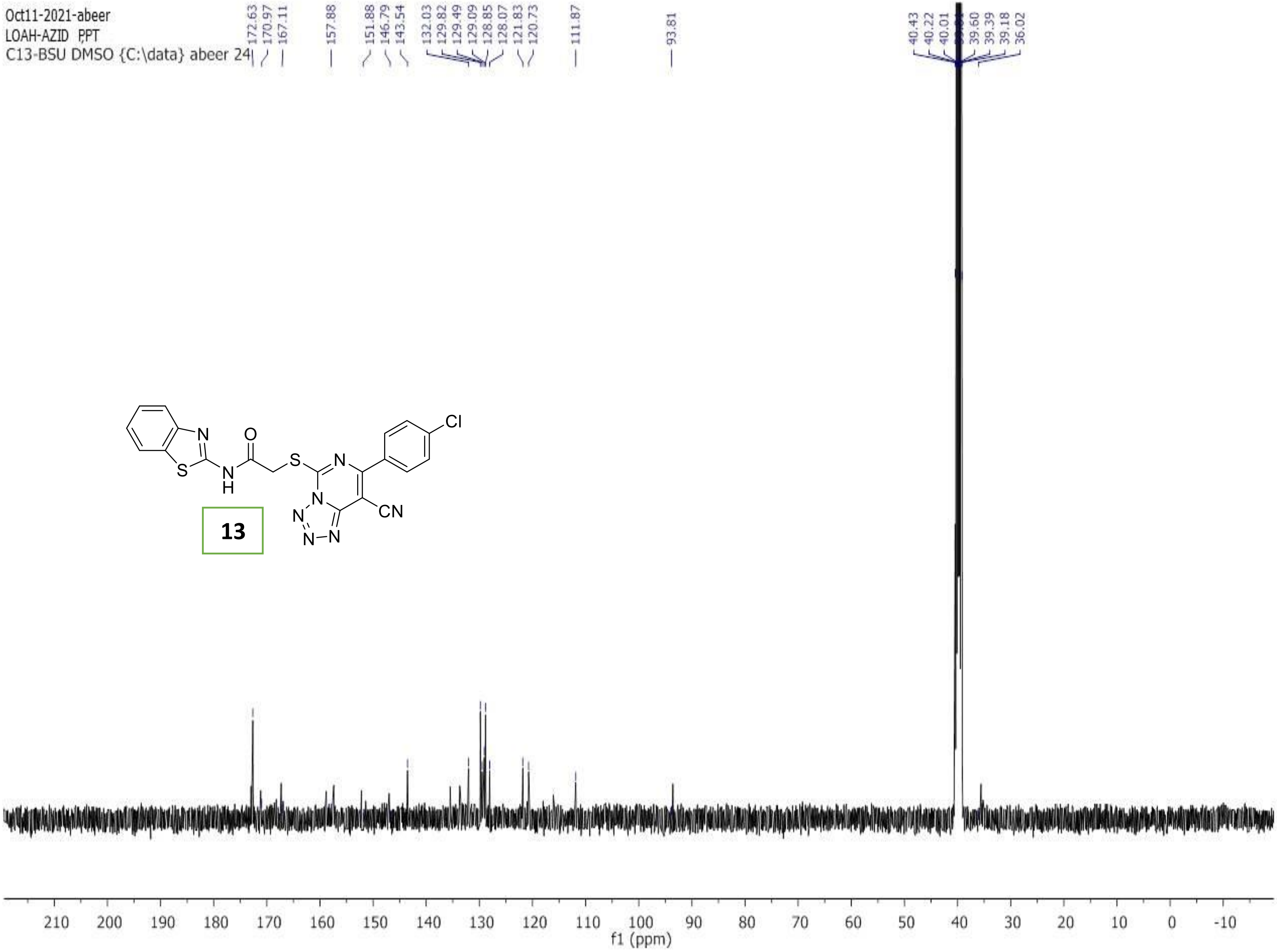
Oct11-2021-abeer
LOAH-AZID PPT
C13-BSU DMSO {C:\data} abeer 24
172.63
170.97
167.11
157.88
151.88
146.79
143.54
132.03
129.82
129.49
129.09
128.85
128.07
121.83
120.73
111.87
93.81
40.43
40.22
40.01
39.60
39.39
39.18
36.02
N
O
S
N
H
S
N
Cl
N
CN
N
N
N
210 200 190 180 170 160 150 140 130 120 110 100 90 80 70 60 50 40 30 20 10 0 -10
f1 (ppm)

13

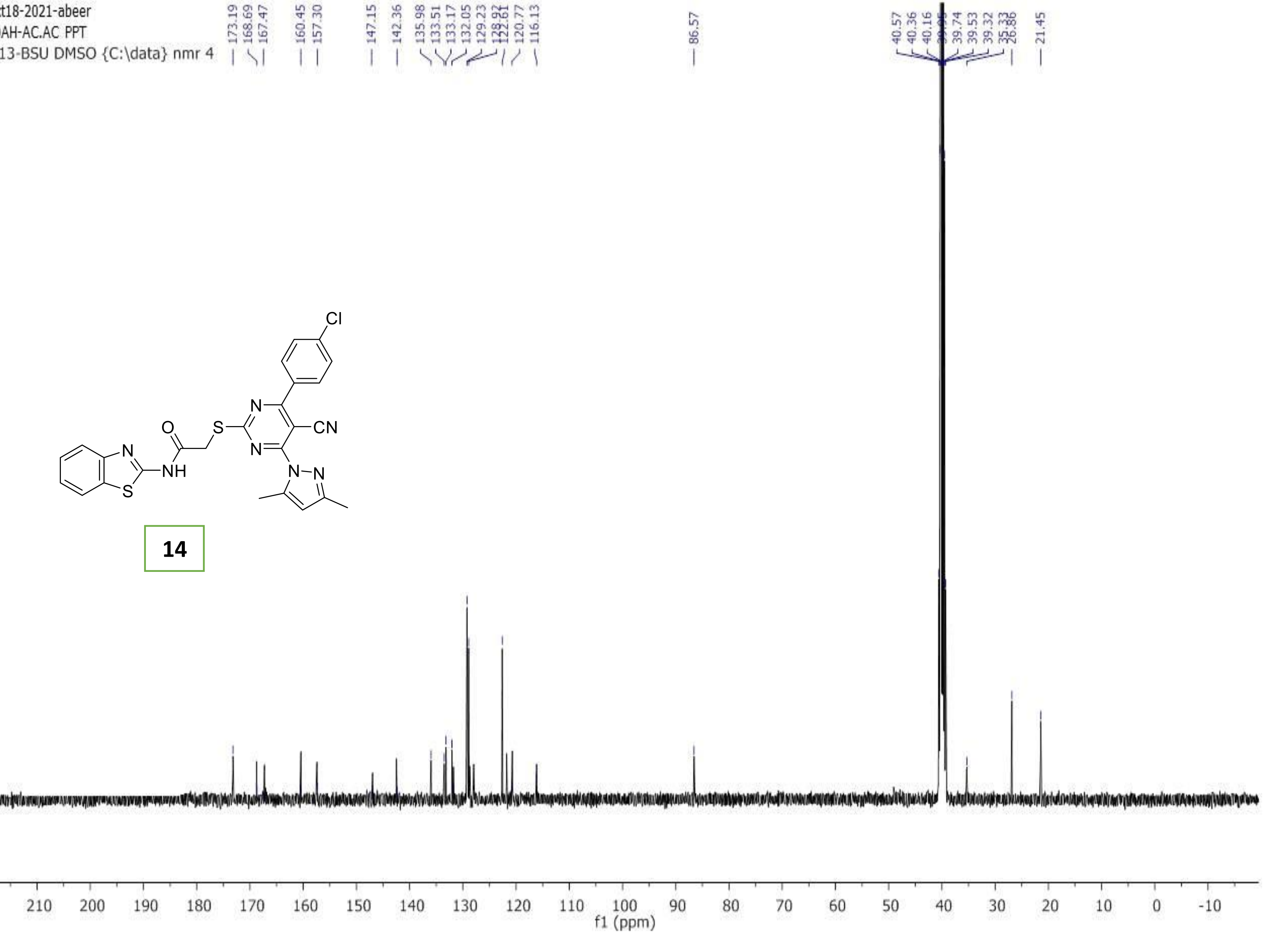

t18-2021-abeer
DAH-AC.AC PPT
13-BSU DMSO {C:\data} nmr 4
173.19
168.69
167.47
160.45
157.30
147.15
142.36
135.98
133.51
133.17
132.05
129.23
128.92
122.61
120.77
116.13
86.57
40.57
40.36
40.16
39.74
39.53
39.32
35.33
26.86
21.45
Cl
N
O
S
CN
N
NH
N
S
N
N
14
210 200 190 180 170 160 150 140 130 120 110 100 90 80 70 60 50 40 30 20 10 0 -10
f1 (ppm)

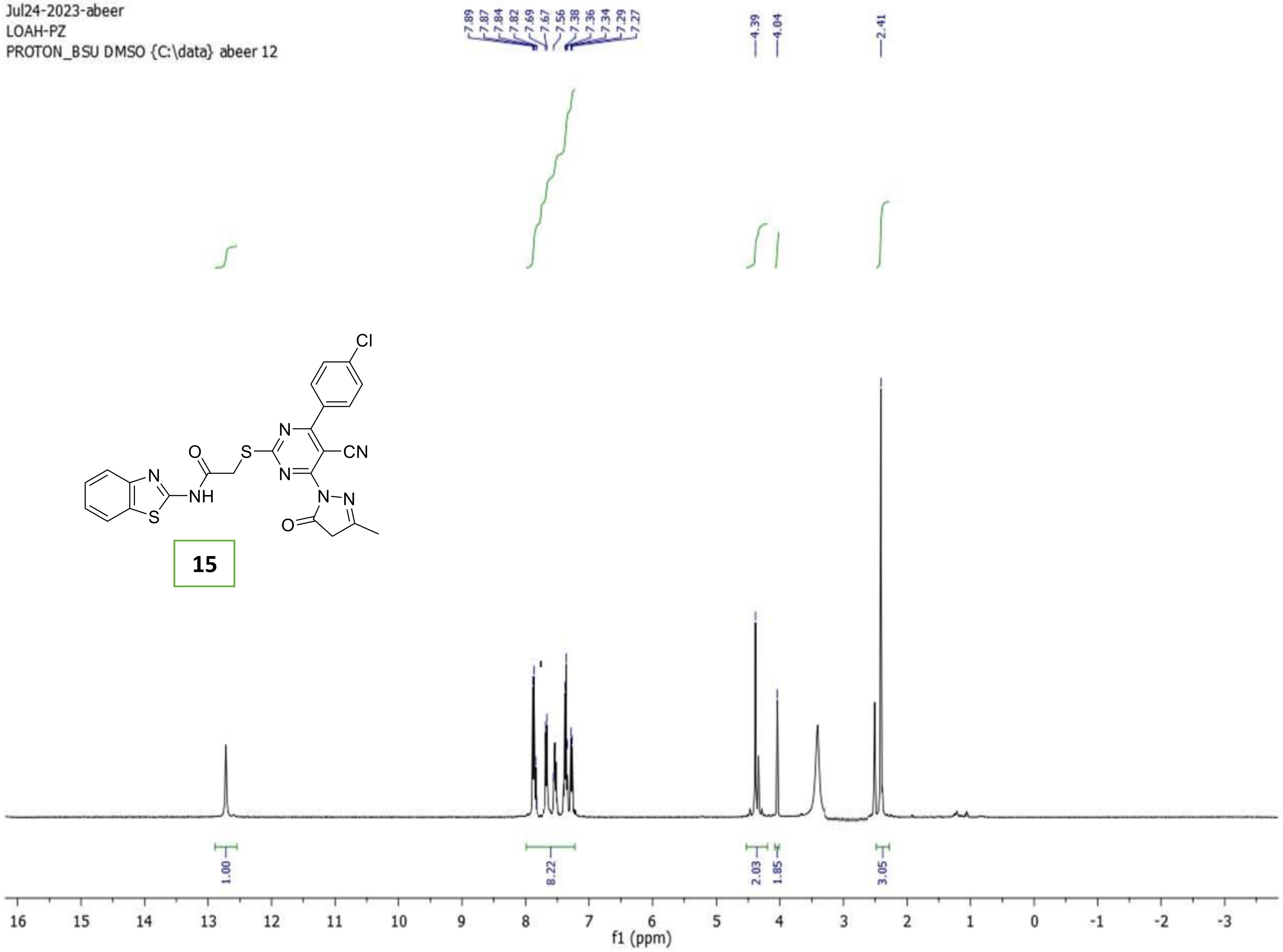
Jul24-2023-abeer
LOAH-PZ
PROTON_BSU DMSO {C:\data} abeer 12
7.89 7.87 7.84 7.82 7.69 7.67 7.56 7.38 7.36 7.34 7.29 7.27
4.39
4.04
2.41
Cl
N
S
CN
O
N
NH
N
N
N
S
O
1.00
8.22
2.03
1.85
3.05
16 15 14 13 12 11 10 9 8 7 6 5 4 3 2 1 0 -1 -2 -3
f1 (ppm)

15

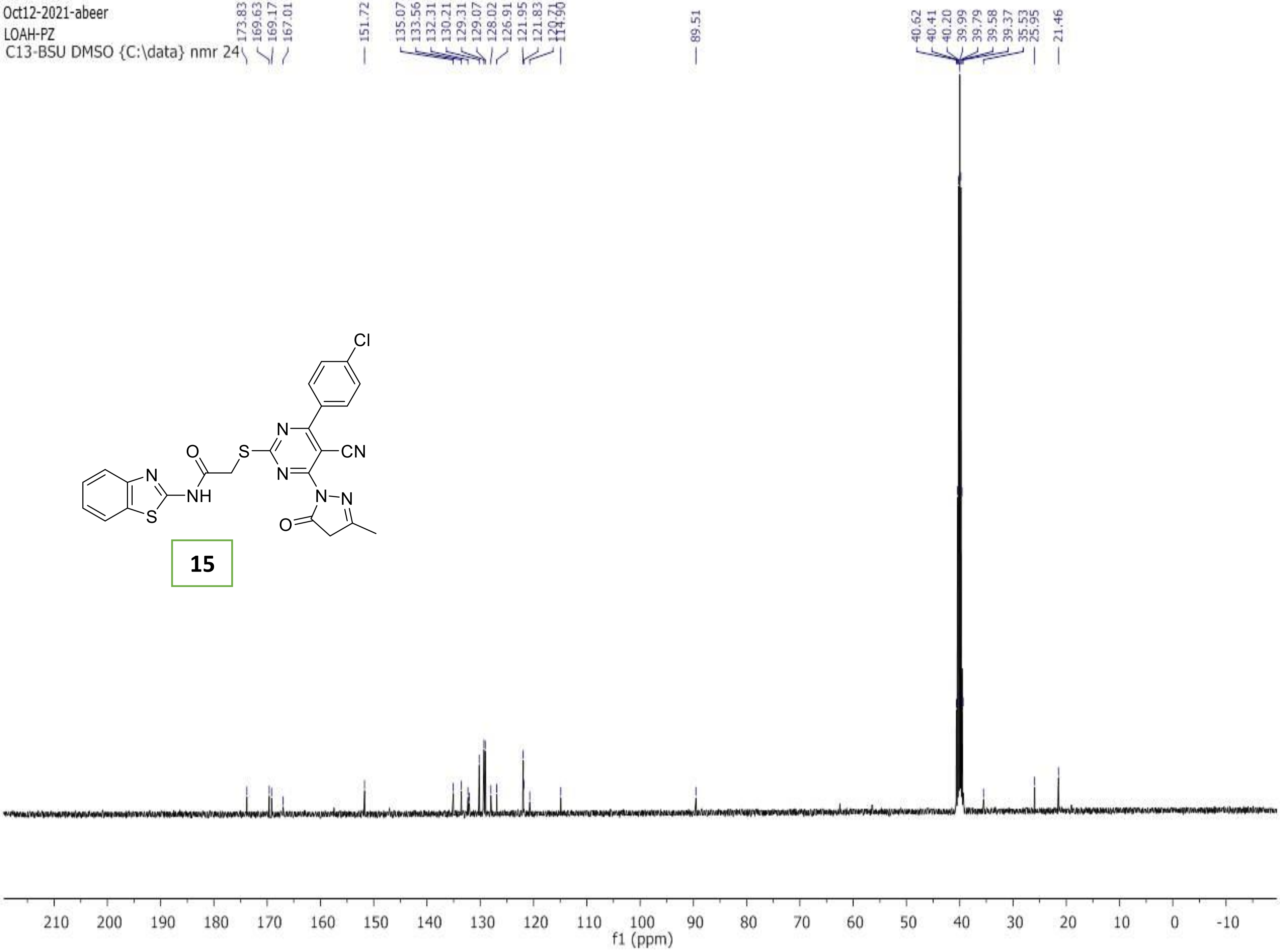

Oct12-2021-abeer
LOAH-PZ
C13-BSU DMSO {C:\data} nmr 24
173.83
169.63
169.17
167.01
151.72
135.07
133.56
132.31
130.21
129.31
129.07
128.02
126.91
121.95
121.83
120.71
114.90
89.51
40.62
40.41
40.20
39.99
39.79
39.58
39.37
35.53
25.95
21.46
Cl
N
N
S
O
N
NH
S
CN
N
N
O
15
210 200 190 180 170 160 150 140 130 120 110 100 90 80 70 60 50 40 30 20 10 0 -10
f1 (ppm)

Compound No. 5a

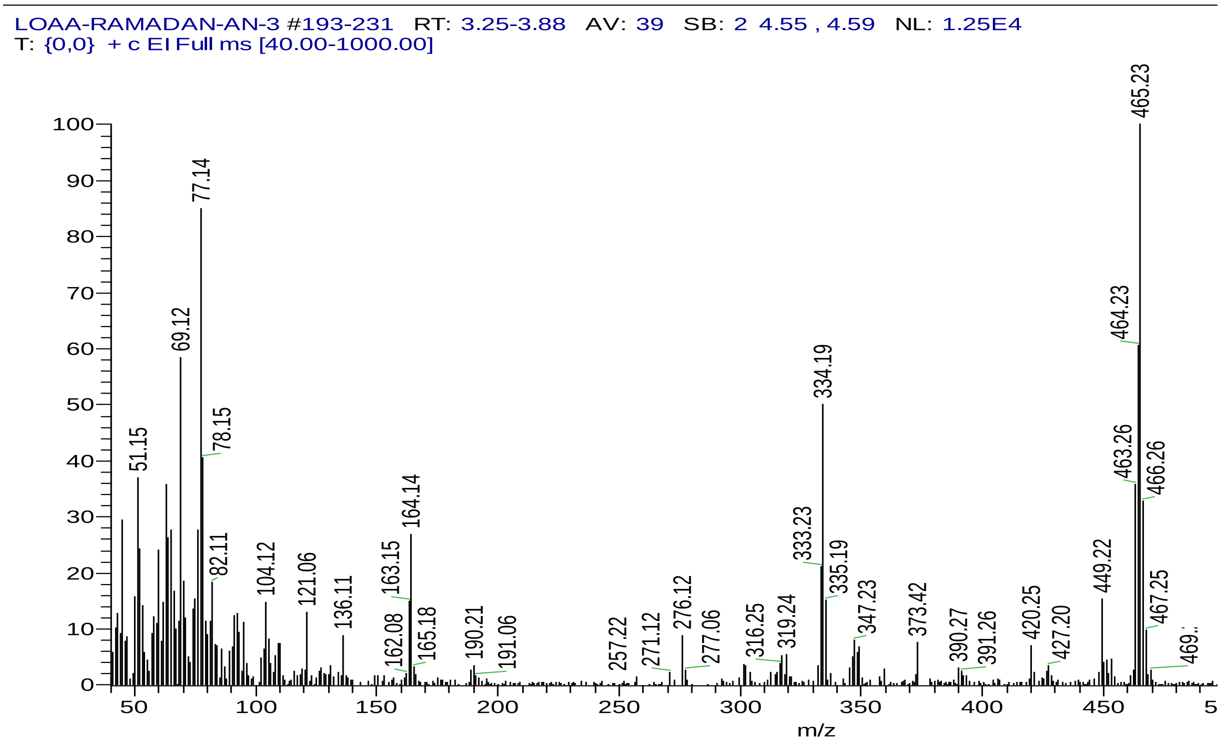

Compound No. 5b

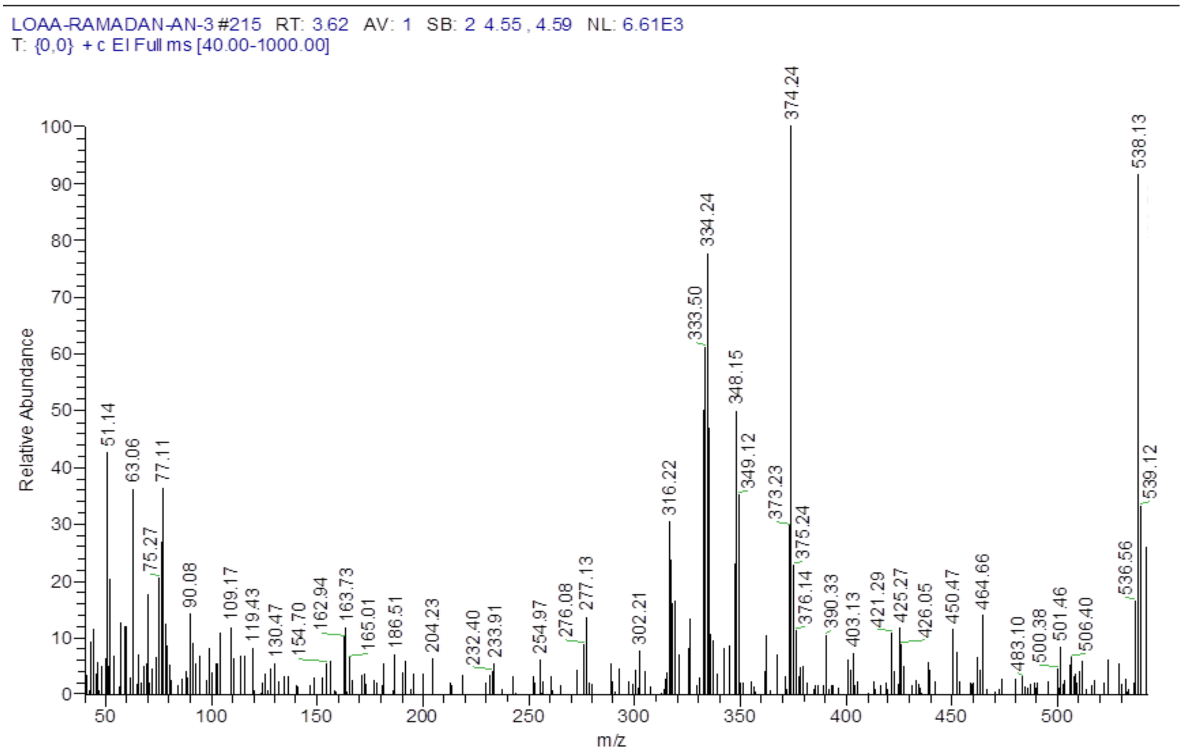

Compound No. 5c

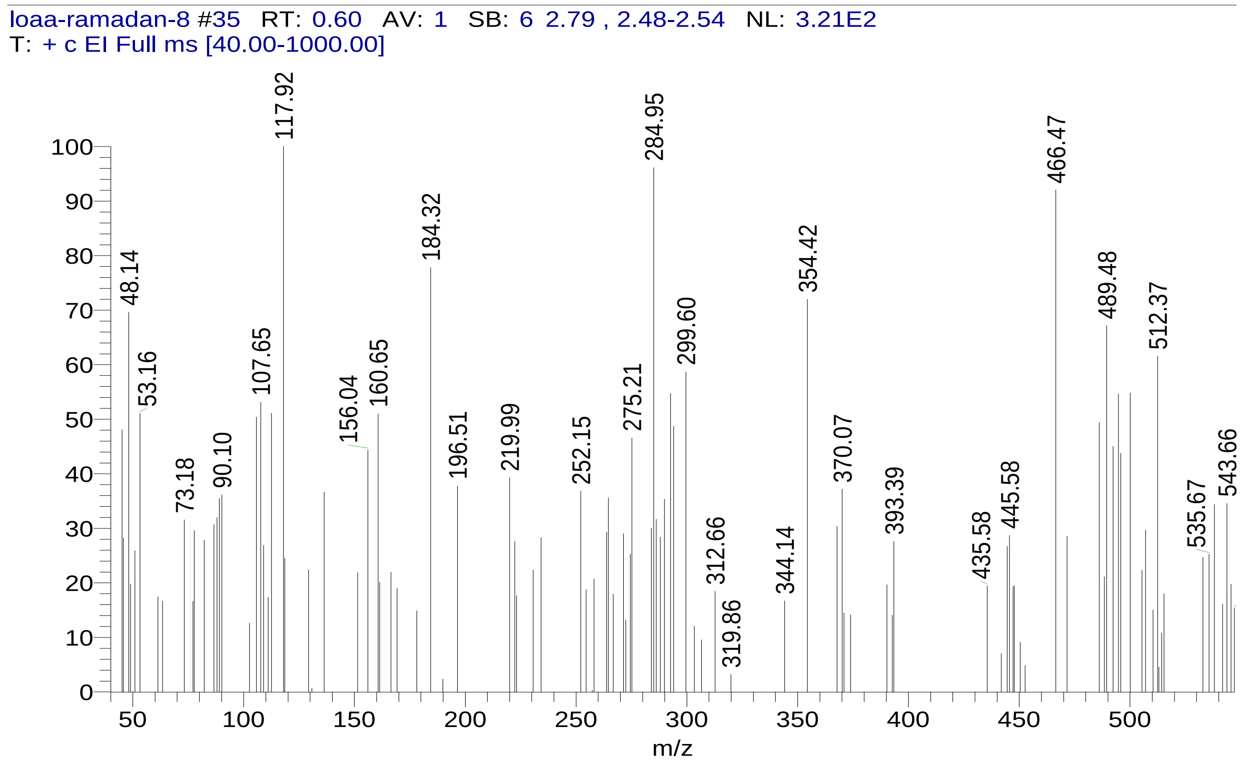

Compound No. 6

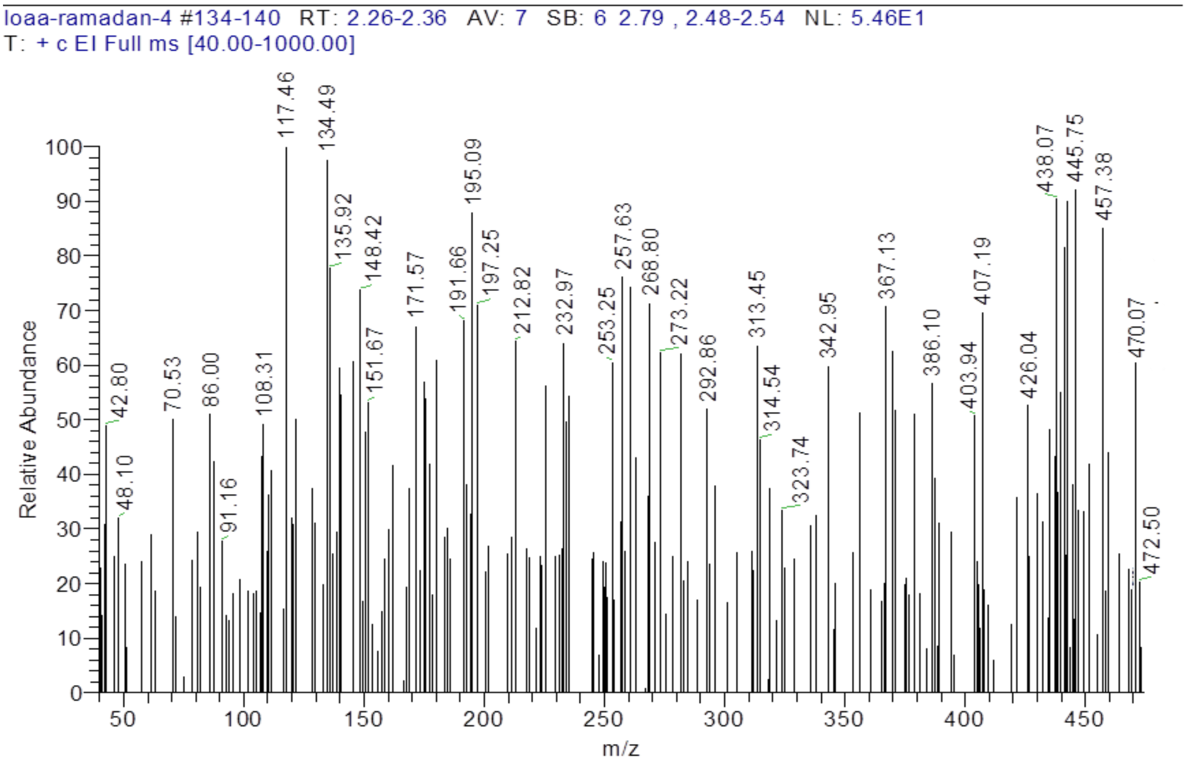

Compound 7a

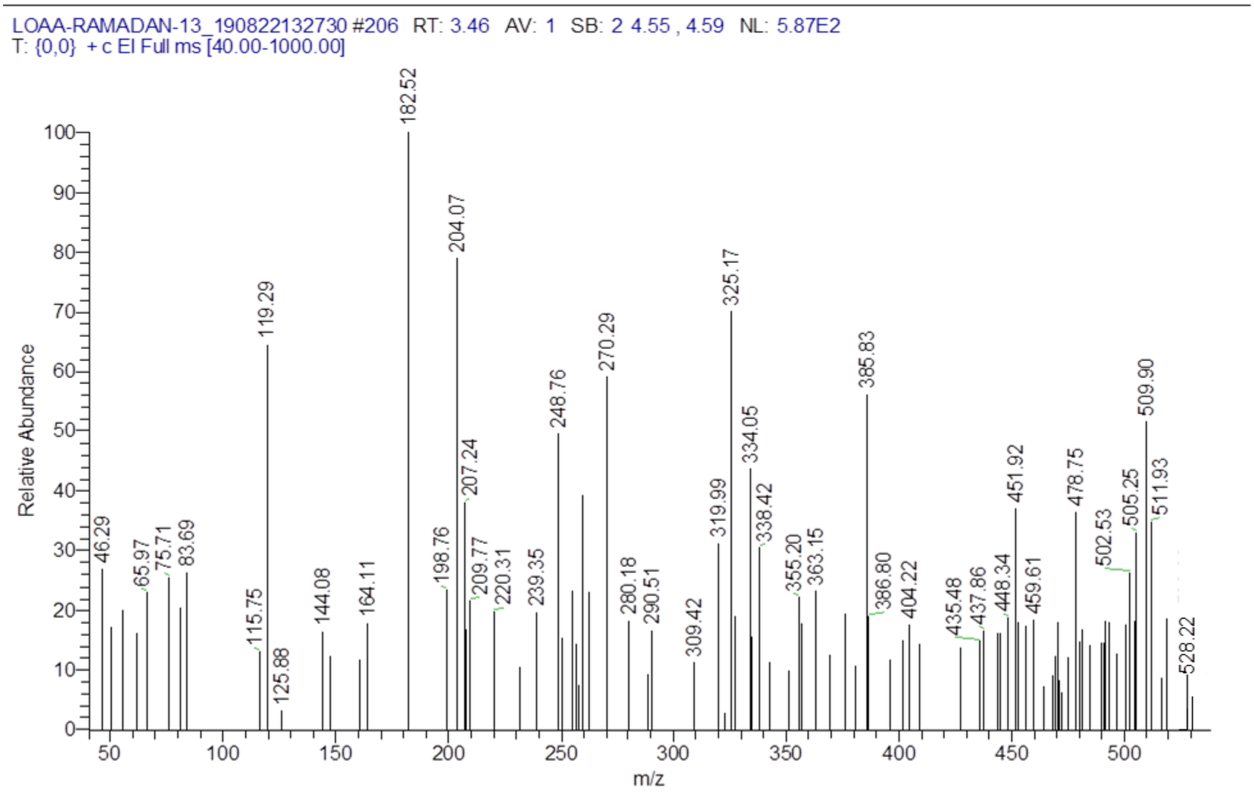

Compound 7c

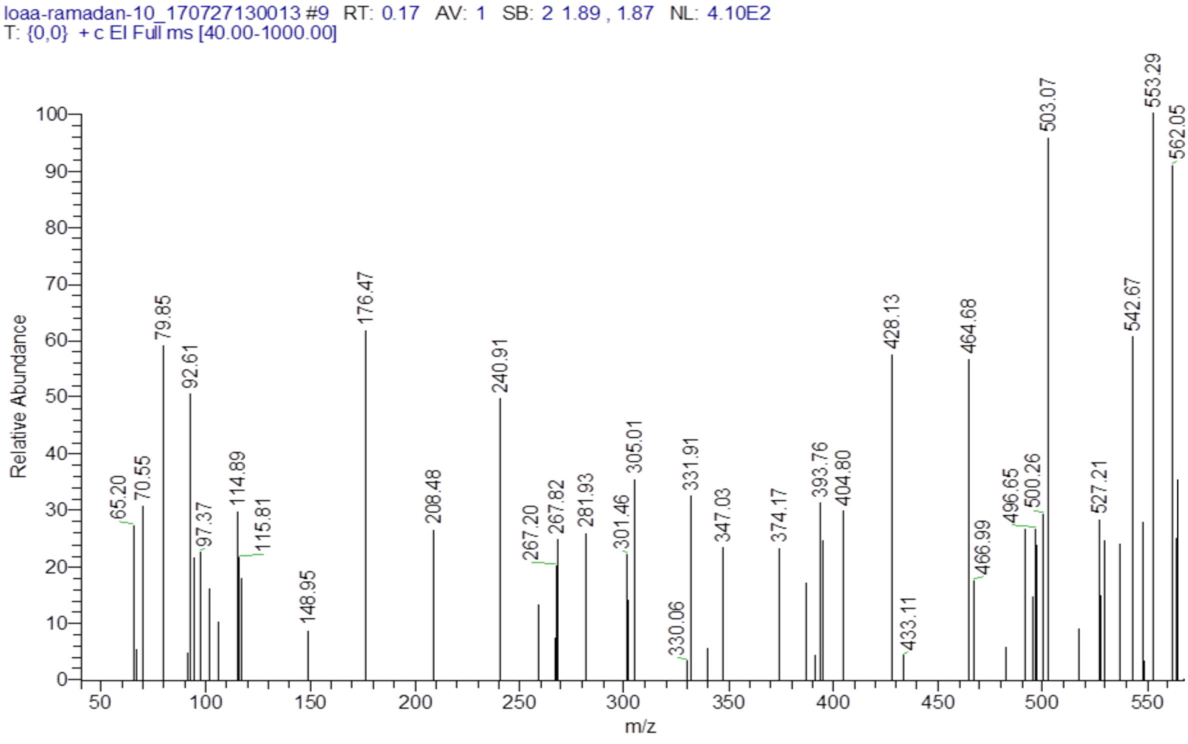

Compound 7d

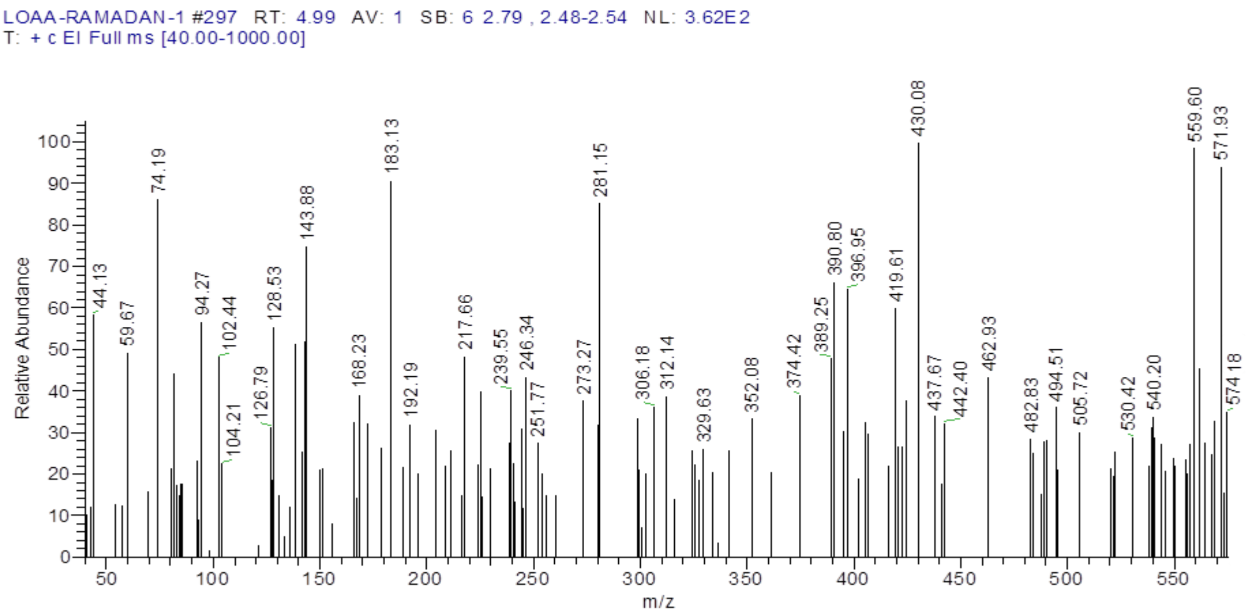

Compound 8

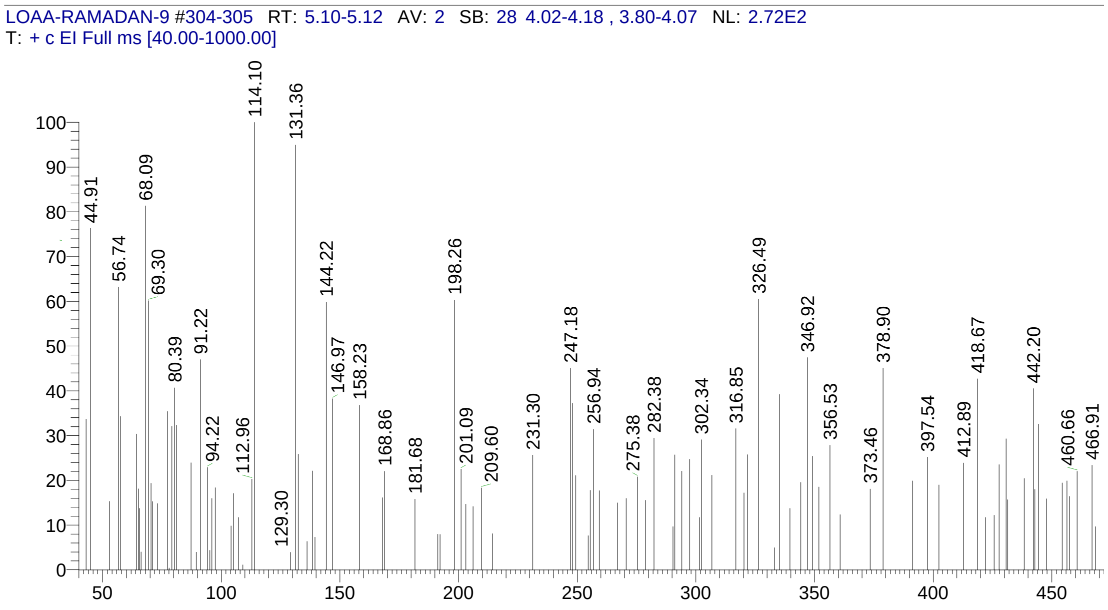

Compound 9

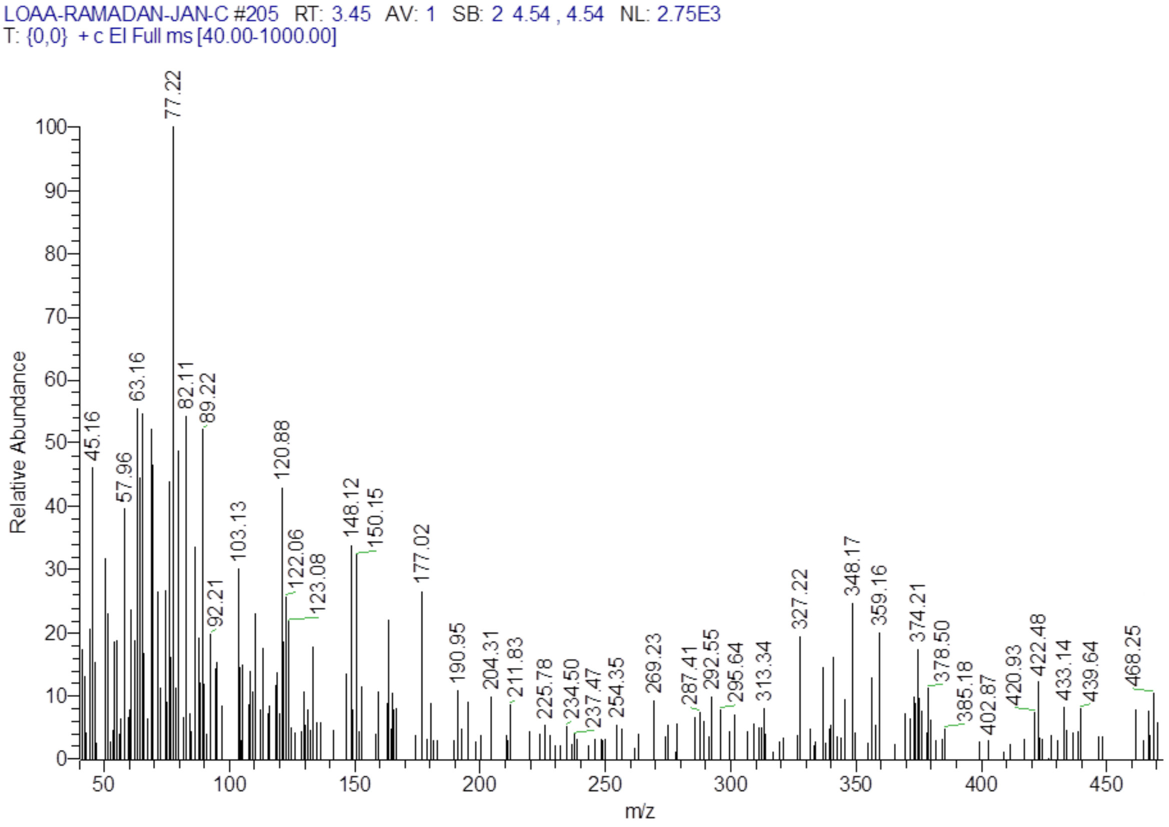

Compound 10

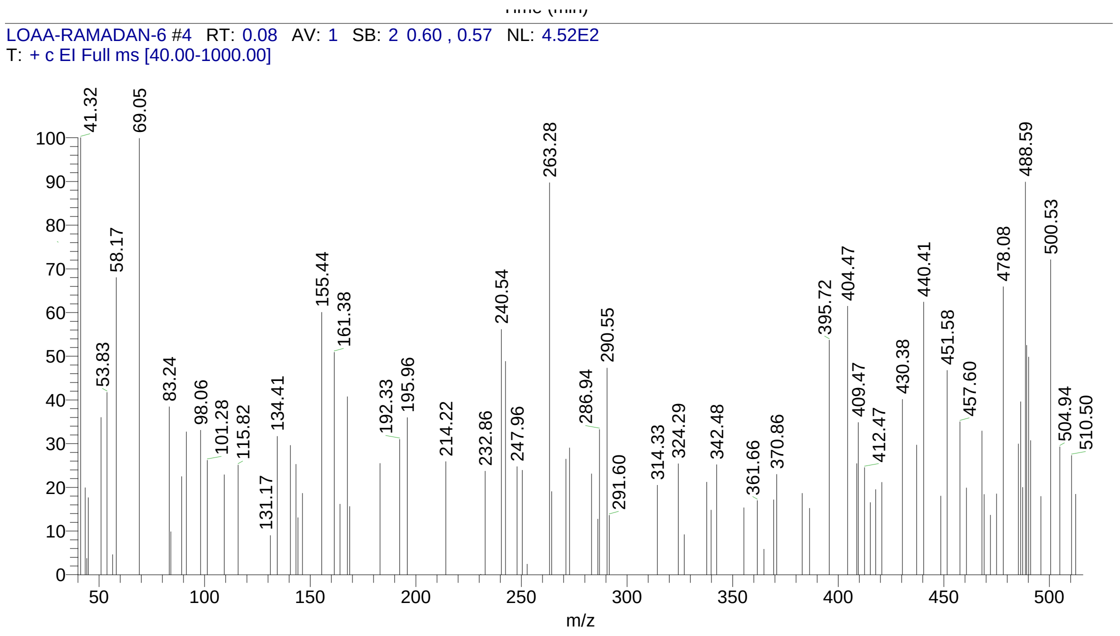

Compound 11

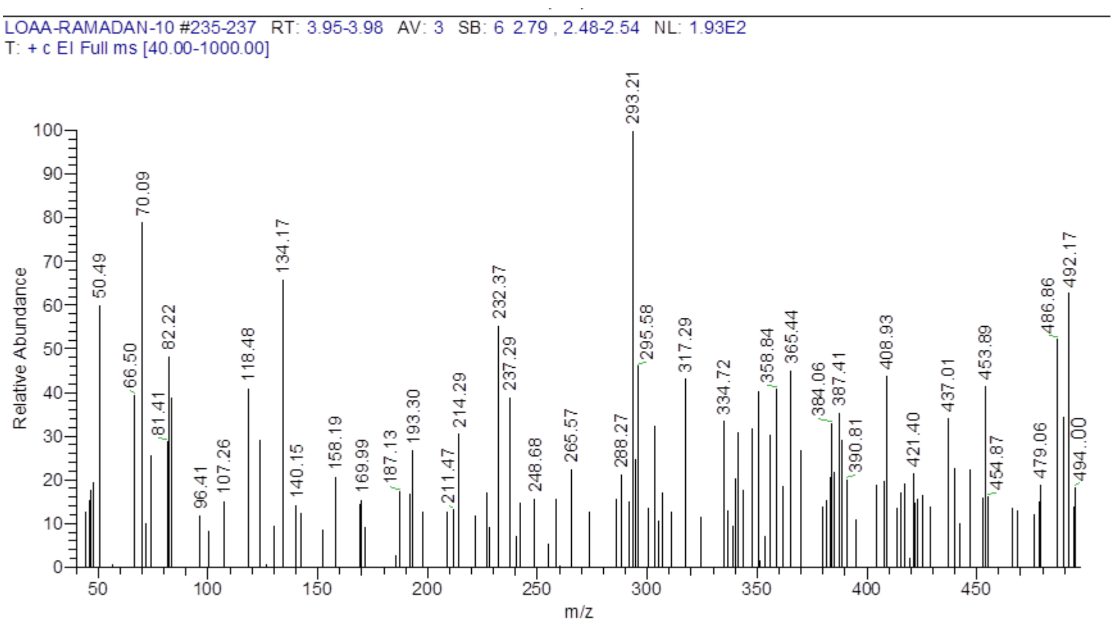

Compound 12

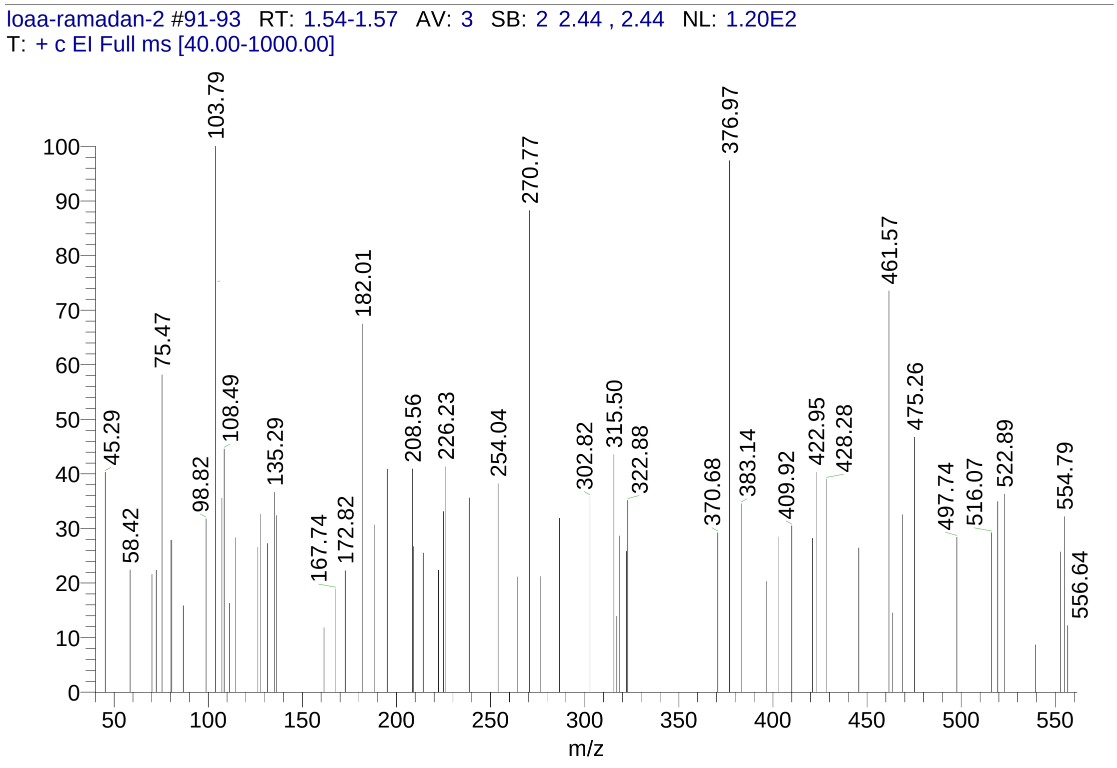

Compound 13

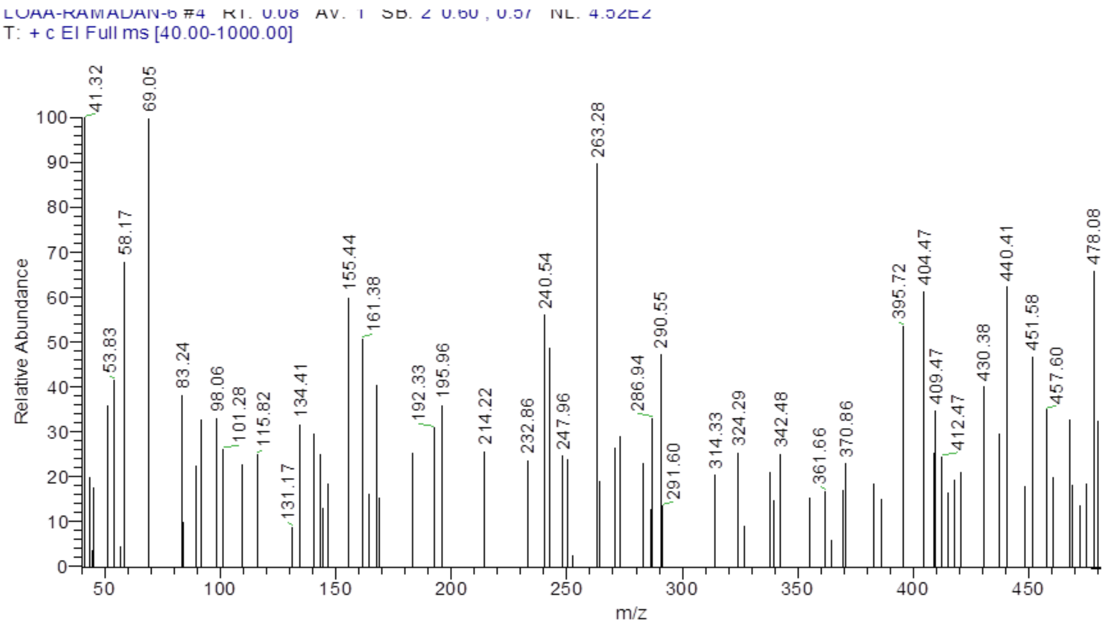

Compound 14

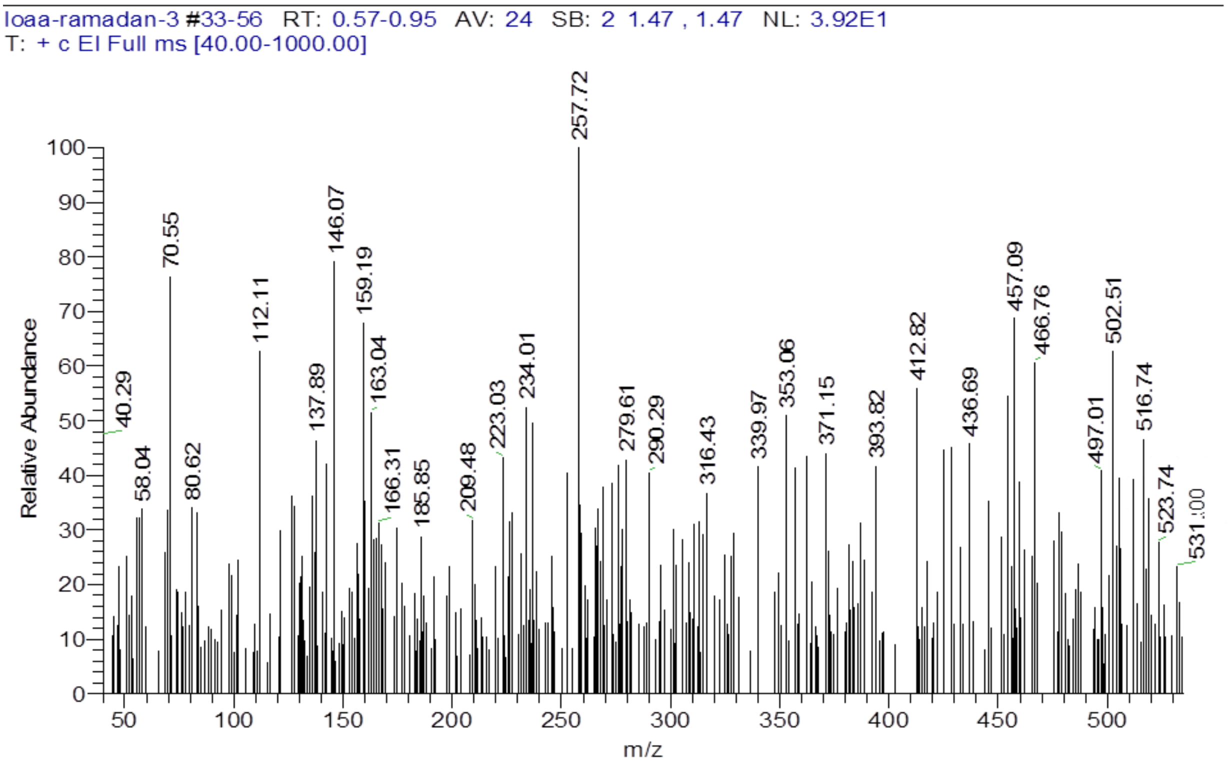

Compound 15

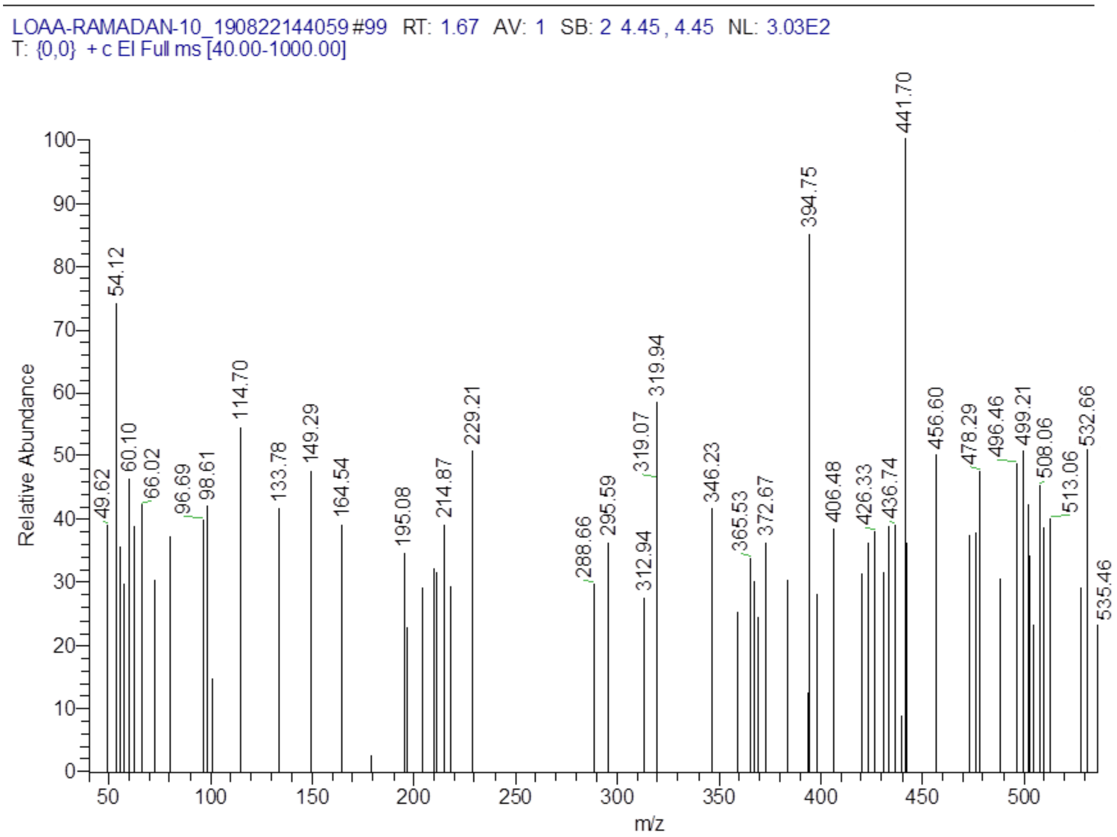

LOAA-RAMADAN-10_190822144059 #99 RT: 1.67 AV: 1 SB: 2 4.45 , 4.45 NL: 3.03E2
T: {0,0} + c EI Full ms [40.00-1000.00]
Relative Abundance
m/z
441.70
394.75
54.12
319.94
114.70
229.21
456.60
532.66
499.21
478.29
496.46
149.29
60.10
319.07
346.23
133.78
164.54
214.87
49.62
66.02
96.69
98.61
195.08
288.66
295.59
312.94
365.53
372.67
406.48
426.33
436.74
508.06
513.06
535.46
